# Metal Battery Anode: Rich in Electrons, Rich in Problems: A Critical Review from Industrial Perspectives

**DOI:** 10.1002/advs.76682

**Published:** 2026-07-30

**Authors:** Jian Pan, Chan Shu, Oscar Tutusaus, Rana Mohtadi

**Affiliations:** ^1^ Toyota Research Institute of North America (TRINA) Ann Arbor Michigan USA; ^2^ Advanced Institute for Materials Research (AIMR) Tohoku University Sendai Japan

**Keywords:** Li metal, liquid electrolytes, metal anode, Mg metal, Na metal, solid state electrolytes

## Abstract

Metal‐anode batteries promise energy densities beyond conventional Li‐ion. However, despite decades of study and earlier discontinued commercialization efforts, they still lag Li‐ion in efficiency, cycle life, and safety, leaving practical adoption uncertain. Key challenges stem from the metals’ inherent reactivity, incomplete understanding of interphase formation and evolution, and nonstandard testing and reporting that can mask true failure modes and misses to present performance relevant to practical use. Encouragingly, progress in electrolyte development and understanding of interphases over the past decade has uncovered more shared behavior across different metals than previously assumed, alongside important metal‐specific nuances. Thorough integration of these insights presents significant opportunities to deepen our knowledge and steer the field toward promising avenues for future advancements. This review unifies guiding principles for reactive monovalent (Li, Na) and multivalent (Mg, Ca) metal anodes across liquid and solid‐state electrolyte designs, summarizes major advances and barriers, and flags frequent pitfalls to guide the research community.

## Graphical abstract

Metal‐anode batteries using Li, Na, Mg, and Ca offer exceptionally high energy density, but dendrites, unstable interphases, cracking, and pore formation hinder durability and safety. This review shows how careful electrolyte and interphase design can stabilize these reactive metals. Drawing insights from monovalent and multivalent batteries in liquid and solid‐state systems, it identifies shared degradation mechanisms and strategies for safer, more efficient energy storage.

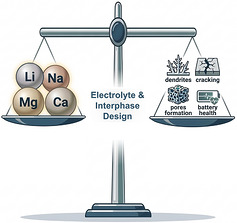

## 1 Introduction

Alkali and alkaline‐earth metal anodes are increasingly pursued as a route to batteries with energy densities beyond those of today's Li‐ion technology. Interest in these systems has surged in recent years, but roots of research on reversible metal anodes date back to the 1960s [1], and commercialization efforts involving lithium‐ or sodium‐based cells have been explored for several decades [2]. Despite this long history, practical deployment has been constrained by the metals’ high chemical reactivity and the difficulty of achieving long cycle life at room temperature. As a result, many concepts have either been shelved or confined to regimes where key failure modes are partially mitigated, such as high‐temperature molten‐metal batteries [2] or niche primary battery systems [3].

The benchmark for success is by and large set by Li‐ion batteries, whose dominance stems from their ability to cycle reversibly with high‐capacity retention up to thousands of cycles. The Li‐ion trajectory underscores both the scale of improvement required and why it can be worthwhile to pursue technically challenging systems with higher upside: when Sony introduced Li‐ion batteries commercially in 1991 to power their camcorders [4], costs were on the order of $5000 per kWh, with modest specific energy (120 Wh/kg), volumetric densities (264 Wh/L), and limited cycle life. Since then, the energy densities have more than doubled (to ≥270 Wh/kg and ≥650 Wh/L), while costs fell by ∼98% to roughly $101 per kWh by 2021 [5]. This history emphasizes that competitive technologies must ultimately demonstrate not only attractive intrinsic materials properties, but also durable, manufacturable performance and a credible path to cost reduction.

Metal‐anode research spans lithium and sodium, as well as multivalent systems such as zinc, magnesium, and calcium. With the exception of zinc, these metals are highly reactive in ambient air and can act as strong nucleophiles, complicating handling and long‐term interfacial stability. Their electrochemical redox reactions occur at very low potentials. Lithium, for example, has the lowest standard electrode potential at −3.04 V versus the Standard Hydrogen Electrode at 25°C. It is important to emphasize, although not sufficiently addressed, that the practical operating voltages can deviate markedly from these ideal values because overpotentials are proportional to current densities and tied up to electrolyte composition and the properties of interphases and interfaces. These realities place stringent demands on electrolyte design. Indeed, across metal types, room‐temperature rechargeable metal‐anode cells often fail through recurring mechanisms, including short circuits triggered by dendrite growth or metal penetration (including creep‐related phenomena), and in liquid‐electrolyte systems, progressive electrolyte depletion and drying driven by continuous parasitic reactions with the metal surface. These failure modes remain central barriers to commercialization.

Among the metals studied, lithium has received the most attention due to its exceptionally high theoretical capacity and low potential, making it a leading candidate for energy densities beyond graphite‐based Li‐ion anodes. This has motivated the frequent portrayal of lithium metal as a “holy grail” anode. Here, however, we emphasize that a more general objective would be more effective: the goal is not lithium metal per se, but any anode chemistry that can deliver a practical advantage over incumbent Li‐ion technology under real operating constraints. Achieving that advantage requires balancing high capacity with long cycle life, safety, manufacturability, and cost‐effectiveness; without this balance, strong theoretical metrics rarely translate into practical value.

Although mechanistic details vary, monovalent (Li, Na) and multivalent (Mg, Ca) metal anodes share common chemical and electrochemical themes [6]. In particular, a growing body of work suggests that broadly similar electrolyte frameworks and interphase‐engineering principles can often be translated across both classes—stabilizing the metal–electrolyte interface, mitigating dendrite‐driven failure, and tuning solvation/coordination environments through electrolyte formulation. Despite this convergence, the two fields have largely evolved separately, reflecting a long‐standing perception that each class demands fundamentally distinct electrolyte chemistry and design strategies.

Accordingly, while there are many excellent reviews covering advances in metal systems or specific components, a unified, comparative treatment that integrates guiding principles across multiple metals, evaluates both liquid and solid‐state battery systems within the same framework, and addresses practical needs, remains needed. This need is sharpened by the rapid expansion of the literature, coupled with inconsistent reporting and non‐standardized test conditions that complicate the interpretation of reported “breakthroughs” and hinder fair comparisons across studies [7, 8]. In this review, we consolidate lessons across metal systems, highlight the most consequential technical findings and barriers, and identify common pitfalls such as overemphasis on isolated performance metrics without sufficient consideration of long‐term stability, reproducibility, or scalability.

**FIGURE 1:**
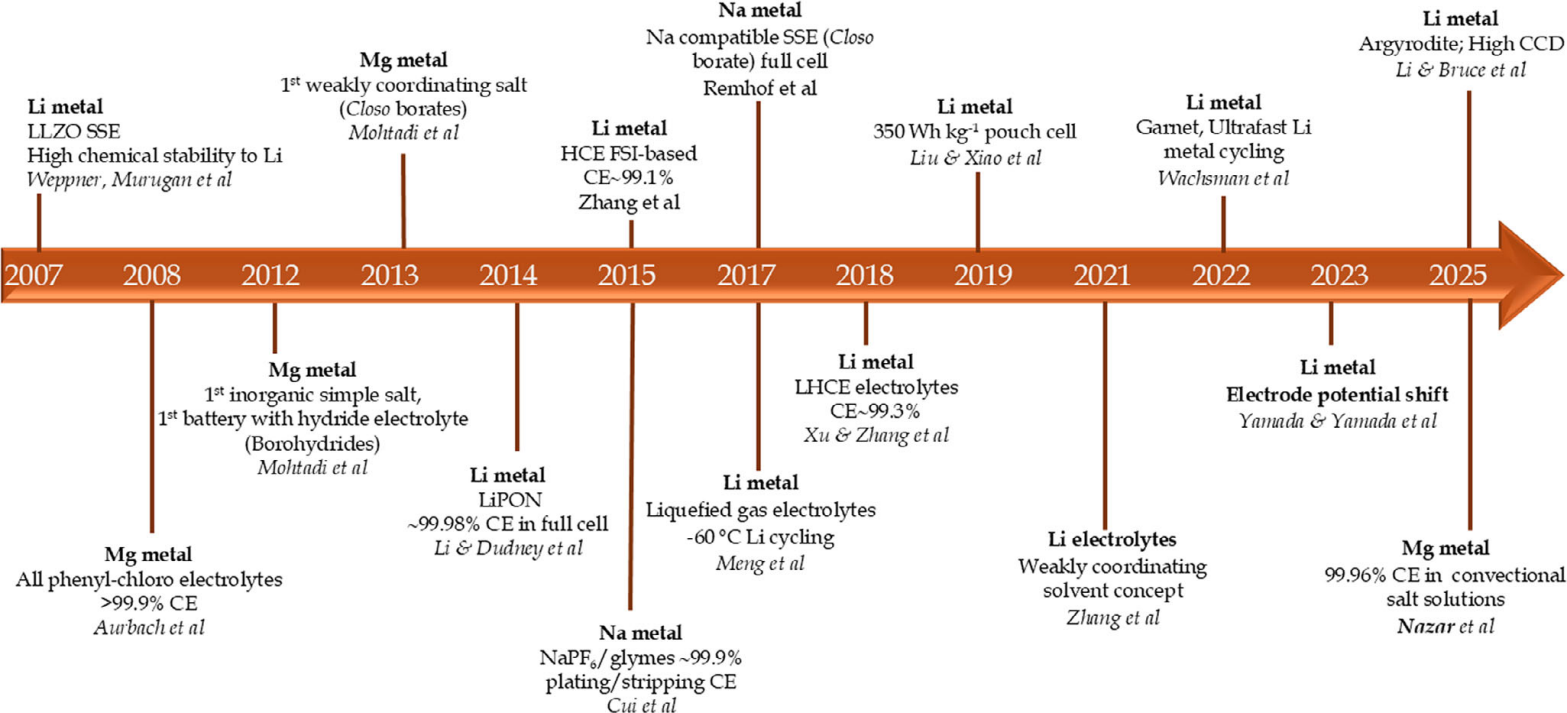
Consequential electrolyte designs and pivotal battery demonstrations that revealed the electrolyte's capabilities with metal anodes, driving and inspiring further advancements. In a chronological order: First demonstration of the garnet lithium lanthanum zirconium oxide LLZO solid state electrolyte and its low reactivity with Li metal by Weppner, Murugan et al. [9] Demonstration of ultrahigh Coulombic Efficiency CE in all‐phenyl MgCl electrolytes developed by Aurbach et al. This electrolyte enhanced the Mg battery operating voltage window [10]. Concept and first demonstration of simple inorganic salts, Mg(BH_4_)_2_/ethereal solutions that are chloride‐free and can cycle Mg metal efficiently [11]. Concept and first demonstration of weakly coordinating salts (closo‐borates) that efficiently cycle Mg metal and support increased battery operating voltage window by Mohtadi et al. [12, 13]. Demonstration of extremely efficient Li cycling in full cell employing lithium phosphorus oxynitride LiPON solid state electrolyte SSE by Li and Dudney et al. [14]. Demonstration of highly concentrated LiFSI/DME electrolytes by Zhang et al. [15]. Demonstration of high CE for Na plating/stripping in conventional 1 M NaPF_6_/glyme solutions by Cui et al. [16]. Demonstration of efficient cycling of Na_4_B_12_H_12_B_10_H_10_ solid electrolyte in Na metal in half and full cells by Remhof et al. [17, 18]. Demonstration of liquefied gas solvents for efficient low‐temperature Li cycling by Meng et al. [19]. Demonstration of the new concept of locally highly concentrated LHCE electrolytes for Li efficient half and full cell metal cycling at low concentration of LiFSI salt by Zhang and Xu et al. [20]. Demonstration of 600 cycles of Li metal pouch cell, 76% capacity retention by Lui and Xiao et al. [21]. Demonstration of weakly coordinating solvent concept to create anion‐rich solid electrolyte interphase by Zhang et al., proposed for Li‐ion and proven later effective for Li metal cycling [22]. Demonstration of ultrafast Li metal cycling in 3D electrode structure by Wachsman et al. [23]. Concept and demonstration of Li plating potential shift function of Li coordination and its impact on CE by Yamada and Yamada et al. [24]. Demonstration of the potential to achieve very high Critical Current Density CCD (∼9 mA/cm^2^ for Li plating/stripping in prototypical argyrodite Li_6_PS_5_Cl SSE through densification by Li & Bruce et al. [25]. Demonstration of high Mg plating/stripping CE using the conventional salt Mg triflate in ether, phosphate solutions by Nazar et al. [26].

This review centers on the performance of Li, Na, and Mg metal anodes and on how electrolytes and their interphase design govern their behavior. These relatively more mature systems have informed, and may ultimately help unlock, less studied metal anodes such as Ca and K. Tremendous advancement has been made in all the last two decades, and Figure 1 depicts consequential electrolytes designs and related battery demonstrations that have opened a new path. The article is structured into sections on Li metal in liquid electrolytes, Li metal in solid‐state electrolytes, Na metal anodes, and Mg metal anodes, conclusions and perspectives. Throughout the review, research findings are discussed from the standpoint of practical relevance where appropriate, with dedicated sections addressing key considerations, including the impact of metal cycling conditions and failure modes.

## 2 Lithium Metal Anode With Liquid Electrolytes

Lithium metal is an attractive anode material for next‐generation rechargeable batteries, owing to its exceptionally high theoretical specific capacity of 3860 mAh/g and lowest electrochemical potential (−3.04 V vs. SHE) among other metals. When paired with high‐voltage cathodes, lithium metal anodes offer the potential to dramatically increase cell‐level energy density, making them highly attractive for a broad range of applications, including electric vehicles, aviation, and grid‐scale energy storage.

Despite these advantages, the practical implementation of lithium metal batteries (LMBs) remains challenging due to several critical issues. These include uncontrolled lithium dendrite growth, low Coulombic efficiency (CE), continuous electrolyte consumption, and limited cycle life. These problems primarily arise from the unstable and reactive interface between lithium metal and conventional liquid electrolytes, which leads to the formation of non‐uniform solid electrolyte interphase (SEI) layers and the accumulation of inactive lithium (dead Li), ultimately compromising battery safety and performance.

Liquid electrolytes play a pivotal role in determining the electrochemical behavior of lithium metal anodes. They influence the solvation structure of Li^+^ ions, govern the formation and composition of the SEI, and affect the morphology of lithium deposition. Over the past decade, significant research efforts have focused on engineering advanced liquid electrolyte formulations to stabilize lithium cycling. Key strategies include the development of high‐concentration electrolytes (HCEs), localized high‐concentration electrolytes (LHCEs), weakly solvating electrolytes (WSE), fluorinated solvents and diluents, multi‐salt systems, and tailored additive packages. These approaches aim to promote the formation of robust, LiF‐rich SEI layers that suppress dendrite growth, enhance Coulombic efficiency, and improve compatibility with high‐voltage cathodes.

This section summarizes key recent advances in liquid electrolyte engineering for lithium metal batteries, emphasizing electrolyte composition, solvation chemistry, SEI structure, and electrochemical performance. We also address critical challenges such as cost, scalability, and safety, and explore future directions toward practical LMBs. By providing a state‐of‐the‐art overview, this section seeks to guide the rational design of next‐generation liquid electrolytes toward safe, durable, and high‐energy lithium metal batteries. In addition, Table 1 summarizes the Li CE of electrolytes that are discussed. The CE was determined using either the cycling method or the Aurbach method at different rates and capacities. The Aurbach method provides a quick evaluation of lithium cycling performance, while the cycling method reveals the long‐term stability of the electrolyte. For the cycling method, the reported CE for some electrolytes wasn't calculated starting from the initial cycles, excluding the losses associated with the formation of a stable SEI. The first‐cycle CE values are also included in the table (obtained from reported cycling profiles). Notably, although some electrolytes show average CE values above 99.0%, their first‐cycle CEs fall below 90%, which indicates excessive reactivity, inefficient SEI and isolated metal formation that may impact performance in subsequent cycles. Although CE values above 99.0% were achieved for some electrolytes, significant CE fluctuations of up to ±1.5% were observed directly in the cycling profiles. These large fluctuations suggest possible SEI instability and ongoing side reactions. Therefore, when reporting high CE for novel electrolytes, it is crucial to carefully consider the full cycling performance, including CE stability over extend cycles, to ensure reliable electrolyte behavior.

**TABLE 1:** Li CE of selected reported electrolytes in Li|Cu half cells.

Electrolyte formulation	Year	Method	Current to capacity	Cycle	First‐cycle CE	CE (accounted cycles)	CE fluctuation
	Carbonate‐based electrolytes						
3.27 mol/kg LiBETI in PC [27]	2008	Cycle	0.5 mA/cm^2^ to 0.25 C/cm^2^	50	—	∼80%	—
10 M LiFSI in DMC [28]	2018	Cycle	0.2 mA/cm^2^ to 1 mAh/cm^2^	200	∼90%	99.2% (160–200)	∼0.5%
10 M LiFSI in EC/DMC [28]	2018	Cycle	0.2 mA/cm^2^ to 1 mAh/cm^2^	250	∼89%	99.3% (100–250)	∼±1%
7 M LiFSI in FEC [29]	2018	Cycle	0.25 mA/cm^2^ to 0.5 mAh/cm^2^	400	89%	99.6% (300–400)	∼±1.5%
1.2 M LiFSI/DMC‐BTFE (1:1.5 by mol) [30]	2018	Aurbach	0.5 mA/cm^2^ to 1 mAh/cm^2^	10	—	99.3%	—
1.2 M LiFSI in TEP/BTFE [31]	2018	Aurbach	0.5 mA/cm^2^ to 1 mAh/cm^2^	10	—	99.2%	—
1.2 M LiFSI with 0.15 M LiBF_2_(C_2_O_4_) (LiDFOB) in EC/EMC/BTFE [32]	2018	Cycle	0.5 mA/cm^2^ to 1 mAh/cm^2^	200	−85%	98.2% (0–160)	±1%
1 M LiPF_6_ in FEC/FMEC/HFE [33]	2018	Cycle	0.2 mA/cm^2^ to 1 mAh/cm^2^	500	∼95%	>99.2% (50–500)	—
1 M LiPF_6_ in VEC [34]	2020	Cycle	0.5 mA/cm^2^ to 0.5 mAh/cm^2^	1400	—	98.4%	±1%
1 M LiPF_6_ in BTC‐FEC [35]	2022	Cycle	0.5 mA/cm^2^ to 1 mAh/cm^2^	300	96%	98.8% (10–300)	<±0.5%
	Ether‐based electrolytes						
4 M LiFSI in DME [15]	2015	Cycle	0.2 mA/cm^2^ to 1 mAh/cm^2^	500	—	99.1%	—
1LiFSI‐1.2DME‐3TTE (1.5 M LiFSI in DME/TTE) [20]	2019	Cycle	0.5 mA/cm^2^ to 1 mAh/cm^2^	300	∼98.5%	99.3%	±1%
1 M LiFSI in DME‐TFEO [36]	2019	Aurbach	0.5 mA/cm^2^ to 1 mAh/cm^2^	10	—	99.5%	—
1 M LiFSI in FDME [37]	2020	Cycle	0.5 mA/cm^2^ to 1 mAh/cm^2^	300	97.6%	99.3% (5–300)	<±0.5%
		Aurbach	0.5 mA/cm^2^ to 1 mAh/cm^2^	10	—	99.52%	—
1 M LiFSI in DEE [38]	2021	Aurbach	0.5 mA/cm^2^ to 1 mAh/cm^2^	10	—	98.9% at RT 98.4% at −60°C	—
1 M LiFSI in 1,2‐DEE [39]	2021	Cycle	0.5 mA/cm^2^ to 0.5 mAh/cm^2^	100	76%	98.0%	—
4 M LiFSI in 1,2‐DEE [40]	2021	Cycle	0.5 mA/cm^2^ to 1 mAh/cm^2^	150	∼98%	99.25%	—
2 M LiFSI in DBE [41]	2022	Aurbach	0.5 mA/cm^2^ to 1 mAh/cm^2^	10	—	99.0% at RT 98.0% at −50°C	—
1 M LiFSI in DMM [42]	2022	Aurbach	0.5 mA/cm^2^ to 0.5 mAh/cm^2^	10	—	99.12%	—
1.5 M LiFSI in DMM [24]	2022	Cycle	0.5 mA/cm^2^ to 0.5 mAh/cm^2^	400	∼94%	99.1%	<±0.5%
1.2 M LiFSI in F5DEE [43]	2022	Cycle	0.5 mA/cm^2^ to 1 mAh/cm^2^	600	∼97.5%	99.90% (100–600)	<±0.1%
		Aurbach	0.5 mA/cm^2^ to 1 mAh/cm^2^	10	—	99.5%	—
1.2 M LiFSI in F4DEE [43]	2022	Aurbach	0.5 mA/cm^2^ to 1 mAh/cm^2^	10	—	99.5%	—
1 M LiFSI in DBE‐toluene [44]	2023	Aurbach	0.5 mA/cm^2^ to 1 mAh/cm^2^	10	—	99.70%	—
1 M LiFSI in MTBE‐toluene [44]	2023	Aurbach	0.5 mA/cm^2^ to 1 mAh/cm^2^	10	—	99.64%	—
LiFSI‐2CPME [45]	2023	Aurbach	0.5 mA/cm^2^ to 0.5 mAh/cm^2^	10	—	99.4%	—
		Cycle	0.5 mA/cm^2^ to 1 mAh/cm^2^	350	∼95%	99.3%	∼±0.25%
	LCEs						
0.3 M LiTFSI, 0.3 M THF in FM:CO_2_ 19:1 [46]	2019	Cycle	0.5 mA/cm^2^ to 0.5 mAh/cm^2^	500	∼95%	99.9% (100–500)	∼±1%
1.2 M LiTFSI, 1 M AN in FM:CO_2_ 19:1 [47]	2020	Cycle	0.5 mA/cm^2^ to 0.5 mAh/cm^2^	200	∼95%	99.4%	—
	Other novel electrolytes						
2.5 M LiFSI, 0.2 m LiPF_6_ in FSA [48]	2021	Cycle	0.5 mA/cm^2^ to 0.5 mAh/cm^2^	400	∼91%	99.03%	∼±1%
1 M LiFSI in DMTMSA [49]	2021	Cycle	0.5 mA/cm^2^ to 1 mAh/cm^2^	350	∼90%	99%	∼±1%

### 2.1 Electrolyte Engineering Guiding Principles

**FIGURE 2:**
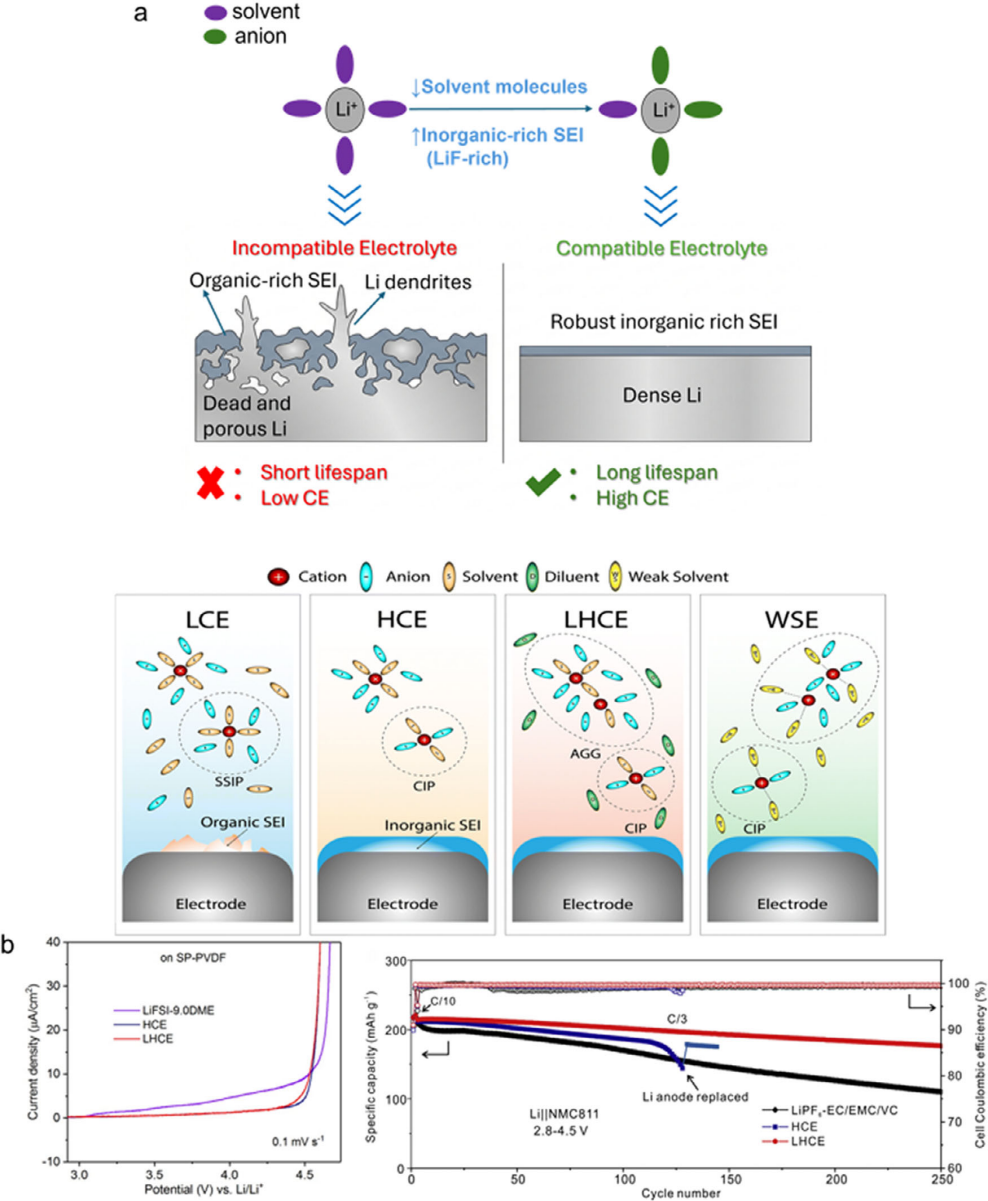
(a) Schematic of electrolyte engineering for LMBs, and schematic of four types of solvation structures. Schematic of four solvation structures. Adapted with permission [60]. Copyright 2025, Royal Society of Chemistry. (b) LSV and cycling performance of Li|NMC811 cells using different electrolytes, HCE: 4M lithium bis(fluorosulfonyl)imide (LiFSI) in DME, LHCE: 1.5 M LiFSI in DME/ 1,1,2,2‐tetrafluoroethyl‐2,2,3,3‐tetrafluoropropyl ether (TTE). Reproduced with permission [20]. Copyright 2019, Elsevier.

Electrolyte engineering involves the deliberate design and optimization of electrolyte composition and properties to enhance the performance, stability, and safety of electrochemical energy storage systems, particularly LMBs. This approach focuses on tailoring the chemical formulation—including salts, solvents, and additives—to control key factors such as ionic conductivity, electrochemical stability, solvation structure, and interfacial reactions. In LMBs, electrolyte engineering aims to modulate the SEI chemistry to form a stable, mechanically robust passivation layer that suppresses lithium dendrite growth and minimizes parasitic side reactions. By optimizing the solvation environment of lithium ions, it influences transport kinetics and SEI composition, thereby improving cycling stability and battery performance.  The key guiding principles of electrolyte engineering for LMBs are reducing the number of solvent molecules in the Li^+^ solvation sheath and promoting the formation of an inorganic‐rich, mainly LiF‐containing SEI layer. This is currently achieved using highly concentrated electrolytes (HCEs), localized highly concentrated electrolytes (LHCEs), and weakly solvating electrolytes (WSEs) (Figure 2a). The common feature in all these systems, as discussed next, is that Li^+^‐anion interactions are enhanced through the overall minimization of the coordinating solvent content (HCE, LHCE) or utilization of more weakly coordinating solvents (WSE).

Similar to lithium ion batteries (LIBs) [50], the typical desired salt concentration in LMBs is around 1.0 M to optimize ionic conductivity, viscosity, and salt solubility. At this concentration, solvent‐separated ion pairs (SSIPs) predominate due to the abundance of free solvent molecules. However, a high proportion of solvent molecules in the solvation structure tends to produce unstable, organic‐rich SEI, which accelerates lithium dendrite growth and degrades cycling performance. Increasing salt concentration leads to the formation of HCEs, where the number of free solvent molecules is significantly reduced. This strengthens cation–anion interactions and promotes the formation of contact ion pairs (CIPs) and larger ion aggregates (AGGs). The concept of HCEs originated from LIBs research. In 1985, Mckinnon and Dahn first demonstrated that a saturated solution of LiAsF_6_ in propylene carbonate (PC) could eliminate the co‐intercalation of PC in ZrS_2_ electrode [51]. In 2003, Jeong and co‐workers reported that concentrated LiN(SO_2_C_2_F_5_)_2_ (LiBETI, 2.72 M) in PC enabled lithium‐ion intercalation into graphite without graphite exfoliation caused by PC co‐intercalation [52]. In 2010, Yamada and co‐workers showed that concentrated Lithium bis(trifluoromethanesulfonyl)imide (LiTFSI) in dimethyl sulfoxide (DMSO) similarly prevented the co‐intercalation of solvent into graphite [53]. These studies collectively demonstrated that high‐concentration electrolytes mitigate solvent effects and improve cell performance. Concurrently, Watanabe and co‐workers found that highly concentrated Li(G3)TFSI electrolytes in triglyme (G3) exhibited unique properties compared to dilute solutions, including enhanced thermal and oxidative stability, and supported stable Li ion intercalation into graphite without solvent co‐intercalation [54, 55]. In 2014, Yamada and co‐workers further reported improved reductive stability and ultrafast charging characteristics in LIBs using highly concentrated LiTFSI in acetonitrile (AN) [56]. Spectroscopic analyses (X‐ray photoelectron spectroscopy and Raman spectroscopy) and molecular dynamics simulations (DFT‐MD) confirmed that HCEs reduce the number of solvent molecules coordinated to Li^+^, thereby eliminating solvent co‐intercalation and enhancing electrolyte reductive stability. In 2015, Wang and Xu's groups introduced a highly concentrated “water‐in‐salt” electrolyte (WiSE) based on approximately 5 M LiTFSI in water, enabling the first high‐voltage aqueous LIB [57].

These findings from LIBs research highlight that high‐concentration electrolytes effectively modify solvation structures and provide valuable guidance for advancing electrolyte design in LMBs and informed the design of electrolytes for LMB [15]. The elevated salt concentration of HCEs modifies the solvation structure and suppresses solvent activity, promoting the formation of LiF‐rich interphases that stabilize the lithium metal anode. In recent decade, numerous HCE formulations have been explored. However, the practical use of HCEs is limited by their high viscosity, poor wettability with electrodes and separators, reduced ionic conductivity, and high cost. To overcome these challenges, diluents are typically added to form LHCEs [58]. These diluents are non‐coordinating and miscible with coordinating solvents in the electrolytes that preserve the advantageous solvation properties of HCEs while enhancing their physical characteristics. A micelle‐like structure has been proposed for LHCE, featuring a higher concentration of AGGs at the core, surrounded by diluents that are miscible with the solvents, which effectively increases the local salt concentration [59]. Both HCEs and LHCEs have been validated as effective strategies to enhance the cycling performance and stability of lithium metal batteries in a variety of solvents. Notably, unlike with diluent electrolytes (usually salt concentration = 1M), Al corrosion is suppressed when using electrolytes containing imide‐based salts in HCEs and LHCEs [58]. In addition, improved oxidative ability can be achieved with few free solvent molecules in the electrolytes. This is especially important for ether‐based electrolytes used in high‐voltage battery applications, as ethers are typically unstable above 4 V (vs. Li/Li^+^). For example, 1,2‐dimethoxyether (DME)‐based LHCEs can remain stable up to 4.5 V (vs. Li/Li^+^) (Figure 2b) [20].

The selection of diluents for LHCEs is limited because they must be non‐ or weakly coordinating to Li^+^ and miscible with the solvents. Most diluents are highly fluorinated solvents [61], which tend to be costly and environmentally unfriendly. Additionally, LHCEs generally exhibit low conductivity due to strong ion pairing, and their performance is sensitive to the solvent‐to‐diluent ratio, restricting their effectiveness during fast charging or low‐temperature operation. Recently, weakly solvating electrolytes (WSEs) have emerged as an alternative electrolyte design strategy, and several comprehensive reviews have discussed the development and design of WSEs [60, 62, 63]. The solvents in WSEs, known as weakly solvating solvents, possess low donor numbers (DNs) and low dielectric constants ɛ [22]. Despite these properties, they can still dissolve salts while interacting only weakly with metal ions. In these solvation structures, the competition between anions and solvent molecules for Li^+^ favors anions, leading to an increased presence of CIPs and AGGs. As a result, WSEs can sustain an anion‐derived SEI even at moderate or low salt concentrations (e.g., 1 M). In the following section, we will also cover key electrolytes and solvent molecule designs within WSEs.

The studies of HCE and LHCE have contributed to generating the two key principles for designing competent electrolytes for lithium metal anode (LMA) to enable a CE greater than 99% [64]. The first aims to minimize unexpected solvent decomposition on lithium by minimizing its content while the other aims to drive the formation of an inorganic‐rich SEI layer—such as a LiF‐rich SEI—to suppress lithium dendrite growth and improve lithium reversibility. However, a significant gap remains between designing optimal electrolytes to achieve lithium CE above 99.9% or even 99.99% and the fundamental theoretical understanding. It is essential to clarify how different electrolytes influence cycling performance. Recently, efforts have been directed towards closing this gap. For example, a seminal report by Yamada's group recently demonstrated a large shift (>0.6 V) in the Li electrode potential (*E*_Li_) associated with Li^+^ coordination structures in LiFSI electrolytes [24]. LiFSI electrolytes with high *E*_Li_ indicate a weakened reducing environment, which can minimize the reductive decomposition of the electrolyte and may ultimately result in a higher CE. By summarizing numerous CE of LiFSI electrolytes, including LCEs, HCEs, LHCEs, and WSEs, they found that enhanced *E*_Li_ corresponds to increased CE. Machine learning‐based regression analysis shows that *E*_Li_ is strongly affected by Li^+^–FSI^−^ interactions. Raman spectroscopy supports this finding, showing that structures with more AGGs and CIPs exhibit higher *E*_Li_ and CE. This work shows that the CE of LiFSI electrolytes can vary significantly despite having similar FSI^−^‐derived SEI layers, and that increasing *E*_Li_ can lead to improved CE. It should be, however, noted that a follow‐up paper from the same group suggested the increased *E*_Li_ of the electrolyte also decreases its cathodic stability, making it challenging to use with high‐voltage cathode [65].

Competent dilute LiFSI solutions are not necessarily limited to the presence of fluorine‐containing solvents. For example, reduction in the solvent oxygen content as informed from machine learning (ML) model resulted in high CE [44]. Considering the key features of advanced electrolytes, elemental composition descriptors were included in the ML model. Among these, the solvent oxygen ratio (sO), fluorine to oxygen ratio (F/O), inorganic to organic ratio (InOr), and anion carbon ratio (aC) were identified as the most relevant features. Importantly, model analysis revealed that reducing solvent oxygen content is the most critical factor improving electrolyte performance. This effect may be attributed to the reduced oxygen content decreasing the interaction between Li^+^ and the solvents, thereby promoting the formation of FSI^−^‐derived SEI layers. In a broader sense, what is effective is achieving a balanced Li ion–solvent affinity that supports ion‐pair association while maintaining salt dissociation, and modest anion–solvent affinity to stabilize CIPs and AGGs. These interactions can be quantified and compared through the normalized cation/anion–solvent affinity, which can help down‐select appropriate salt‐solvent systems [66].

All the above strategies mainly use LiFSI as the salt. LiFSI as a salt in lithium metal batteries offers several advantages, including comparable ionic conductivity to LiPF_6_, enhanced thermal stability, and the promotion of a stable, uniform SEI layer that reduces dendrite growth and improves cycling stability [67, 68]. However, LiFSI is significantly more expensive than traditional salts like LiPF_6_. In 2025, the price of battery‐grade LiFSI ranged from $40 000 to $70 000 per ton [69], compared to $6000 to $10 000 per ton for battery‐grade LiPF_6_ [70], making it less attractive from a cost perspective. Additionally, LiFSI is sensitive to moisture, requiring careful handling, has limited commercial availability, and may cause side reactions under certain conditions that could compromise long‐term battery stability. LiFSI salts in traditional carbonate electrolytes exhibit corrosion issues toward Al current collectors because they struggle to form a stable passivating layer such as Al_2_O_3_ and AlF_3_ [71, 72, 73]. This problem becomes more severe when the cut‐off voltage exceeds 4.2 V (vs. Li^+^/Li). Notably, Al corrosion has been observed at as low as 3.3 V (vs. Li^+^/Li) in 0.85 M LiFSI in EC/DME electrolytes [71]. Interestingly, trace amounts of chloride ions (Cl^−^) in LiFSI can significantly influence Al corrosion [72, 74]. In 1 M LiFSI in ethylene carbonate (EC)/ethyl methyl carbonate (EMC) electrolyte containing 50 ppm LiCl, the corrosion potential was measured at 3.7 V (vs. Li^+^/Li), and the corrosion current density remained elevated even after five cycles, indicating the absence of a protective passivation layer on the Al foil. In contrast, when the LiCl concentration was reduced to 0.45 ppm, although the corrosion potential remained unchanged, the corrosion current density decreased significantly after just one cycle, suggesting improved passivation and reduced Al corrosion [72]. Residual Cl^−^ can be introduced during the synthesis of LiFSI [72]; therefore, the purity of LiFSI used in battery electrolytes must be exceptionally high.

### 2.2 Electrolytes Based on Solvent Systems

#### 2.2.1 Carbonate Based

Carbonate‐based electrolytes possess wide electrochemical stability windows and exhibit excellent compatibility with high‐voltage cathodes (>4.2 V) and graphite anodes, which have enabled their successful commercialization in LIBs. However, these traditional carbonate solvents suffer from poor reductive stability when in contact with lithium metal, making it difficult to form a stable SEI [75, 76]. This instability results in low CE and a shortened cycling lifespan in LMBs. Research on diluent carbonate electrolytes with high cycling performance remains limited, as it is challenging to develop compatible carbonate‐based electrolytes at dilute concentrations that maintain excellent cycling stability. For example, Li's group reported a non‐flammable electrolyte consisting of 1 M LiPF_6_ in vinylethylene carbonate (VEC), achieving a lithium cycling CE of only 98.1% over 1400 cycles [34]. Consequently, extensive efforts have focused on engineering carbonate‐based liquid electrolytes to improve their interfacial stability with lithium metal. Strategies include the use of electrolyte additives, solvent blends, and novel salt formulations aimed at promoting stable SEI formation, enhancing lithium deposition uniformity, and ultimately extending battery cycle life. In addition, HCEs and LHCEs have also been widely developed in carbonate‐based electrolytes, which can effectively widen the electrochemical stability window and improve the battery performance. In 2008, Jeong's group first demonstrated high concentrations of 3.27 mol kg^−1^ LiN(SO_2_C_2_F_5_)_2_ (LiBETI) in PC could extend cycle life from 10 to 50 cycles at 0.5 mA/cm^2^ to 0.25 C/cm^2^ by suppressing the lithium dendritic growth; however, a modest CE of only about 80% could be achieved [27]. On the other hand, lithium cycling CE of 99.2% was shown in 10 M LiFSI in DMC and 99.3% with EC‐DMC mixture (0.2 mA/cm^2^, 1 mAh/cm^2^) [28]. The high concentration of FSI^−^ promotes the formation of LiF‐rich SEI layer. Moreover, fluorinated solvents and diluents have been incorporated into HCEs and LHCEs [61, 77]. Specifically, fluorine substitution lowers both the highest occupied molecular orbital (HOMO) and the lowest unoccupied molecular orbital (LUMO) levels simultaneously [78]. The decreased HOMO level improves oxidative stability against high‐voltage cathodes, while the lowered LUMO facilitates the reductive decomposition of the fluorinated solvent, leading to the formation of a LiF‐rich SEI. For example, fluoroethylene carbonate (FEC), a widely used additive in LIBs, was also used in LMBs [79, 80]. The decomposition of FEC generates a LiF‐rich SEI, which improves the cycling performance of carbonate‐based electrolytes in LMBs [81]. A 7 M LiFSI in FEC electrolyte had a CE of 99.6% (0.25 mA/cm^2^ to 0.5 mAh/cm^2^); however, it required approximately 300 cycles for the CE to gradually increase and stabilize [29]. Similarly, a 99.3% CE was reported in 1.2 M LiFSI solution in DMC/BTFE bis(2,2,2‐trifluoroethyl) ether (0.5 mA/cm^2^ using a modified Aurbach method for Li‐metal cycling) [30]. Li||NMC111 full cell maintained 80% capacity retention after 700 cycles when charged at C/2 (1C = 2.0 mA/cm^2^) and discharged at 2C. Ab initio molecular dynamics (AIMD) simulations and Raman spectroscopy revealed that BTFE did not participate in the solvation structure. The high electrolyte performance was attributed to the formation of a robust FSI‐derived SEI layer. Similar improvements BTFE as a diluent were reported in other solutions such as 1.2 M LiFSI in triethyl phosphate (TEP)/BTFE and 1.2 M LiFSI with 0.15 M LiBF_2_(C_2_O_4_) (LiDFOB) in BTFE [31, 32]. Beyond the LiFSI use, Wang's group reported that 1 M LiPF_6_ in FEC/3,3,3‐fluoroethylmethyl carbonate/1,1,2,2‐tetrafluoroethyl‐2’,2’,2’‐trifluoroethylmethyl (FEC:FEMC:HFE, 2:6:2 by weight) enabled a high CE of 99.2% for Li‐metal cycling (0.2 mA/cm^2^, 1 mAh/cm^2^) [33]. This electrolyte formed a robust SEI layer and significantly suppressed lithium dendrite growth, as evidenced by SEM images showing notably less cracking and dendritic formation in the deposited lithium layer compared to 1 M LiPF_6_ in EC/DMC. Additionally, a 5 V Li|LCP full cell maintained 93% capacity retention after 1000 cycles when cycled at 1C (1C = 2.0 mA/cm^2^).

Solvent modifications were utilized to weaken the interaction between Li^+^ and the solvent by reducing the electron density around the oxygen atoms. This combined to the use of fluoro‐carbonates resulted in substantial amounts of LiF in the SEI layer [33, 35]. For example, CF_3_ groups at both ends of diethyl carbonate (DEC)’s (formation of bis(2,2,2‐trifluoroethyl) carbonate BTC) resulted in a Li CE of 98.8% (1 M LiPF_6_ in BTC/FEC solution) over 300 cycles (0.5 mA/cm^2^, 1 mAh/cm^2^) [35]. Like other fluorinated solvents, BTC also demonstrates good oxidative stability. This electrolyte significantly enhanced the high‐voltage stability, enabling NCM811 cathode operation at ultrahigh cut‐off voltages up to 4.8 V (vs. Li/Li^+^). A Li||NCM811 cell cycled with a 4.7 V cut‐off maintained 95.1% capacity retention after 160 cycles at 0.5C (1C = 200 mA/g^2^). The ability to achieve CE beyond 99% in carbonate‐type solvents is indeed an interesting milestone; however, these systems suffer from severe outgassing issues and limited cyclic stability, which makes them less suitable than other solvents such as ethers.

#### 2.2.2 Ether‐Based Electrolytes

##### 2.2.2.1 Highly Concentrated and Locally Highly Concentrated Electrolytes HCEs and LHCEs

In 2015, Zhang's group from Pacific Northwest National Laboratory (PNNL) reported a high lithium cycling CE of 99.1% at 0.2 mA/cm^2^ and 98.5% at 1 mA/cm^2^ with 1 mAh/cm^2^ cycle capacity using 4 M LiFSI in DME over 500 cycles [15]. In this formulation, LiFSI was selected for its high ionic conductivity and solubility. The elevated salt concentration substantially decreased the fraction of uncoordinated DME and promoted strong coordination of FSI^−^ to Li^+^. This altered solvation structure enhanced both reductive and oxidative stability and fostered the formation of inorganic‐rich SEIs. As a result, the electrolyte facilitated Li deposition in large, uniform granules with improved cycling efficiency and was compatible with high‐voltage cathodes such as NMC. These findings spurred subsequent efforts to optimize HCEs for LMBs [28, 29]. In addition, PNNL reported that the electrolyte with a molar ratio of 1LiFSI‐1.2DME‐3 TTE (1.5 M LiFSI in DME/TTE) achieved impressive LMB cycling performance, demonstrating a Li CE of 99.3% (0.5 mA/cm^2^ with 1.0 mAh/cm^2^) over 300 cycles, along with uniform lithium deposition [20]. Although this high CE was attained, significant CE fluctuations of 1%–2% were observed during cycling, indicating that considerable side reactions still occurred. Using this electrolyte, Li||NMC811 coin cell with limited excess Li (N/P ratio = 2.4) was able to maintain 80% capacity at C/3 charge/discharge (1C = 4.2 mA/cm^2^) condition over 155 cycles. Consequently, in the same year, another LHCE, consisting of 1 M LiFSI in DME‐TFEO was reported, which demonstrated an excellent Li CE of 99.5% (modified Aurbach method at 0.5 mA/cm^2^ with 1 mAh/cm^2^) [36]. TFEO has a high boiling point of 143°C, and its three strong electron‐withdrawing ─CF_3_ groups enhance its oxidative stability. A Li|NMC811 cell with 1 M LiFSI in DME‐TFEO had an 80% capacity retention over 300 cycles cycled at C/3 (1C = 1.5 mA/cm^2^).

##### 2.2.2.2 Weakly Coordinating Electrolytes WSEs

**FIGURE 3:**
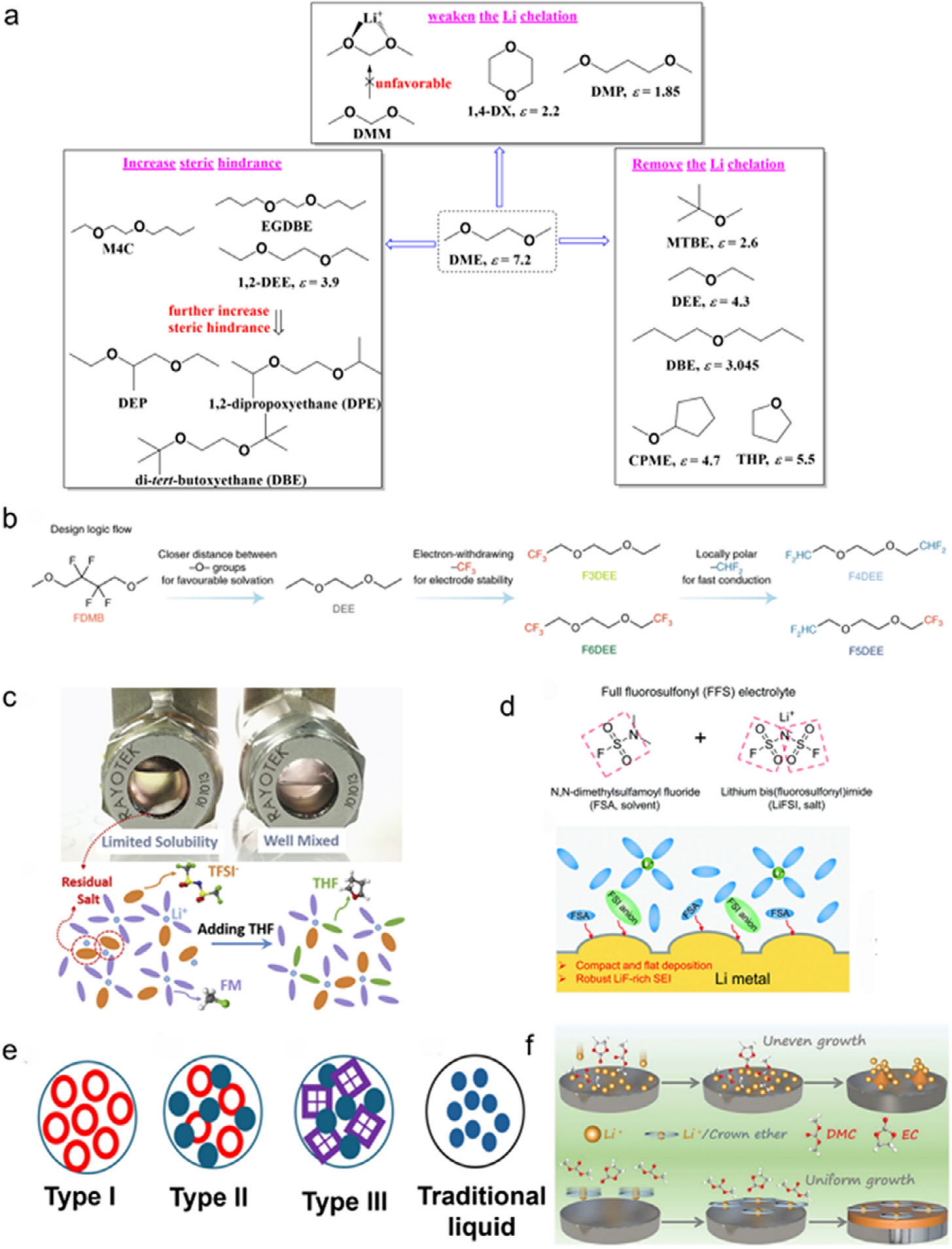
(a) The design principles and structures of weakly solvating solvents based on DME. (b) Structures and design principles of FxDEE, x = 4–6. Reproduced with permission [43]. Copyright 2022, Nature Publishing Group. (c) Schematic illustration of the solvation mechanism of the liquefied gas electrolyte with the THF additive. Reproduced with permission [46]. Copyright 2019, Elsevier. (d) Schematic diagram of Li growth and SEI formation mechanism in the FSA electrolyte. Reproduced with permission [48]. Copyright 2020, Royal Society of Chemistry. (e) Schematics of three types of PLs and traditional liquids. Adapted with permission [96]. Copyright 2025, American Chemical Society. (f) Schematics of the effect of crown ether additives on the decomposition process of solvents. Reproduced with permission [97]. Copyright 2020, Wiley‐VCH.

As discussed before, a key disadvantage of DME is its strong coordination with Li^+^, which causes excessive solvent participation in the solvation sheath. This necessitates the use of HCEs or LHCEs with diluents to form a stable anion‐driven SEI [15, 20, 36]. Alternatively, WSEs employ solvents with low DN and dielectric constant *ε* to limit Li^+^‐solvent interactions, thereby reducing solvent involvement in solvation and promoting anion‐derived SEI formation. Through tuning the structure of DME, various weakly solvating solvents have been developed (Figure 3a).

For example, LiFSI in 1,2‐diethoxyethane (1,2‐DEE, *ε* = 3.9) was reported as a WSE. Compared to DME, the terminal alkyl groups of 1,2‐DEE increase the steric hindrance, weakening the interaction between Li^+^ and solvent molecules [39, 40]. However, the better cycling performance was achieved with a high concentration of 4 M instead of 1 M, indicating that simply increasing steric hindrance makes 1,2‐DEE only a moderately weakly solvating solvent, and a high concentration is necessary to achieve enhanced cycling stability. Another ether solvent of interest is DMM, which, despite lacking bulky terminal groups, exhibits a unique molecular conformation due to the anomeric effect. This effect favors a gauche‐gauche conformation that is unfavorable for forming a four‐membered ring, thereby preventing strong chelation with Li^+^ [24]. As a result, DMM demonstrates weak solvation characteristics that make it a promising candidate for weakly solvating electrolytes despite its simpler structure. Approximately 99% Li CE of 1 M LiFSI in DMM (0.5 mA/cm^2^ with 0.5 mAh/cm^2^) was reported by Chen's group, and 1.5 M LiFSI in DMM (0.5 mA/cm^2^ with 0.5 mAh/cm^2^) was reported by Yamada's group [24, 42]. However, the low boiling point of DMM (42°C) makes it highly volatile and difficult to handle at room temperature.

In contrast, solvents such as methyl *tert*‐butyl ether (MTBE, melting/boiling −109°C to 55°C, ɛ = 2.6) [45], diethyl ether (DEE, −117°C to 35°C, ɛ = 4.33) [38], cyclopentyl methyl ether (CPME, −140°C to 106°C, ɛ = 4.7) [45], and dibutyl ether (DBE, −98°C to 142°C, ɛ = 3.045) [82] contain only one oxygen atom, lack chelation ability, and have lower dielectric constants, making them ideal weakly solvating solvents for WSEs that improve lithium metal anode stability. For example, 1 M LiFSI in MTBE/toluene electrolytes achieved a high Li CE of 99.70% [44]. The reduced interaction between Li^+^ and ether leads to a more anion‐derived SEI layer. Energy dispersive X‐ray spectroscopy (EDX) indicated that the SEI layer is oxygen‐rich. From an industry standpoint, MTBE is low‐cost and scalable, with both solvents being fluorine‐free. However, their mixture with toluene further lowers electrolyte ionic conductivity. Additionally, long‐term cycling performance data for these electrolytes remain unavailable. CE was only obtained from the modified Aurbach method, which brings into question the extent of activation needed and its consequences on improving the CE. These solvents’ class possess a wide liquid range. Their weak interaction with Li^+^ facilitates easier desolvation. The combination of a broad liquid range and faster desolvation kinetics makes them interesting for low‐temperature battery applications (below 0°C) [83].

Good low‐temperature performance (CE of 98.4%, 0.5 mA/cm^2^, −60°C, modified Aurbach method) was reported by Chen and Liu et al. in 1 M LiFSI in DEE [38]. SEM images showed dense lithium plating from 23°C down to −60°C. A systematic study comparing 1 M LiFSI in DEE and 1 M LiFSI in DME/DOL revealed that the solvation structure plays a crucial role in maintaining good battery performance at low temperatures, whereas strong bonding between Li^+^ and DME makes desolvation more difficult at low temperatures, highlighting the advantage of weakly solvating solvents under these conditions. Additionally, 1 M LiFSI in dipropyl ether (DPE) and DBE also exhibited improved performance at −40°C, with Li CE of 97.3% and 98.2%, respectively. However, it is important to note that the boiling point of DEE is only 35°C, making it highly volatile and challenging to handle at room temperature. The higher‐boiling DBE was investigated as a more viable solvent for ultralow‐temperature LMBs due to weak Li^+^–solvent interactions and strong Li^+^‐anion interactions, which promote ion pairing in solution and facilitate easier ion desolvation at low temperatures. LiFSI (2 M) in DBE had a Li CE of 99.0% at room temperature and 98.0% at −50°C (modified Aurbach method at 0.5 mA/cm^2^) [41]. The electrolyte performed well at both low and high temperatures due to its optimized solvation structure and good wettability. On the other hand, because of DBE's weak coordinating ability, common salts like LiPF_6_ and LiBF_4_ are not soluble in it. Additionally, 2 M LiTFSI in DBE exhibits a lower Li CE of only 96.5%. Cyclic weakly coordinating solvents were also investigated. In particular, CPME has a wider liquid temperature window (−140°C to 106°C) attributed to the cyclopentyl group, which increases steric hindrance around the oxygen atom, making CPME a weakly solvating. Good performance in CPME was achieved in low and high salt concentrations. For example, 99.0% was obtained in LiFSI‐10CPME (0.96 g/mL, 0.81 M) vs. 99.4% (0.5 mA/cm^2^ with 0.5 mAh/cm^2^, modified Aurbach method) in LiFSI‐2CPME (molar ratio, 1.23 g/mL, ∼3.17 M) [45]. However, it is worth noting that significant fluctuations were observed during cycling in LiFSI‐10CPME (0.81 M). Other weakly solvating solvents were investigated, including 1,4‐dioxane [22], 1,2‐diethoxypropane (DEP) [84], 1,3‐dimethoxypropane (DMP) [85], tetrahydropyran (THP) [86], 1,3‐dioxane and ethylene glycol dibutyl ether (EGDBE) [87], and 1‐(2‐methoxyethoxy)butane (M4C) [88].

The concept of weakly solvating electrolytes was extended recently to fluorinated ethers. These solvents are designed to combine the benefits of weak Li^+^ coordination with enhanced chemical and electrochemical stability, addressing some limitations of conventional ether solvents. Cui and Bao's groups demonstrated that WSEs can be produced using partially fluorinated 1,2‐DEE [37, 43]. To enhance oxidative stability and weaken solvation ability, longer alkyl chains and fluorine groups were introduced to DME, resulting in the synthesis of the fluorinated 1,4‐dimethoxybutane (FDMB) solvent. FDMB exhibits an oxidative stability greater than 6 V and achieves a Li CE of 99.52% (modified Aurbach method at 0.5 mA/cm^2^). Additionally, a Li|NMC532 coin cell with this electrolyte maintained 90% capacity retention over 400 cycles at C/3 (1C = 2.9 mA/cm^2^), while anode‐free Cu|NMC 811 pouch cell with energy density of approximately 325 Wh kg^−1^ retained 80% of its capacity after 100 cycles at C/5 charge and C/3 discharge (1C = 3.9 mA/cm^2^) [37]. However, the poor solvation ability of 1 M LiFSI in FDMB leads to low ionic conductivity of 3.5 mS/cm and high overpotential [37, 43]. To balance solvation ability and ion transport, partially fluorinated ─CHF_2_ or ─CF_3_ groups were introduced into 1,2‐DEE (Figure 3b). These fluorinated electrolytes reduce the donor strength of the ether solvents, thereby lowering their solvating ability. This shift in solvation structure favors ion pairing, suppresses Li^+^‐solvent coordination, and increases anion involvement. As a result, the ionic conductivity of 1.2 M LiFSI in F3DEE to F6DEE ranges from 4.48 to 6.18 mS/cm. Lithium CE of 99.3% to 99.5% were achieved (modified Aurbach method cycling, 0.5 mA/cm^2^ with 1 mAh/cm^2^). LiFSI (1.2 M) in F5DEE achieved a Li high CE of 99.90% (0.5 mA/cm^2^ with 1 mAh/cm^2^), with CE fluctuations within ±0.1%. However, this high CE was only observed between 100 and 600 cycles, demonstrating that practically achieving 99.9% CE remains thus far not possible. These partially fluorinated electrolytes enabled approximately 270 cycles in Li|NMC811 cells at 0.1 C charge and 0.3 C discharge (1C = 200 mA/g), and over 140 cycles in Cu|LFP pouch cells at 0.5 C charge and 2 C discharge (1C = 155 mA/g). X‐ray photoelectron spectroscopy (XPS) analysis revealed that, in addition to increased LiF content, more Li_2_O was detected in the SEI compared to that generated in 1,2‐DEE solutions. This suggests that Li_2_O may be contributing to improved cycling performance [89]. Using a similar strategy, other weakly fluorinated coordinated solvents have been developed, including fluorinated 1‐ethoxy‐2‐methoxyethane (EME, FxEME, x = 1–3) [90, 91] and fluorinated 1‐methoxy‐3‐ethoxypropane (FxEMP, x = 1–3) [92].

Other approaches sought to increase the ionic conductivity by increasing the solvation strength of the fluorinated solvents. Recently, Bis(2‐fluoroethyl)ether (BFE) was investigated [93]. Although the fluorinated groups reduce the interaction between Li^+^ and oxygen atom (Li–O), in BFE, Li^+^ strongly interacts with fluorine atoms (Li–F). Therefore, Li^+^ and BFE coordination is maximized through strong tridentate Li–F and Li–O interactions [43]. This resulted in high ionic conductivity (∼8 mS/cm at 30°C, 0.95–15 mS/cm from −60°C to 70°C) and enhanced stability with lithium metal and high‐voltage cathodes. A Li CE of 99.75% was achieved with 2 M LiFSI in BFE (0.5 mA/cm^2^ with 1 mAh/cm^2^, modified Aurbach method). Raman spectroscopy and MD simulations show that SSIPs and CIPs dominate the solvation structures. This example is particularly interesting as it demonstrates the possibility to achieve enhanced solvation in fluorinated ether solvents.

Despite their promising electrochemical properties, fluorinated ethers face notable challenges. Although many can be synthesized via relatively simple one‐step processes, scalability and cost remain significant barriers. Moreover, many fluorinated ethers are not environmentally friendly and suffer from limited commercial availability, restricting their practical application in large‐scale battery manufacturing. From an electrochemical perspective, poor solvation capability is prevalent in these solvents, which leads to ion clustering, poor ion mobility, and low salt solubility, all contributing to decreased ionic conductivity, which adversely compromises the rate capability of LMBs. Moving forward, it is crucial to strike a careful balance between solvation strength and ionic conductivity. Optimizing this compromise is essential to enhance ion transport, maintain electrolyte stability, and ultimately improve the overall performance and longevity of LMBs.

#### 2.2.3 Liquefied Gas Electrolytes

In 2017, Meng's group developed fluoromethane (FM)‐based liquefied gas electrolytes (LGEs) to exploit their low viscosity and low melting point. Impressive low‐temperature operation down to −60°C and good stability with both a Li metal anode and a 4 V class cathode [19]. However, the low solubility of LiTFSI in FM limits the cell performance (Figure 3c), so the liquefied gas is typically blended with other solvents. For example, adding THF or AN as a cosolvent increased the salt's solubility and ionic conductivity [46, 47]. Specifically, a 0.5 M LiTFSI and 0.5 M THF solution in FM, where THF is fully coordinated to Li^+^, exhibits a high ionic conductivity of 2.8 mS/cm at −60°C. This value is significantly higher than the 1.1 mS/cm observed for 0.2 M LiTFSI in FM. Additionally, the 0.3 M LiTFSI and 0.3 M THF in FM electrolyte achieved a Li CE of 99.6% over 500 cycles (0.5 mA/cm^2^ with 0.5 mAh/cm^2^), with an impressive average CE of 99.9% maintained from the 100th to the 500th cycle at room temperature. It is important to note that approximately ±1% CE fluctuation was observed, occasionally causing CE values to exceed 100%. This phenomenon is likely due to the recovery of isolated lithium from previous cycles. On the other hand, CE values below 99% may indicate instability of the SEI layer or the occurrence of side reactions. In addition, the electrolyte achieved a Li CE of 98.4% (0.5 mA/cm^2^ to 1 mAh/cm^2^) at −60°C. AN cosolvent further improves the solubility and conductivity of LiTFSI. The 1.2 M LiTFSI and 1 M AN/FM solutions exhibit a high Li^+^ transference number (∼0.7) and fast ion transport (>4 mS/cm) across a wide temperature range from −78°C to 75°C. Interestingly, dense Li deposition occurred even at −60°C interestingly despite the presence of AN solutions, which is typically detrimental for Li metal cycling. The electrolyte maintains a Li CE of 99.4% when 3 mAh/cm^2^ was cycled at 3 mA/cm^2^ for 200 cycles at room temperature. A Li CE ranging from 96.4% at −60°C to 99.4% at 55°C was achieved under the same conditions, demonstrating stable cycling performance in a wide temperature range. Non‐fluorinated liquefied gas electrolytes were also investigated and shown to have a beneficial effect. For example, 1 M LiFSI in Me_2_O–1,1,1,2‐tetrafluoroethane (TFE)–pentafluoroethane(PFE) had high ionic conductivity in a wide temperature range (>3 mS/cm from −78°C to 80°C), a Li CE of 99.0% over 200 cycles (3 mA/cm^2^ with 3 mAh/cm^2^) at room temperature and stable cycling from −60°C to 55°C [94]. It is important to note that LGEs require high pressure to maintain their gas components in the liquefied phase. For example, the vapor pressure of FM is 3.41 MPa, which necessitates additional containment and reduces the specific energy density in practical applications. Recently, the concept of coulombic condensation was introduced, which entails a saturated LiFSI‐Me_2_O electrolyte (mol:mol = 1:2.36) that remains liquid under ambient conditions, thus eliminating the need to apply pressure [95]. Similar to other HCEs, stable lithium cycling was achieved due to the LiF‐rich SEI formed from the anion‐rich solvation structure. Interestingly, owing to the to low viscosity of Me_2_O, this saturated LiFSI–Me_2_O electrolyte exhibits lower viscosity (27.74 mPa/S) and improved ionic conductivity (0.17 to 3.54 mS/cm from −60°C to 0°C) below 0°C. These unique properties enable stable cycling across the temperature range from −40°C to 50°C in Li|Cu half cells (Modified Aurbach method, 0.5 mA/cm^2^, CE = 98.4% for −40°C and −20°C, 99.0% at 50°C) and in Li|SPAN full cells at current densities up to 6 mA/cm^2^. It should be noted that the coulombic condensation approach still relies on gas‐condensation steps to produce these solutions. Even so, it provides a promising route to formulate other liquefied‐gas electrolytes and assess their performance in unpressurized battery cells.

#### 2.2.4 Other Novel Electrolytes

Electrolyte engineering has been employed to address the limitations of traditional ether‐ and carbonate‐based electrolytes, which lack cathodic stability above 4 V and are incompatible with LMA, resulting in low CE. Consequently, novel electrolytes with alternative salts and solvents are needed to meet the requirements of LMBs. For example, in 2020, inspired by the decomposition of the FSI anion in HCEs and LHCEs on the lithium metal surface, which generates a LiF‐rich SEI layer that supports high‐performance cycling in LMA, Shao‐Horn, Johnson and Li et al. reported *N*,*N*‐dimethylsulfamoyl fluoride (FSA) as a solvent for battery electrolytes (Figure 3d) [48]. This solvent offers sufficient solubility for lithium salts and a wide electrochemical window. Additionally, DFT calculations show that the LUMO energy of FSA is similar to that of FEC, making FSA prone to decomposition, thus facilitating LiF formation on the lithium metal anode surface. By adding 0.2 m LiPF_6_, 2.5 m LiFSI and 0.2 m LiPF_6_ in FSA electrolyte, the cathodic stability exceeded 4.5 V and prevented Al corrosion, enabling a Li CE of 99.03% over 400 cycles (0.5 mA/cm^2^ with 0.5 mAh/cm^2^). Furthermore, a Li|NMC622 cell with a cut‐off of 4.3 V exhibited a capacity retention of 89% after 200 cycles. Consequently, a similar strategy of 1 m LiFSI in *N*,*N*‐dimethyltrifluoromethane‐sulfonamide (DMTMSA) was investigated [49]. To be noticed, this electrolyte is WSE due to low polarity, and it creates more anion‐derived SEI. The electrolyte enables stable cycling of NMC811 cathodes under 4.7 V with a high specific capacity of 231 mAh/g at 0.1 C and a Li CE of 99.65% over 100 cycles, by suppressing cathode particle intergranular stress corrosion cracking (SCC), partially due to decreased transition metal ion solubility in the sulfonamide‐based electrolyte. The two solvents above fully leverage the advantages of the FSI anion, offering new options for designing electrolytes for high‐performance LMA. Building on FSA and DMTMSA, additional sulfonyl solvents have been developed with modifications to the F or N─C groups, including nonafluoro‐*N*,*N*‐dimethylbutane sulfonamide (NFS) [98], 1‐((trifluoromethyl)sulfonyl)piperidine (TFSPP) [99], *N*‐morpholine‐trifluoromethanesulfonamide (TFSMP), *N*‐pyrrolidine‐trifluoromethanesulfonamide (TFSPY) [100], and 1‐azetidine trifluoromethanesulfonamide (AzTFSA) [101]. Although sulfonamide solvents demonstrate notable advantages such as high oxidative stability and the ability to form robust solid electrolyte interphases that suppress lithium dendrite growth, their practical application in lithium metal batteries is questionable. Compared to traditional carbonate or ether‐based solvents, sulfonamides also tend to be more expensive and synthetically complex, which may limit scalability. Therefore, while sulfonamide solvents represent a promising direction for safer and more stable lithium metal batteries, further optimization and comprehensive testing under realistic conditions are essential before they can be considered viable for commercial use.

In addition, other solvents classes have been explored for LMBs, such as silanes [102, 103, 104], nitrile [105], and phosphate [106, 107], each offering unique properties that may enhance electrolyte performance. Silane‐based solvents have shown potential for improving interfacial stability, nitriles provide high oxidative stability and wide electrochemical windows, and phosphate solvents contribute to enhanced thermal stability and flame retardancy [108]. Despite these advances, significant challenges remain, and the new electrolytes have yet to match the overall benchmark performance, especially in terms of cycling stability and coulombic efficiency, demonstrated by traditional carbonate or ether‐based electrolytes. This performance gap highlights the need for further research to tailor solvent properties and electrolyte formulations to fully realize the potential of alternative solvents in LMB applications.

#### 2.2.5 Ionic and Porous Liquid Electrolytes

Ionic liquids (ILs) are salts that remain liquid at room temperature. As liquid salts, ILs typically exhibit significant ionic conductivity, are non‐flammable, have good thermal stability, and possess wide electrochemical windows, making them promising candidates for use as electrolytes [109, 110]. IL electrolytes have been extensively studied for a long time. ILs help stabilize the SEI layers in LMA. For example, *N*‐methyl‐*N*‐butylpyrrolidinium bis(trifluoromethylsulfonyl)imide (Py_14_TFSI) has been shown to promote the formation of a protective SEI layer that prevents the polysulfide shuttle in Li‐S cells [111]. The growth of Li dendrites and the corrosion of the Li metal anode in LMBs can be effectively suppressed by surface passivation using the optimized hybrid *N*‐propyl‐*N*‐methylpyrrolidinium bis(trifluoromethanesulfonyl)amide (Py_13_TFSI) IL electrolyte [112]. In addition, IL 1,1‐diethylpyrrolidinium bis(fluorosulfonyl)imide (Pyr2(2)FSI) was used to modify the Li^+^ solvation structure of 1.5 M LiFSI in DME to improve the ionic conductivity and lower desolvation barrier, enabling anion‐derived SEI and stable cycling [113]. ILs incorporating anodically stable anions beyond conventional chemistries have been explored. In 2018, room‐temperature ionic liquids (RTILs) based on the *closo*‐carborane anion were reported; these were enabled by introducing structural disorder into the organic cation, which suppressed crystallization and facilitated RTIL formation. Notably, this class remains the only known IL family compatible with both Li and Mg metals [114].

Despite significant progress, the use of IL electrolytes in LMBs remains challenging. Key challenge includes the high cost and complex synthesis, limited understanding of how the structure and dynamics in IL electrolytes relate to their electrochemical and transport behavior, high viscosities leading to limited room‐temperature conductivities, in addition to environmental impact concerns.

Beyond conventional electrolytes, porous liquids (PLs) are interesting due to their unique properties. PLs are classified into three types based on their structure and pore characteristics (Figure 3e) [96]. Type I porous liquids are neat liquid materials composed of individual molecules that possess permanent, rigid internal cavities, such as porous organic cage‐structure molecules and certain ILs with cationic or anionic cages. Type II porous liquids involve porous molecules dissolved in a solvent, where the solvent molecules cannot enter the pores. Type III porous liquids comprise porous solids, such as metal‐organic frameworks (MOFs) and crown ethers, dispersed in solvents to form stable colloids. The porous molecules in Types II and III can be the same; the only difference is whether the molecules are dissolved or dispersed in the solvent [115]. Although studies on the compatibility of PLs with battery electrodes are still limited, there are positive suggestions that their components, such as crown ethers, MOFs, and ILs, can improve compatibility with LMA and can enhance the stability and cycling performance of LMBs. For example, in 2021, Ma's group demonstrated that 15‐crown‐5 ether (15C5) molecules, used as an additive in LiPF_6_ dissolved in EC/DMC electrolyte, form stable complexes with Li^+^ that regulate the solvation structure and promote uniform lithium deposition (Figure 3f) [97]. The interaction also facilitates the formation of a dense SEI, improving cycling performance and preventing dendrite growth. PLs may offer unique advantages in battery electrolyte applications, including improved ion mobility, increased surface area for ion interaction, enhanced ionic selectivity, improved thermal stability, and suppression of Li dendrite formation [96]. By controlling the pore size of PLs, metal ions such as Li^+^, Na^+^, and Mg^2+^ can selectively transport through the porous structure while blocking solvents in the electrolyte. This could be a promising approach if the goal is to eliminate the solvent molecules from the Li^+^ solvation shell prior to reductive electron transfer. This selective ion transport can support higher ionic conductivity by creating fast diffusion pathways. For example, crown ether‐based type II PLs can improve the ionic conductivity of 1M LiTFSI in PC/EC [116]. Specifically, electrochemical measurements and simulations reveal that the 12‐crown‐4 ether (12C4) pore size matches the Li^+^, facilitating the dissociation of Li‐TFSI salt, reducing ion–solvent interactions, and promoting the formation of stable complexes within the cavity. In 12C4 solutions (Li^+^/12C4 = 1), the ionic conductivity increases from 5.55 to 6.33 mS/cm, and the Li^+^ diffusion coefficient *D*_Li_^+^ increases slightly from 1.72 × 10^−10^ to 2.12 × 10^−10^ m^2^/s. However, it is important to note that weakening the Li^+^‐anion interactions is not necessarily always desired, as it results in a vehicle‐type transport mechanism (*D*_Li_^+^/*D*_12C4_ ≈ 1, PFG NMR), which negatively impacts the transference number of Li^+^ and also prevents the formation of inorganic rich SEI. Additionally, high desolvation barriers can lead to high overpotentials and low rate capabilities.

Compared with conventional liquid electrolytes that rely on flammable organic solvents, porous liquid electrolytes (PLEs) may offer safety advantages, especially when ionic liquids are used or when solvent content is minimized, as in the porous‐solid‐based systems. Nevertheless, further work is needed to determine their true compatibility with metal anodes, define and design optimal porosities, and minimize cation binding energies and develop scalable synthesis routes. From a fundamental perspective, a clearer understanding of ion‐transport pathways within the porous architecture and of the interactions between PLE components and lithium metal is essential.

#### 2.2.6 Electrolyte Additives

Electrolyte additives are small amounts of chemical compounds added to battery electrolytes to enhance performance, stability, and safety. They can improve the formation and stability of the SEI layer, suppress unwanted side reactions, enhance ionic conductivity, and increase thermal and electrochemical stability. In 1996, hydrofluoric acid (HF) was reported as an additive to suppress the generation of Li dendrite‐formation in LMA through generating LiF/Li_2_O‐rich SEI [117]. Since then, significant progress has been made in the development of electrolyte additives for graphite/silicon‐based LIBs. In LIBs, solvent additives vinylene carbonate (VC) and FEC are two of the most common additives for modifying SEI formation in Li‐ion batteries, significantly improving cell performance [118, 119, 120, 121]. Specifically, both additives form polymer species during reduction and FEC is also known to produce LiF in the SEI. In the early development of LMBs, especially those using carbonate‐based solvents, VC and FEC were investigated as additives to enhance battery performance [80, 81, 122, 123]. FEC was also used in ether‐based electrolytes. For example, LiTFSI in DOL/DME with 10% FEC could improve the CE of lithium metal anodes at −60°C, where the decomposition of FEC at low temperature produced LiF and Li_2_CO_3_ [124]. However, FEC is used more commonly in carbonate‐type systems because it was effective at addressing the main interfacial problems of carbonate electrolytes [125]. In general, fluorine‐containing carbonate additives can come with trade‐offs, such as increased interfacial resistance [126] and therefore less commonly used than fluoroethers.

In addition, salt additives have also been extensively studied. For example, LiNO_3_ is the most widely studied salt additive for LMBs. Initially, LiNO_3_ was introduced as an additive in lithium–sulfur (Li‐S) batteries to enhance cell performance by suppressing the shuttle effect [127, 128, 129]. NO_3_^−^ has a high cathodic decomposition potential of ∼1.7 V versus Li/Li^+^. Upon reduction, it forms insoluble Li_x_NO_y_ /Li_3_N/Li_2_O species that integrate into the SEI layer, and this SEI modification promotes uniform lithium deposition [128, 130, 131, 132]. However, adding additives also introduces certain drawbacks. The decomposition of VC and FEC can generate gases such as O_2_, H_2_, CO_2_, and C_2_H_4_ [133, 134], which raise serious safety concerns. Additionally, LiNO_3_ has poor solubility in carbonate solvents, requiring alternative strategies for its effective use [135, 136]. The inclusion of additives also increases the cost and complexity of battery manufacturing. In conclusion, electrolyte additives can offer significant advantages, including improved cycle performance through stabilizing the SEI layers, enhanced safety by mitigating dendrite formation and thermal risks, increased ionic conductivity, better capacity retention, and reliable performance across a wide temperature range. However, they also have drawbacks such as added cost, potential degradation and outgassing over time, and possible side reactions or compatibility issues with other battery components [137, 138, 139].

### 2.3 SEI Layer

#### 2.3.1 Structure Models of SEI

**FIGURE 4:**
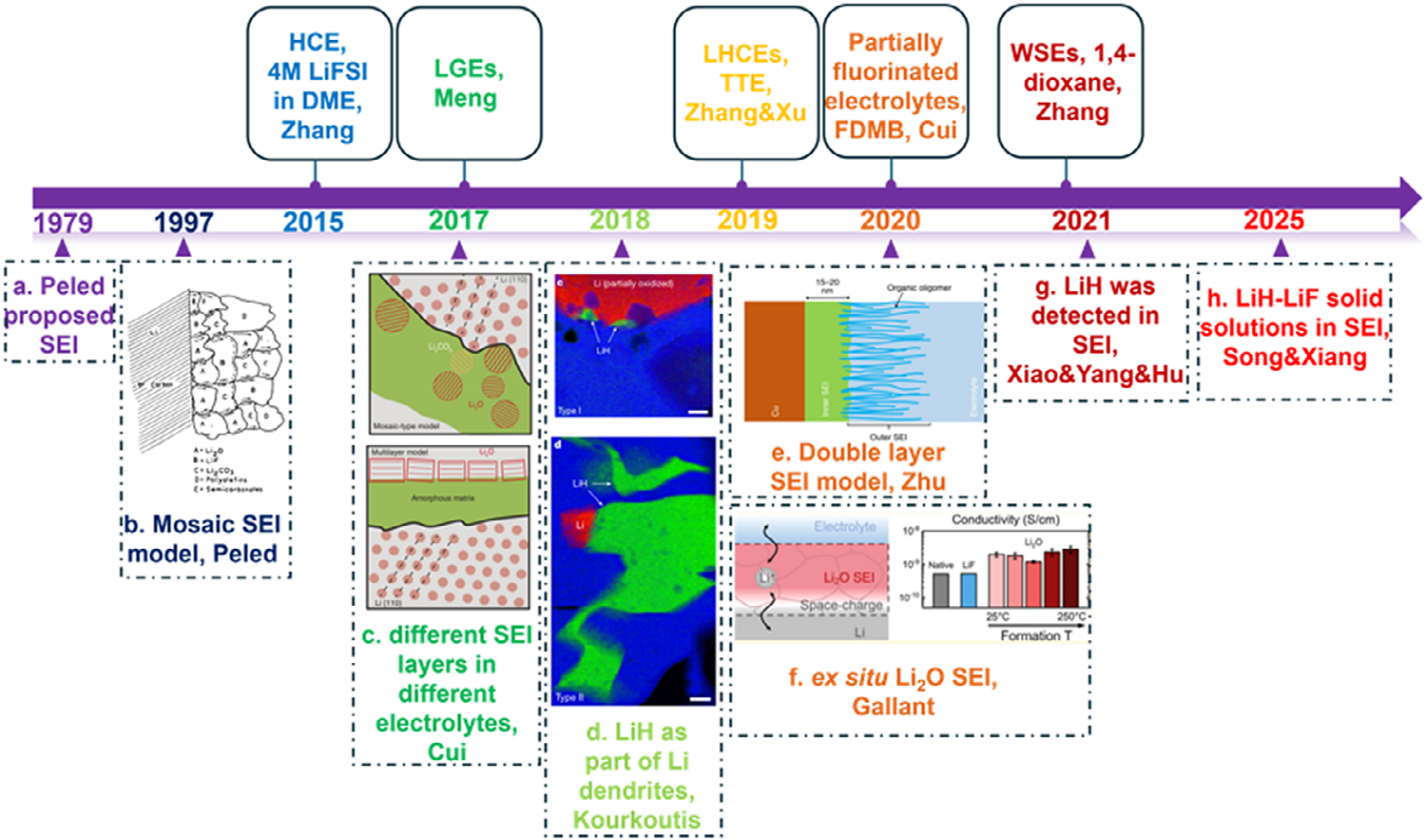
The investigation of SEI models and development of electrolyte design timeline. (a) The concept of SEI layer [140]. (b) Mosaic SEI model was proposed. Reproduced with permission [141]. Copyright 1997, IOP Publishing. (c) Two different SEI structures were observed in different electrolytes by cryo‐TEM. Reproduced with permission [143]. Copyright 2017, AAAS. (d) LiH was observed as part of Li dendrites by cryo‐TEM. Reproduced with permission [157]. Copyright 2018, Nature Publishing Group. (e) Double layer SEI model. Reproduced with permission [145]. Copyright 2020, Nature Publishing Group. (f) Ex situ Li_2_O SEI layer investigation. Reproduced with permission [89]. Copyright 2020, America Chemical Society. (g) LiH was detected in SEI by XRD and PDF [160]. (h) LiH–LiF solid solutions were detected in the SEI layer [162].

The SEI is a crucial passivation layer that protects the electrode from continuous electrolyte decomposition while allowing lithium‐ion transport. The SEI was first proposed by Peled in 1979 to describe the passivated layer formed between the metal and the liquid electrolyte [140]. Since then, extensive research has been conducted on the SEI. However, the formation mechanisms and structure of the SEI layer are still not fully understood. Several models of SEI structure have been proposed. In 1997, Peled proposed the mosaic model, which describes the SEI as consisting of an inorganic‐rich layer and an organic‐rich layer. This model assumes that each component forms a distinct pure microphase, and the SEI is a mosaic assembly of these different microphases [141]. The main components include LiF, Li_2_O, Li_2_CO_3_, polylefins, and semicabonates. Similarly, in 2000, Aurbach described the SEI layer on the lithium metal surface as consisting of an inner inorganic layer and an outer organic layer. The elastic organic polymer in the outer layer is important because it helps maintain the integrity of the film during the volume changes of anode throughout cycling [142]. Even though the mosaic model became most accepted based on XPS analyses, cryo‐transmission electron microscopy (cryo‐TEM) studies have deviated from this model. For example, in 2017, based on cryo‐TEM measurements, Cui's group observed that the SEI formed in EC–DEC electrolytes was a classic mosaic structure [143]. On the other hand, the SEI formed in a FEC‐based electrolyte was a multilayer structure, where the inner layer was amorphous polymer matrix, and the outer layer was mainly a crystalline Li_2_O layer. Surprisingly, although XPS analysis shows that LiF is abundant in the SEI, it was not detected in the SEI layer by cryo‐TEM. This discrepancy likely arises because TEM examines a very limited area and primarily identifies crystalline phases, while parts of the SEI may be amorphous. Several studies support this interpretation. For example, a 1 M LiFSI‐DME‐TFEO electrolyte forms an amorphous, inorganic‐rich, single‐layer SEI that is uniform both laterally and in depth [36]. Additionally, in 2020, utilizing cryo‐TEM and electron energy loss spectroscopy (EELS), LiF was absent from the compact SEI in 1 M LiPF_6_ in EC/DEC+10% FEC electrolyte, but it can deposit on the current collector and SEI surface to aid Li plating uniformity to some degree [144]. In 2020, Zhu and co‐workers proposed a double‐layer SEI model, which demonstrated that an inner inorganic and compact layer and an outer permeable organic oligomer layer through using in‐situ liquid secondary ion mass spectrometry [145]. Looking back, it is evident that the structural model of the SEI has evolved significantly over time (Figure 4). These improvements have provided deeper insights into the SEI's complex composition, morphology, and formation mechanisms. However, despite these advances, the exact structure and properties of the SEI remain not fully understood. This is largely because the SEI is highly dynamic and its formation strongly depends on the specific electrolyte chemistry, including salt type, solvent composition, additives, and operating conditions. As a result, the variability of SEI makes it challenging to establish a universal structural model. Ongoing research continues to uncover new aspects of SEI behavior, highlighting the need for tailored electrolyte designs to optimize SEI properties for different battery systems.

#### 2.3.2 Mechanical Properties of SEI Components

Considering the large volume change during Li plating and stripping in LMA, the mechanical properties of the SEI are crucial. Cracking of the SEI allows the electrolyte to contact and react with fresh Li, triggering further parasitic reactions that promote continued SEI growth and thickening while consuming the electrolyte. As a result, cell polarization increases, and severe degradation can ultimately lead to cell failure.

Atomic force microscopy (AFM) can be a powerful tool for probing the nanoscale mechanical properties of SEI layers because it combines topographical imaging with local force measurements, enabling both elastic‐property characterization and high spatial resolution. The effectiveness of AFM, together with XPS and atomistic calculations, was shown through studies of composition–structure‐dependent elasticity of the SEI formed on highly oriented pyrolytic graphite (HOPG) [146]. XPS results showed that the outer SEI is enriched in organic and polymeric species, whereas repeated cycling increases the fraction of inorganic components such as Li_2_CO_3_ and LiF, which are associated with greater stiffness. AFM measurements showed that the Young's modulus of the SEI on HOPG was mainly in the range of 0.2–4.5 GPa. After the outer SEI was removed by AFM scratching, the measured modulus increased, indicating that the inner SEI layer is mechanically stiffer than the outer layer. Atomistic simulations further revealed that the Young's modulus of individual SEI components increases in the order polyethylene (PEO) < LiEC < LiMC < LiEDC < Li_2_CO_3_ < LiF. This trend confirms, as expected, that inorganic SEI species such as Li_2_CO_3_ and LiF are significantly stiffer than organic species like LiEC, LiMC, and Li_2_EDC, as well as polymeric components such as PEO.

To mitigate issues related to substrate interferences using traditional AFM, a more rigorous analysis of SEI mechanics was shown using a two‐step AFM nanoindentation method that corrects for substrate effects, which can otherwise overestimate the SEI Young's modulus by up to 200% [147]. Using this approach, the authors extracted both the Young's modulus and elastic strain limit of the SEI, and proposed the maximum elastic deformation energy as a more meaningful descriptor of SEI mechanical stability. This parameter showed a strong correlation with CE and cycling stability in both Li and K metal systems, indicating that SEI stability depends not only on stiffness but also on the ability to elastically accommodate deformation before failure. These findings suggest that an ideal SEI should combine sufficient stiffness with high strain tolerance to withstand repeated electrode volume changes.

Overall, elucidating the mechanical properties of the SEI is of fundamental importance, as they dictate the local interphase response to the substantial volume fluctuations associated with repeated Li plating and stripping. A mechanistic understanding of SEI stiffness, elasticity, and failure behavior is therefore indispensable for clarifying how the interphase maintains passivation, accommodates strain, and suppresses continuous side reactions during cycling.

#### 2.3.3 Organic Components in the SEI

Organic components are typically detected in the outer layer of the SEI and are generally formed by decomposition of solvent molecules. In carbonate‐based electrolytes, these species commonly include lithium alkyl carbonates, whereas in ether‐based electrolytes, lithium alkoxides (ROLi) are more often observed [142]. In addition, oligomeric and polymeric species have also been identified in the SEI [148]. For example, polydioxolane has been reported in the SEI formed in DOL‐based electrolytes [142]. The elastic properties of polydioxolane help preserve film integrity and accommodate anode volume changes during cycling. More generally, organic SEI components are considered mechanically softer and more deformable than inorganic phases, which may help buffer interfacial stress and suppress crack formation during repeated plating and stripping. Very recent work by Lopez's group further supports the importance of organic SEI components [149]. Using LiPF_6_ in FEC and a spin‐trapping/electron paramagnetic resonance (EPR) approach with 2‐methyl‐2‐nitrosopropane (MNP), they directly identified radical intermediates during FEC reduction and showed that the reaction proceeds mainly through a ring‐opening pathway. More importantly, when MNP was used to suppress the formation of organic polymeric species derived from FEC, the resulting interphase contained more LiF but became less stable and more resistive, with higher interfacial resistance and evidence of accelerated electrolyte decomposition. These results indicate that a stable interphase requires not only inorganic species such as LiF, but also sufficient organic SEI components.

#### 2.3.4 LiF and Li_2_O in the SEI

Although the SEI consists of both organic and inorganic components, only a few reports have shown that organic‐rich SEIs enhance LMA performance. Inorganic components are widely recognized as crucial for forming a stable SEI due to their relatively high ionic diffusion rate, low solubility, high surface energy, and strong mechanical properties. Among these, LiF stands out for providing a dense, chemically stable, and electronically insulating layer that effectively suppresses further electrolyte decomposition. During the substantial volume changes associated with lithium plating and stripping, LiF is believed to maintain SEI structural integrity by minimizing cracks and preventing continuous side reactions between lithium metal and the electrolyte. As a result, LiF is widely regarded as essential for forming a stable, uniform SEI that helps suppress lithium dendrite growth [150]. A popular strategy to improve LMBs' performance involves tuning the solvation structure to favor the formation of anion‐derived SEI rich in LiF. Such LiF‐rich SEI primarily forms through the decomposition of fluorine‐containing anions as discussed earlier, which enhances mechanical robustness and ionic conductivity, ultimately improving cycling stability and Coulombic efficiency. Interestingly, Gallant's group demonstrated through *ex*‐situ and *in*‐situ studies that LiF is not intrinsically protective and can in fact degrade under cycling stress. This work suggests that other SEI components are contributing to SEI robustness, and underscores the importance of addressing SEI's integrity during cycling [151].

Despite widespread reports of abundant LiF in the SEI and many high‐performance LMB designs focusing on increasing fluorine content, the exact role and importance of LiF remain debated. For example, XPS pretreatment can inadvertently alter the analytical results, with LiF artifacts generated from LiPF_6_, LiFSI, and LiTFSI during argon ion sputtering. Evidence also suggests that in 1 M LiPF_6_ in EC/EDC electrolyte, LiF primarily influences lithium nucleation in early deposition stages, but its effect diminishes as deposition capacity grows [152].

Beyond LiF, Li_2_O is another important SEI component that only recently has received attention. Li_2_O often coexists with LiF in SEIs formed from high‐performance electrolytes [43, 44]. In 2020, Gallant's group demonstrated that a single Li_2_O SEI grown on lithium metal via a metal–gas reaction exhibits a higher ionic conductivity of 10^−9^ S/cm and a lower diffusion energy barrier of 0.152eV, compared to LiF SEI (5.2 × 10^−10^ S/cm) produced using the same method [89, 153]. Other studies reported that the energy barrier of LiF is 0.729 eV with ionic conductivity of approximately 10^−13^ to 10^−14^ S/cm [153, 154]. These findings highlight potential advantages of Li_2_O as an SEI component in promoting more homogeneous Li^+^ transport.

In fact, titration analysis of SEI layers from various electrolytes revealed a significant amount of Li_2_O content, with CE strongly correlated to Li_2_O amount depending on the electrolyte [155]. For example, 1 M LiTFSI in DOL/DME electrolyte + 3 wt% LiNO_3_ achieved a CE of 99.0%, with SEI capacity losses predominantly (80.8%) attributed to Li_2_O. Although only a limited electrolyte range was tested, CE showed a weaker correlation with LiF content. Thus, high CE values above 99% likely result from combined effects of both Li_2_O and LiF. To isolate the influence of fluorine, electrolytes such as LiClO_4_ in DME/anisole and LiClO_4_ in DOL/DME with LiNO_3_ were also evaluated, achieving CEs of 98.9% and 99.1%, respectively, with SEI analysis confirming high Li_2_O content. However, these electrolytes are also expected to produce LiCl and Li_3_N in the SEI, whose effects and contributions were not examined in these studies. Overall, exploring a broader range of electrolytes beyond fluorine‐based systems could be beneficial for advancing lithium metal battery performance.

#### 2.3.5 LiH/LiF in the SEI

Beyond the regular inorganic salts in the SEI layer, the LiH component has recently gained increased attention. Since its initial proposal by Aurbach in 1999, reports on LiH in the SEI have been scarce due to its high reactivity and the challenges associated with its detection [156]. However, recent studies have confirmed the presence of LiH in the SEI, revealing its unique properties.

Different electrolytes produce distinct SEI structures, and LiH has been observed in two main forms. First, LiH was identified as part of lithium dendrites using cryo‐TEM [157, 158]. This LiH is believed to form via the reaction between lithium metal and hydrogen gas, which originates from water impurities in the electrolytes and the decomposition of hydrogen‐containing solvents. Thus, even with super‐dry solvents, typical carbonate‐ and ether‐based solvents used in LMBs are prone to LiH dendrite formation, which leads to electrical isolation of lithium metal and capacity fade. A recent study showed LiH was also detected using MS‐D_2_O titration, indicating that LiH accumulation negatively correlates with the cyclability of practical LMBs [159].

LiH has also been detected as an inorganic SEI component [160, 161, 162].  In 2021, crystalline LiH was identified as a significant SEI component in both low‐ and high‐concentration LiFSI‐based electrolytes through synchrotron XRD and pair distribution function (PDF) analysis [160]. Additionally, LiF in the SEI exhibited expanded lattice parameters compared to standard LiF, suggesting the formation of a solid solution LiH_x_F_1‐x_. It was not until 2025 that ^19^F MAS NMR and XRD confirmed that this LiF corresponds to LiH_x_F_1‐x_ (H‐rich phase) and LiF_y_H_1‐y_ (F‐rich phase), which have been widely detected across different electrolytes [162]. Notably, high CE electrolytes such as 1 M LiTFSI in DOL/DME + 2wt% LiNO_3_, 1.5 M LiFSI in DME/TTE and 4 M LiFSI in DME showed higher amounts of LiH_x_F_1‐x_. Increasing hydrogen content expands the lattice parameters of LiH_x_F_1‐x_, potentially enhancing ionic conductivity. The higher hydrogen content in LiH_x_F_1‐x_ improves ionic conductivity and facilitates Li^+^ transport.

These findings confirm the presence and establish potential roles of LiH in the SEI. However, LiH is air‐sensitive and shares a face‐centered cubic (FCC) structure with similar lattice parameters as LiF (4.084 Å for LiH and 4.026 Å for LiF) [160], complicating its analysis. Understanding the formation of the LiH–LiF solid solution requires careful consideration of LiH behavior across different electrolyte systems. Furthermore, the role of LiF in the SEI should be reevaluated. Future strategies should aim to minimize LiH formation in dendrites while promoting the beneficial development of the LiF–LiH solid solution within the SEI.

#### 2.3.6 SEI Characterization

To uncover the precise architecture of SEI layers, it is essential to develop characterization methods that are both minimally invasive and offer higher resolution. Common surface characterization techniques used to analyze the SEI include SEM, XPS, cryo‐TEM, XRD, atomic force microscopy (AFM), and NMR [163]. These methods provide detailed insights into the morphology and chemical composition of the SEI layer.

Notably, XPS depth profiling with argon ion sputtering can deliver comprehensive information throughout the entire SEI. However, recent studies have shown that this technique may induce undesired reactions with SEI components, leading to artifact signals [164, 165, 166]. Cryo‐TEM offers high‐resolution imaging and allows for deeper investigation of the SEI's internal structure [143, 167]. For example, cryo‐TEM has revealed SEI swelling, with the degree of swelling strongly depending on the electrolyte composition. Increased SEI swelling has been correlated with a decline in battery performance. Building on these insights, in 2024, Choi's group investigated hexane as an anticorrosive additive to improve the calendar life of lithium metal batteries by preventing the decomposition of hexafluoroisopropyl methyl ether (HFME) in the electrolyte [168]. However, cryo‐TEM is limited to detecting very localized areas and primarily crystalline signals, as discussed earlier. Given these limitations, relying on a single analytical technique can lead to incomplete or misleading conclusions about SEI structure and composition. Therefore, a multi‐technique approach combining methods such as XPS, cryo‐TEM, and others is essential to obtain a more comprehensive and accurate understanding of SEI. Integrating complementary data helps to cross‐validate findings, mitigate artifacts, and capture the full complexity of the SEI.

In addition, while the aforementioned material characterization techniques are valuable for identifying SEI components, quantitative analysis remains challenging. Therefore, it is necessary to develop additional methods to achieve more accurate and comprehensive quantification. In 2019, Meng's group developed a quantitative titration method using titration gas chromatography (TGC) to measure inactive lithium metal (dead Li) within the SEI, demonstrating that dead Li is the primary contributor to capacity loss [169]. Although their study reported no detection of LiH in the SEI using TGC, subsequent investigations employing titration methods have identified LiH [159, 161, 170], highlighting ongoing controversy and the inherent difficulties in quantitatively analyzing SEI components. This variability is further complicated by differences in electrolyte formulations and cycling conditions. In addition, acid‐based titration methods have detected other SEI species such as ROCO_2_Li, Li_2_C_2_, RLi (R = CH_3_, C_2_H_5_, C_2_H_3_), LiF, phosphorous‐containing phases [159, 171]. More recently, in 2024, Gallant's group introduced an alcohol‐based titration method combined with Karl Fischer analysis to accurately quantify Li_2_O within the SEI layer [155]. Together, these analytical tools and methods advance our understanding and quantification of SEI chemistry, providing robust methodologies to probe SEI composition and identify key building blocks critical for achieving high cycling performance.

#### 2.3.7 Artificial SEI

Artificial SEI layers represent a promising strategy to overcome the limitations of naturally formed SEI in lithium metal batteries. By precisely engineering the interphase, artificial SEIs can provide enhanced mechanical stability, improved ionic conductivity, and better chemical compatibility with both the lithium metal anode and the electrolyte. This tailored protection helps suppress lithium dendrite growth, reduce side reactions, and extend battery cycle life. Lithium metal can react with various gases, such as N_2_ [172], F_2_ [173], NF_3_ [151], I_2_ [174], and S vapor [175], to create protective layers that shield lithium from moisture, solvents, and dendrite growth. In addition to gas‐phase reactions, artificial SEI layers can also be fabricated via film deposition and solution processing techniques [176, 177]. For instance, chemical vapor deposition (CVD) enables the fabrication of thin films with atomic‐layer thickness. In 2021, Lyden's group employed CVD to deposit an ultrathin zwitterionic polymeric interface with precisely controlled thicknesses ranging from 10 to 500 nm on lithium metal surfaces [178]. The zwitterionic moieties in this interface can tune the solvation structure and promote compact lithium deposition.

However, challenges remain in developing artificial SEIs that are scalable, cost‐effective, and durable under practical operating conditions. Achieving uniform coverage, optimal thickness, and long‐term stability without compromising battery performance requires advanced materials design and fabrication techniques. Moreover, compatibility with diverse electrolyte chemistries and manufacturing processes must be ensured for commercial viability.

### 2.4 Cycling Performance Under Different Conditions

From a practical standpoint, the cell response to cycling conditions and subsequent rest periods are critical factor that must be carefully evaluated and understood for serious technological consideration and implementation. Identifying limitations and failure modes is essential for ultimately developing effective solutions. These topics are discussed herein.

#### 2.4.1 Impact of Charge and Discharge Rates

Beyond investigating electrolyte composition and the SEI, practical operating conditions are critical to the performance and longevity of LMBs. For electric vehicle applications in particular, the battery must support charging and discharging at varying rates that correspond to the vehicle's driving cycle. Understanding how these dynamic conditions impact electrolyte behavior, SEI stability, and overall cell performance is essential for developing LMBs that can reliably meet real‐world demands.

**FIGURE 5:**
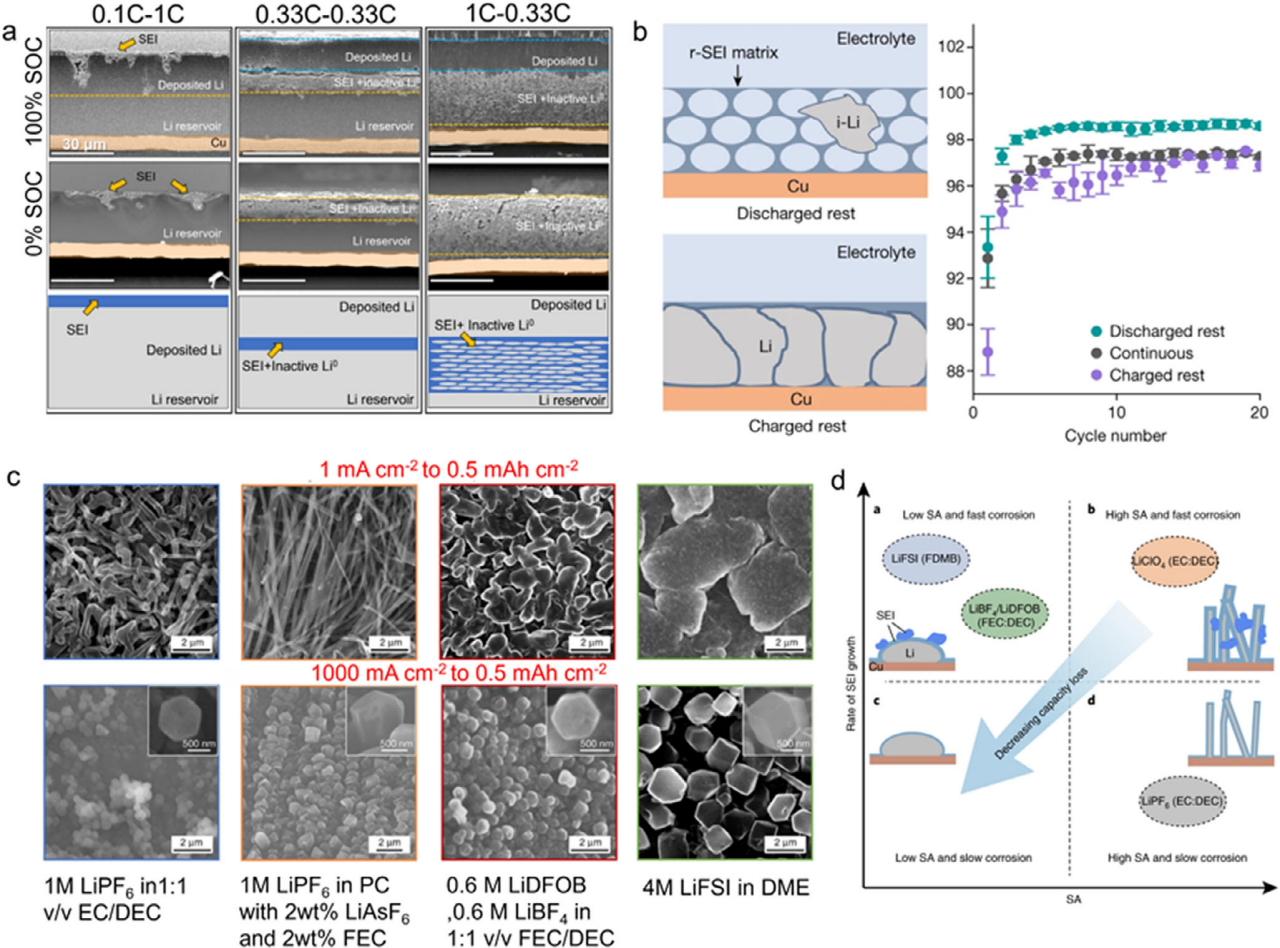
(a) Cryo‐SEM cross‐sections of Li anodes at various charge/discharge rates, with a schematic illustrating the buildup of inactive Li during cycling. Adapted with permission [184]. Copyright 2025, American Chemical Society. (b) Schematic showing the anode after calendar aging in the discharged state and charged state, performance of the first 20 cycles of LiFSI in DME/TTE LHCE electrolyte with different conditions. Reproduced with permission [189]. Copyright 2024, Nature Publishing Group. (c) Different dendritic lithium morphologies transition into identical faceted lithium polyhedral depending on electrolyte chemistry and current density. At 1 mA/cm^2^ in Li|Cu cells, lithium deposits as filaments or chunks varying by electrolyte type. At a high current density of 1000 mA/cm^2^ on ultramicroelectrodes, faceted lithium polyhedral form as filaments, rods, or columns depending on the electrolyte, with magnified images showing detailed particle shapes. Adapted with permission [191]. Copyright 2023, Nature Publishing Group. (d) Schematic of the relationship between the rate of SEI growth, surface area (SA) of Li and capacity loss of Li metal anodes in liquid electrolytes. Reproduced with permission [194]. Copyright 2021, Nature Publishing Group.

Today, a key goal for EV batteries is to achieve a driving range of over 300 miles, assuming an average speed of 30 to 75 miles per hour, which corresponds to a slow discharge rate of C/4 to C/10, this requires the battery to operate for 4 to 10 hours. In contrast, charging should be completed within 1 h or even as quickly as 10 min, demanding a fast charge rate. To meet these requirements, significant research efforts have focused on understanding and improving the charge and discharge rates of lithium metal batteries. Experiments show that the primary cause of cell failure at high charge current densities is the rapid formation of a highly resistive SEI layer intertwined with lithium metal, followed by continuous SEI growth into the bulk lithium and a sharp increase in cell impedance. This behavior has been observed across various electrolyte types [179, 180]. Additionally, studies indicate that ether‐ and carbonate‐based electrolytes tend to perform better under low charge and fast discharge conditions [181, 182, 183, 184, 185]. In 2016, Zhang's group found that fast discharge rates resulted in better performance compared to fast charge/slow discharge and equal charge/discharge rates in both Li|NMC cells with 1 M LiPF_6_ in EC‐DMC and anode‐free Cu|LFP cells with 4 M LiFSI in DME [181, 182]. Using 1 M LiPF_6_ in EC‐DMC, SEM images under low discharge conditions (C/10 and C/3, where 1C = 2 mA/cm^2^) show significant cracking on the lithium surface. This indicates that the plated lithium is not well protected by the SEI layer and that SEI continues to form during cycling, which can reduce cycling performance. In contrast, the SEI formed at higher discharge rates (1C and 2C) appears more robust. XPS analysis reveals that the SEI layer formed at higher discharge rates contains polyethylene carbonate [poly(EC)], which is absent in the SEI formed at the lower C/10 and C/3 discharge rates. Similarly, in 2021, Dahn's group demonstrated that a slow charge combined with a faster discharge rate (C/2.5 × x and C/x, x = 2 and 4, 1C = 2.66 mA/cm^2^) can optimize anode‐free cell performance using 0.6 M LiDFOB and 0.6 M LiBF_4_ in FEC‐DEC [183]. They attributed this effect to concentration gradients present at the lithium metal surface; slow charging minimizes the concentration gradient, thereby reducing lithium dendrite formation. Conversely, faster discharging increases the concentration gradient, which raises the current density at the tips of lithium protrusions, leading to preferential stripping of these tips. This process helps remove nonuniform lithium deposits and results in a relatively uniform surface at the end of discharge. Therefore, a slow charge and fast discharge protocol is favorable for cycling. Recently, it was reported that slow discharging leads to increased SEI formation and accumulation of dead lithium within the SEI, consistent with previous findings. Importantly, SEM and TEM analyses suggested that dead lithium within the SEI may form an electronically conductive network, which promotes lithium plating on the SEI layer and necessitates the formation of a new SEI layer above the deposited lithium (Figure 5a) [184]. As a result, SEI and dead lithium accumulate during cycling, causing a decline in cell performance. Consequently, to simulate fast‐discharging conditions, pulse discharge current was applied in a full cell protocol. In Li|NMC811 full cells with a 4.3 V cut‐off, cycling with a 1.32C discharge pulse (1C = 3 mA/cm^2^) for 60 s followed by a 180 s rest time showed an improvement of over 120 cycles in cycle life compared to cycling at 0.33C benchmark, representing a 48% enhancement.

In addition, pulse charge current can also improve the cycling performance [186]. During the plating stage, the continuous consumption of Li^+^ at the electrode surface creates a Li^+^ concentration gradient, which promotes the formation of Li dendrites. Higher current densities result in larger concentration gradients. This pulsed charging helps reduce dendrite formation by allowing metal ion concentration at the electrode surface to replenish during rest, thereby minimizing the depletion layer.

#### 2.4.2 Reconnecting Isolated Lithium and Ultrafast Pulse Deposition

To improve cell performance, recovering isolated Li in the SEI could be a promising approach, as the accumulation of isolated Li is a major contributor to capacity loss in high‐performance electrolytes [169, 187]. Notably, in 2021, Cui's group claimed that isolated lithium can be partially dissolved near the cathode side and plated back near the lithium side during discharging, providing an opportunity to reconnect the isolated lithium. Applying a short, high current density during the stripping step (e.g., 2 min at 3 mA/cm^2^) can help polarize the isolated lithium and promote its reconnection to the lithium anode, thereby improving cell performance. This effect is especially pronounced when applying a high current density from 2 to 5 mA/cm^2^, which can recover more isolated Li [188]. The same group also developed a different protocol using LiFSI in DME/TTE LHCE to recover isolated lithium in the SEI [189]. They applied a 12 h rest at open‐circuit voltage (OCV) in the discharged state and demonstrated an average CE of 98.2% during the first 20 cycles of Li|Cu half‐cells, compared to 96.9% for continuously cycled cells at 1 mA/cm^2^ with 1 mAh/cm^2^ (Figure 5b). Similar recovery was also observed in 1 M LiPF_6_ in EC/DEC + 10% FEC and 4 M LiFSI in DME at the same cycling conditions. The optical image, cycling results, XPS, and NMR data indicated that the organic components of the SEI dissolved and allowed the isolated Li to reconnect to plating during charging. It is important to note that SEI dissolution and chemical corrosion of isolated Li occur simultaneously during discharged resting at OCV. Therefore, while reconnecting isolated Li in the SEI can improve cycling performance, Li corrosion generates additional SEI and thickens the layer. Over time, SEI solubility can reach saturation during cycling, causing capacity recovery to decrease [190]. These finding highlights an important advancement by demonstrating the potential to recover isolated lithium within the SEI, a factor that has long been considered a major source of irreversible capacity loss. However, relying on SEI dissolution to recover isolated Li raises concerns about electrolyte consumption and overall cell stability. A robust SEI should minimize electrolyte decomposition and maintain structural integrity during cycling. Thus, this recovery approach may be more applicable to electrolytes with inherently low CE, where capacity loss is more severe. It is also worth noting that only a limited number of electrolyte systems have been tested with this method, and broader validation is needed. In summary, while the recovery of isolated lithium within the SEI seems possible, addressing its root causes and improvements in electrolytes offer a more compelling path to overcome this challenge.

Since the majority of capacity loss occurs due to SEI formation, another considerable strategy is to reduce or even avoid SEI formation altogether. In 2023, Li's group proposed super‐high plating current density to outpace SEI formation as well as avoiding mass transport limitations (Figure 5c). Specifically, plating current densities ranging from 50 to 1000 A/cm^2^ using an ultramicroelectrode (UME) geometry showed that lithium deposits preferentially as rhombic dodecahedra, with deposition kinetics claimed to surpass the SEI formation. This behavior has been consistently observed across multiple electrolyte systems exhibiting both low and high CE, as well as on diverse substrate materials [191]. Thus, ultrahigh current densities resulted in non‐dendritic lithium growth in the form of rhombic dodecahedra, provided that mass transport limitations are mitigated. A pulse‐current deposition protocol was employed to promote the formation of lithium rhombic dodecahedra as a nucleation template at ultrafast current densities, i.e. small amount of Li capacity was passed at high currents followed by plating at conventional current densities. For example, in Li|Cu cells with 1 M LiPF_6_ in EC/DEC, lithium was plated at 50 mA/cm^2^ for 0.05 mAh/cm^2^, followed by growth at 1 mA/cm^2^ for 0.95 mAh/cm^2^, achieving a total areal capacity of 1 mAh/cm^2^. This protocol resulted in a 5% CE improvement compared to cycling continuously at 1 mA/cm^2^ for 1 mAh/cm^2^ (20 cycles and CE with 80%–90%). Similarly, in an electrolyte of 0.6 M LiDFOB and 0.6 M LiBF_4_ in FEC/DEC, lithium was plated at 50 mA/cm^2^ for 0.05 mAh/cm^2^, followed by growth at 3 mA/cm^2^ for 2.95 mAh/cm^2^, reaching a total areal capacity of 3 mAh/cm^2^. This approach showed a 1% CE improvement compared to continuous cycling at 3 mA/cm^2^ for 3 mAh/cm^2^ (50 cycles and CE with 95%–98%). While these findings are intriguing, the modest improvements in CE and the specific electrolyte choices raise questions about the overall effectiveness of the protocol. For instance, advanced LiFSI‐based electrolytes—often lower in ionic conductivity—could exhibit a shorter Sand's time, making them more susceptible to dendrite formation. Moreover, because SEI layers were still observed on the plated lithium, it is unclear whether SEI formation was truly bypassed; although the authors attribute the SEI to contact between freshly deposited lithium and the electrolyte, this interpretation remains open to debate.

#### 2.4.3 Li Corrosion in LMBs

Corrosion of Li metal anodes involves both anode–electrolyte reactions and galvanic corrosion. Typical SEI layers are formed through anode–electrolyte reactions, which we have discussed in the above sections. Galvanic corrosion, also known as contact corrosion, is an electrochemical process that occurs when two electronically connected conductors form a galvanic couple within an electrolyte [192]. In Li metal batteries, this couple is formed by the Li metal and the current collector (typically Cu). Due to its lower electrode potential, Li functions as the anode and corrodes, while electrolyte reduction occurs on the Cu surface, leading to the formation of SEI. Investigation of the corrosion behavior of electrochemically deposited Li on a Cu current collector showed that reactive Li acts as the anode and inert Cu as the cathode [193]. Due to a relatively weak passivation of SEI on the Cu, electrons transfer from the Li to the electrolyte through the Cu, while Li^+^ diffuse outward through the SEI on the Li surface, resulting in corrosion. The morphological evolution of the Li deposits during this process is attributed to the Kirkendall effect. Galvanic corrosion of Li metal anodes leads to the loss of active Li and electrolyte decomposition. These parasitic reactions significantly degrade the capacity and cycling stability of Li metal batteries.

Several studies have also revealed that the calendar life of LMBs is closely related to the corrosion of lithium metal anodes. For example, Li metal was found to lose at least 2%–3% capacity after 24 h of aging, largely independent of electrolyte chemistry, which subsequently reduces cycle life [194]. Cryo‐TEM imaging confirmed that the capacity fade results from Li corrosion and continued SEI thickening. Effective electrolytes must therefore minimize both the SEI growth rate and the surface area of electrodeposited lithium (Figure 5d). Zhang's group demonstrated that the calendar life of LMBs is strongly influenced by both the lithium metal surface area exposed to the electrolyte and the mechanical stability of the SEI layers [195]. Minimizing this exposure and controlling the state of charge (SOC) during storage are crucial to reducing degradation. Using a LHCE based on LiFSI in DME/TFEO, they developed a mechanically robust SEI shell that withstands repeated lithium plating/stripping with minimal damage. This stable SEI structure is key to extending calendar life. Their results with Li|NMC811 cells demonstrated that storing cells at 0% SOC followed by 100% SOC significantly reduces lithium surface exposure, thereby enhancing battery longevity.

Chemical corrosion of LMAs, a key factor limiting their calendar life, was also investigated [196]. Studies have shown that its severity is more pronounced in the carbonate electrolytes versus ethers. This was demonstrated through investigation conducted in four typical electrolytes: bisalts electrolyte 4.7 M LiFSI + 2.3 M LiTFSI in DME, nitrate electrolyte 1 M LiTFSI in DME/DOL+ 2% LiNO_3_, carbonate‐based electrolyte 1.2 M LiPF_6_ in EC/EMD and LHCE 1.5 M LiFSI in DME/TTE. Using XPS, fresh SEI layers formed in bisalts, nitrate, and carbonate electrolytes contained similar components, mainly LiF, Li_2_O, and organic species. However, after 3 weeks of resting, while Li_2_O nearly disappeared in the carbonate‐based electrolyte, it remained stable in the ether‐based electrolytes, indicating that SEI layers in ether‐based electrolytes are more stable. Although XPS depth profiling provided valuable insights into SEI component evolution during corrosion, the exact SEI structure of lithium deposited from each electrolyte remains unclear. The inorganic SEI components, primarily Li_2_O, LiF, and Li_2_CO_3_, showed little variation among the three electrolytes, suggesting that the higher stability of SEI layers in ether‐based electrolytes may be due to differences in their organic components. It is worth noting that the porosity of electrodeposited lithium, as expected, can impact the corrosion rates, where lower rates were observed through stack pressure application to densify the deposited Li. However, uniformly applying high stack pressure across the entire electrode surface in battery cells can present additional technical challenges, potentially increasing costs and reducing the overall system energy density. A recent study suggested reducing Li corrosion by incorporating *n*‐hexane [168]. Specifically, *n*‐hexane was added to an LHCE using HFME as a diluent to mitigate lithium metal corrosion. The introduction of *n*‐hexane effectively suppressed lithium corrosion by acting as a kinetic barrier, preventing HFME from diffusing to the lithium surface through the swollen solid–electrolyte interphase.

These studies systematically examined Li corrosion in liquid electrolytes, focusing on two key factors: the surface chemistry of the SEI and the porosity of electrodeposited lithium. However, while significant progress has been made in understanding these aspects, the complexity of SEI nanostructure and its dynamic evolution under practical battery conditions remain unexplored. Future work should prioritize designing dense, stable and mechanically robust SEI layers that tightly protect the lithium surface and effectively mitigate corrosion, especially under elevated temperatures and long‐term storage, to bridge the gap between laboratory findings and real‐world battery performance.

#### 2.4.4 The Failure Modes of LMBs

The failure of LMB cells during cycling is primarily associated with dead/isolated lithium and SEI accumulation [169, 187], which continuously consumes electrolytes. This leads to a sudden capacity drop (“cell death”) in the cycling profiles. This phenomenon has been observed in both coin cells and pouch cells [21, 179, 197, 198, 199].

Coin cells provide significant advantages as a research platform for evaluating electrolyte performance. Their compact and straightforward design enables precise control over key experimental parameters such as electrolyte composition, current density, and cycling protocols. This level of control allows researchers to systematically investigate how different components and conditions influence electrochemical behavior, facilitating clear correlations between electrolyte formulation and cell performance. However, in coin cells, the electrolyte volume ranges from 40 to 100 µL, which is more than 50–75 times greater than that used in commercial Li‐ion cells. This large electrolyte volume can significantly extend the cycle life of coin cells. However, when the electrolyte amount is reduced, rapid cell death is often observed [198]. The reported CE of coin cells is also influenced by the cell hardware and assembly. Variations in cell configuration, assembly methods and materials used in coin cell can lead to different CE [200]. Therefore, we caution against claims of cyclic stability when coin cells are being utilized.

To address these limitations, establishing benchmarks that define experimental conditions relevant to realistic high‐energy cells is essential. Pouch cells are commonly employed in R&D laboratories for electrolyte testing because they better represent the form factor and size of commercial batteries [198]. They enable evaluation under conditions closer to real‐world applications, including realistic electrode stacking, electrolyte distribution, and thermal management. Additionally, pouch cells offer greater flexibility for testing various materials and configurations, as well as higher capacity for more meaningful performance and degradation analyses. Given their importance in electrolyte research, pouch cells are also used to analyze failure modes, helping to isolate electrolyte performance from external factors that may influence test results [199].

In 2019, Liu and Xiao's group demonstrated that 1.0 Ah 300 Wh/kg Li|NMC622 pouch cells using 1.2 M LiFSI in TEP/BTEF electrolyte can operate stably for up to 200 cycles before experiencing a sudden capacity drop (“cell death”) [21, 199]. To investigate cell failure further, 350 Wh/kg Li|NMC622 pouch cells with 1.5 M LiFSI in DME/TTE electrolyte were studied. XRD, SEM, and TEM analyses indicated that failure originated on the anode side rather than the cathode side. Additionally, varying the lithium anode thickness affected cell lifespan: SEM images showed that 100 µm Li accumulated more than twice the SEI compared to 20 µm Li, as well as supported by cell impedance measurements. The authors hypothesized the formation of two types of SEIs during cycling: “wet SEI”, which contains electrolyte, and “dry SEI”, which has no residual electrolyte. With a limited amount of electrolyte, thicker lithium deposits increase the surface area during cycling, causing the limited electrolyte to react and form new “dry SEI”. This accumulation of SEI layers ultimately leads to sudden battery failure. These findings suggest that both lithium thickness and electrolyte amount should be optimized together in cell design to minimize SEI accumulation. In addition, more research is needed on the detailed degradation mechanisms and molecular pathways of the SEI reactions.

Furthermore, applying suitable external pressure helps to homogenize the electrolyte and reduce SEI accumulation [201]. Studies showed pouch cell swelling reduced from 20%–40% down to 6%–8% under 110–248 kPa external pressure. Pressure mapping during cycling revealed hotspots shifting from the center to the edges, indicating higher pressure in the center during both charge and discharge. SEM analysis from full cells confirmed more intact lithium in the center, suggesting preferential lithium deposition from the NMC plate in that region during charging. Appropriately applied external pressure, enabled by hybrid fixtures, played a critical role in significantly reducing pouch cell swelling while enhancing the consistency of Li metal battery performance. It is important to note that pressure control is significantly more challenging in pouch cells compared to coin cells. Unlike coin cells, which have rigid, fixed casings that naturally maintain stack pressure, pouch cells use flexible, soft packaging that can easily deform during cycling. This flexibility makes it difficult to apply and sustain uniform external pressure across the cell. To address this, specialized external fixtures or mechanical systems are required to maintain consistent stack pressure throughout the battery's operation. These additional components increase the complexity of the experimental setup and can introduce variability in pressure distribution, making precise control and reproducibility more difficult to achieve in pouch cell testing.

These failure studies of pouch cells highlight that multiple interrelated factors critically influence the cycling performance and longevity of LMBs. Key parameters include electrolyte volume, which directly impacts SEI accumulation and electrolyte consumption; lithium metal anode thickness, which affects the effective surface area and the tendency for uneven lithium plating and heterogeneous SEI growth; and externally applied pressure, which enhances electrode–electrolyte interfacial contact, mitigates cell swelling, and helps suppress the formation of “dry” or non‐uniform SEI layers. Importantly, the chemical and electrochemical compatibility of the electrolyte with the lithium metal anode remains a fundamental consideration. Electrolytes that are less compatible with lithium promote porous, dendritic lithium deposition and unstable SEI formation, resulting in rapid capacity degradation and increased safety risks. Therefore, optimizing electrolyte formulations in conjunction with carefully controlled cycling conditions is essential to achieve stable, uniform lithium deposition and the formation of robust SEI layers, enabling long‐term, high‐performance cycling in practical lithium metal pouch cells.

## 3 Sodium Metal Anode With Liquid and Solid Electrolytes

Sodium metal has been studied for many years, dating back to the 1960s, first with the roll out of the high‐temperature molten Na metal–S battery and later ZEolite Battery Research Africa (ZEBRA) batteries, which operated at temperatures in excess of 250°C [202]. Room‐temperature liquid electrolyte‐type Na metal batteries were studied in the late 1970s motivated by the Li metal‐TiS_2_ cells reported by Wittingham and co‐workers [2]. Challenges with Na metal diverted focus to Na‐ion batteries; however, recent increased interest emerged as Na metal can be viewed as a means to boost the energy density of Na‐ion batteries especially given the low capacity of the hard carbon anode vs. that of graphite in the Li‐ion. Na metal has a lower melting point, elastic modulus and hardness than Li (respectively 371 K, 6.3 GPa, 0.69 MPa vs. 454 K, 11 GPa, 5 MPa). It shares with Li metal that large volume changes occur as the metal is plated and stripped, making it quite challenging to maintain a robust SEI presence throughout the charge/discharge process. There are numerous excellent reviews addressing various aspects related to Na metal batteries [203, 204, 205, 206, 207]; however, a critical and pinpointed address that guides advancements from a practical perspective is lacking. The purpose of this section is to address opportunities and challenges with Na metal and discuss guiding accomplishments in electrolyte design.

### 3.1 How Does Sodium Metal Stack in Comparison to Li

#### 3.1.1 Performance

One unique feature of using Na metal anode is the possibility to achieve very high plating/stripping coulombic efficiencies CE in the order of 99.9% in ethereal‐type electrolytes [16]. This is in stark contrast to Li metal, where reaching such values remains elusive. Although seemingly promising, this impressive CE is only achievable at low‐current densities where severe rate limitations are not only associated with plating but also with metal stripping [208]. The low CE at increasing stripping rates were linked with features of the stripped Na manifested in porous structures with large pinholes, which result in the formation of dead Na metal, thereby disrupting the connection between Na and the current collectors. In a prototypical ethereal electrolyte (NaPF_6_/ tetraethylene glycol dimethyl ether G4) [208], stripping at moderate rates led to a low CE (30%), whereas porous structures and pitting were evident after stripping at fast discharge (3 mA/cm^2^) compared to stripping at lower rates (0.5 mA/cm^2^) [208]. These porous structures caused the formation of isolated Na as stripping occurred near the current collector as opposed to the top surface. The isolated Na could however be recovered through application of lower stripping current densities, leading to an increased CE. Fast charging of Na is also problematic where excessive electrolyte decomposition takes place even in advanced Na electrolytes that tend to be more compatible. For example, the same study in NaPF_6_/G4 showed the presence of a 200 nm thick SEI after charging at 5 mA/cm^2^. Although we caution that in this case the conductivity of the electrolyte utilized can play a role in increasing the overpotential further driving electrolyte's breakdown, so future studies would be needed with a wide variety of highly performing Na electrolytes.

**FIGURE 6:**
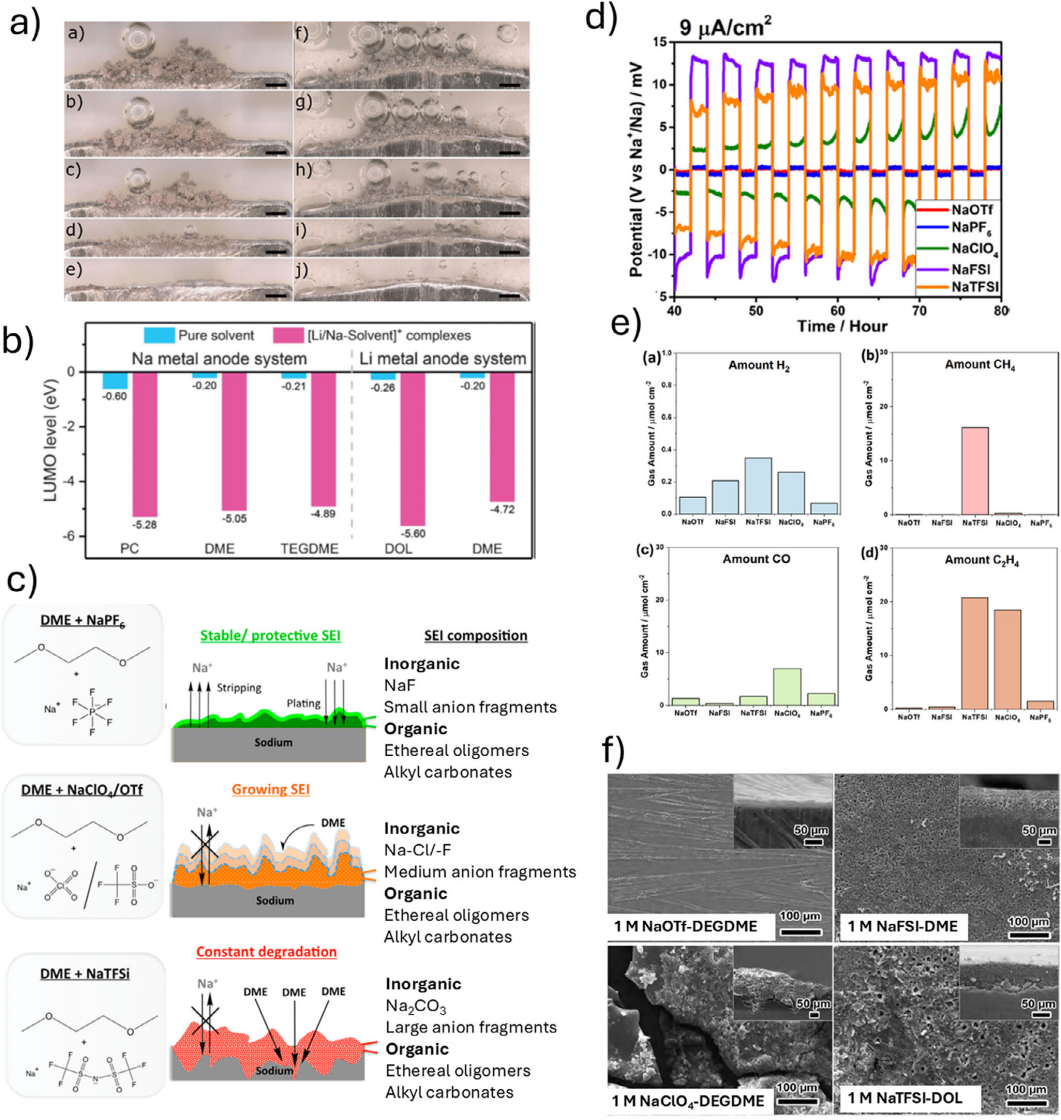
(a) Sodium depositions in FEC/DEC (a–e) and PC/FEC (f–j) electrolytes at a rate of 5.0 mA/cm^2^ for 1.0 mAh/cm^2^ of charge. Reproduced with permission [210]. Copyright 2017, American Chemical Society. (b) Comparisons among the LUMO levels of pure solvents and ion–solvent complexes in Na and Li metal anode electrolytes. Reproduced with permission [211]. Copyright 2018, Wiley‐VCH. (c) Illustration of SEI formation mechanism in different DME electrolytes. Reproduced with permission [216]. Copyright 2017, American Chemical Society. (d) Na plating/stripping in Na|Na cells. Reproduced with permission [217]. Copyright 2019, American Chemical Society. (e) Total amount of gas generated in different Na electrolytes in Na|Na cells. Reproduced with permission [217]. Copyright 2019, American Chemical Society. (f) SEM images of Na metal electrode surfaces after 50 cycles at 20°C in Na|Na cells. Reproduced with permission [218]. Copyright 2022, Nature Publishing Group.

In a similar fashion, in less compatible carbonate‐based electrolytes, Na plating was observed to occur accompanied by gas bubble evolution (NaPF_6_/PC), where also in this case, Na stripping was observed to occur near the current collector rather than the top surface [209], leading to dead Na. Gas bubble formation due to electrolyte decompositions were observed in addition to porous deposits in the electrolytes, and their intensities were correlated with the current densities. Mullins and co‐workers visualized the bubbles (Figure 6a) as a function of increased current densities, revealing that they became more apparent at 1 mA/cm^2^ currents, caused by exacerbations of the electrolyte decomposition due to increased plating overpotentials [210]. Nucleation density strongly influences dendrite morphology: higher densities at elevated plating rates yield shorter, more compact dendrites, whereas lower densities produce longer, more ramified structures [210]. Counterintuitively, high plating rates showed less erratic voltage spikes during cycling, likely because smaller dendrites detach more readily, forming isolated sodium that is less disruptive than the larger dendrites typical of lower current densities. This complexity underscores the need for careful evaluation of cycling performance to avoid misleading conclusions. It is important to note that the outgassing issue is not limited to esters and extends to ethers, similar behavior is also observed on Li metal. A study demonstrated the instability of ethers and showed that both Na and Li metal chemically react more rigorously in the electrolyte solutions vs. just the pure solvent, attributed to destabilization of the solvent molecules bound to the cation manifested in a lower occupied molecular orbital (LUMO) energy (Figure 6b) [211].

#### 3.1.2 Nature of Plated Na and SEI

One major difference between Li and Na metals is the nature of dendrites that form and higher propensity of Na to form isolated metal fragments. To explain, Na metal is more reactive than Li with the electrolyte components upon contact, attributed to weaker bound valence electrons, resulting in higher chemical reactivity [203]. Na dendrites were also shown to have much higher sensitivity to residual moisture, resulting in dendrite dissolution during cell rest without the application of a potential bias, whereas Li dendrites remain intact under similar conditions. That was reported in a variety of ethereal and Na salts (G4 and EC/DMC) solutions with NaPF_6_ and NaCF_3_SO_3_ [212]. Mechanically, Na dendrites were also found to be less mechanically robust, easily fragmenting and detaching, which leads to increasing formation of dead Na metal [212]. We thus caution that this behavior driven by Na intrinsic properties can initially mask underlaying dendrites formation during cell operation, ultimately leading to sudden and unexpected catastrophic failure. Therefore, care must be taken when designing and interpreting the electrochemical testing protocols of the electrolytes studied.

What further drives corrosion of Na is tied to the nature of the SEI. There is a consensus that the SEI produced in Na‐based batteries tend to be more soluble as opposed to Li metal SEIs. This is attributed to the lower acidity of Na^+^ leading to less stable organometallic SEI components (i.e. a study in NaPF_6_–cabonate electrolyte) [213]. Notably, the organic SEI constituents were quantified to be up to 3.26 times more soluble than the inorganic components [214], highlighting the severity of dissolution, which can be detrimental to the stability of the SEI. This leads to continuous electrolyte decomposition with cycling to failure. One possible pathway to overcome this serious issue is through maximizing the content of the inorganic component in the SEI, such as NaF and Na_2_O [16]. Also informed from Li metal, these inorganic components have high shear moduli of 31.4 and 49.7 MPa, which can support the formation of a denser Na metal, although the diffusivity of the cation in NaF was found to be several orders of magnitude less than that in LiF [215].

The formation of NaF has emerged as a key strategy in the pursuit of a robust solid–electrolyte interphase (SEI). Generally speaking, interest in fluorinated interphases mirrors the research trends in the Li metal area, discussed in Section 2. However, in the Na metal case, there is ample evidence that its presence does not guarantee a good SEI and cannot be simply correlated to improved performance, in some cases, it can even be counterproductive. The nature of the fluorine‐containing salt was found to play an important role in the homogeneity and distribution of NaF in the SEI. A study by Tarascon and co‐workers examined the SEI composition in a variety of typical electrolytes, NaClO_4_, NaTFSI, Na triflate, NaPF_6_, in DME solvent and correlated it to the Na plating/stripping performance and SEI impedance [216]. For example, NaTFSI produced high plating impedance, less pronounced NaF coverage, and severe Na corrosion with visible decomposition products that could be peeled off the metal. On the other hand, NaPF_6_ provided the most stable and uniform SEI with pronounced presence of a NaF layer. The perchlorate‐derived SEI containing NaCl was unstable, as evidenced by increased impedance—this was speculated to result from weaker bonding in NaCl versus NaF, which makes it less protective (Figure 6c). Similar trends were observed by Adelhelm and co‐workers, which emphasized the inferiority of TFSI^−^ and FSI^−^ anions in forming a good SEI, in stark contrast with their behavior on Li metal (Figure 6d) [217]. For example, the TFSI anion generated larger amounts of gas while NaFSI introduced the highest resistance among the salts studied (Figure 6e). A pronounced presence of Na_2_O in the perchlorate salt SEI was also found. Overall, the poorer‐performing salts exhibited higher chemical reactivity with Na metal, forming an SEI that was evident even after simple immersion in Na metal. A more recent study utilizing first‐principles computations showed that the NaTFSI reduction potential is too low such that ethereal solvent decomposition precedes NaF formation [218]. Interestingly, the same study proposed that excessive instabilities of FSI^−^ and ClO_4_^−^, evidenced in higher reduction potentials as compared with triflate and NaPF_6_, promote nonuniform plating morphologies in ethereal solutions, leading to inferior performance (Figure 6f). How a lower salt decomposition onset influences SEI chemistry and structure remains unclear. We speculate, however, that excessive gas evolution and other byproducts generated during SEI formation can compromise the SEI homogeneity and mechanical robustness. Notably, these trends differ from the Li metal systems, where FSI^−^ is widely regarded as an effective SEI former, and even perchlorate‐based electrolytes can enable stable cycling, albeit with lower CE than FSI‐based formulations [155].

Nonetheless, good performances were reported in FSI‐based electrolytes when utilizing high concentrations, that is, 5 M NaFSI/DME. However, the results reveal increased resistances in these solutions, resulting in CE fluctuations, surprisingly even at a very low current density of 0.56 mA/cm^2^ [219]. The negative impact of NaF was also seen on the sodiation/desodiation insertion anodes, where higher impedances were reported (i.e., Sn_4_P_3_ anodes), demonstrating that excessive NaF coverage is not preferrable outcome due to its resistive nature [220]. Beyond the primary Na salt being the main source of NaF, the solvent or other organic additive can be NaF formers. Absence of foundational understanding regarding the decomposition path and role of the organic compounds challenges the ability to select the appropriate chemistry. For example, the addition of FEC to 5 M NaFSI/DME degraded the performance [219]. In our opinion, one plausible reason in this case could be attributed to increased outgassing due to FEC decomposition, which can inhibit the formation of a dense and more homogeneous SEI composition. This is not far‐fetched given that in PC‐FEC‐PF_6_ solutions, random porous Na resulted due to outgassing [210], this outgassing was found to diminish the electrochemical active surface area and dislodge the deposited Na [210].

Based on the issues related to NaF, we caution against the pursuit of NaF formation as a necessary descriptor of good performance and encourage studies that consider capturing the impact of all potential contributors to the SEI based on the electrolytes’ formulations.

Beyond fluorinated salts, recently NaH (Young modulus 45 GPa) was observed in Na metal SEI [221]. In NaClO_4_/PC electrolytes (with/ without FEC additive), the presence of NaH was notable, and based on a slower NMR T1 relaxation time, NaH was inferred to have lower ionic conductivities as opposed to NaF. NaH formation mechanism and its impact and role remain unexplored; however, seemingly its higher resistivity is problematic. Since LiH was also observed in ethereal solutions on Li metal, a potentially similar formation mechanism may be at play in the Na metal case, which motivates further detailed studies.

### 3.2 Electrolytes for Na Metal

#### 3.2.1 Traditional Solvents Based

**FIGURE 7:**
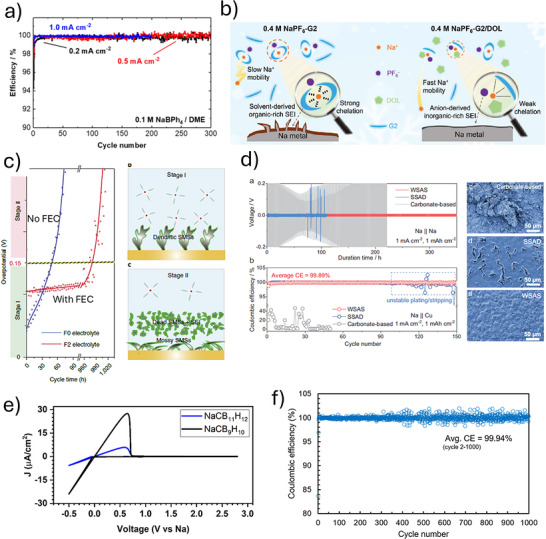
(a) Charge–discharge voltage curves of Cu/Na cells with NaBPh_4_/DME. Reproduced with permission [224]. Copyright 2019, Wiley‐VCH. (b) Schematic illustration of the mechanism of improved LT Na reversibility by the DOL‐diluted electrolyte. Reproduced with permission [227]. Copyright 2024, Wiley‐VCH. (c) Correlation between overpotential and Na microstructure in 1 M NaClO_4_/propylene carbonate (PC) with 2% fluoroethylene carbonate (FEC) (abbreviated as F2) and without FEC (F0) electrolytes in Na|Cu cells, and the corresponding morphological evolution schematics, both exhibit an overpotential critical transition voltage of around 0.15 V. Reproduced with permission [221]. Copyright 2020, Nature Publishing Group. (d) Electrochemical plating/stripping reversibility and characterizations of the Na metal plating on Cu in different electrolytes including Voltage–time profiles of Na|Na symmetric cells and Na plating/stripping Columbic efficiency (CE) of cycling performance in Na||Cu asymmetric cells at 1.0 mA/cm^2^ with a capacity of 1.0 mAh/cm^2^, SEM images of Na plating on the Cu with WSAS, SSAD, and Carbonate‐based electrolytes after 50 cycles. Reproduced with permission [239]. Copyright 2024, Wiley‐VCH. (e) The electrochemical stability of sodium carborane electrolytes. Reproduced with permission [240]. Copyright 2023, Royal Society of Chemistry. (f) Half cell cycling data for Na_4_(B_10_H_10_)(B_12_H_12_) in half cell with Al pressed pellet and Na_9_Sn_4_ counter electrode. Reproduced with permission [241]. Copyright 2024, Nature Publishing Group.

Electrolyte design principles are very similar to those implemented for Li metal, wherein, as discussed above, the presence of inorganic NaF‐type content is needed to achieve high coulombic efficiencies. That said, strategies used in Li metal electrolyte developments have been applied to enable improved cycling performances of Na metal. Esters type electrolytes have poor compatibility with Na, reacting even upon contact prior to electrochemical cycling [222, 223], so ethereal solvent solutions are by far the most effective and dominant types and it covers mono and multidentate solutions such as triglyme and tetraglymes, first demonstrated to result in 99.9% CE in NaPF_6_ (1 M) close to a decade ago by Cui and co‐workers without the use of additives or special electrode coating. Electrolytes using other common salt analogs to those used in Li metal chemistries include NaFSI, NaTFSI, NaBF_4_, sodium trifluoromethanesulfonate NaTFSI, sodium bis(fluorosulfonyl)imide (NaFSI), sodium bis(trifluoromethanesulfonyl)imide Na triflate, and NaClO_4_. There has been recent interest in considering halogen‐free electrolytes, several of which have shown promising Na plating/stripping cycling efficiencies, although they have not yet surpassed fluorine‐containing systems such as PF_6_^−^‐based electrolytes. For example, dilute NaBPh_4_/DME solutions (0.1 M) were reported with CE approaching 99.85% by Yamada and co‐workers [224]. However, CE stability was not examined in that work; fluctuations during extended cycling (Figure 7a), including values exceeding 100%, and a decrease in CE upon modestly increasing the salt concentration to 0.7 M (despite improved bulk conductivity) raise questions about possible adverse effects of a boron‐derived SEI on Na metal. These observations underscore the need for careful characterization of boron‐containing interphase species using surface‐sensitive techniques such as XPS. Since some of us first reported the advantages of hydrides for enabling reversible metal anodes more than a decade ago, the research community has increasingly investigated borohydride‐based electrolytes with reactive metals [11]. A recent report examined NaBH_4_ and showed a high coulombic efficiency (99.67% for NaBH_4_/DME) and high cyclic compatibility with Na metal [225]. However, the study does not clearly substantiate the practical value of this electrolyte. First, the coulombic efficiency was only evaluated using a modified Aurbach method, which can obscure capacity losses during the early “activation” cycles; without CE% reported as a function of cycle number, overall performance cannot be reliably assessed. Second, the electrolyte appears to operate only at a single concentration, leaving its broader compatibility with Na metal unresolved.

Improvements of Na electrolytes were demonstrated utilizing cosolvents or cosalts. For example, mixtures of different ethers, incorporating either short‐ or long‐chain glymes were used to improve the properties of the electrolyte, such as anodic stability. For example, 1 M NaPF_6_ in a solvent mixture of diethylene glycol dimethyl ether (G2) and tetraethylene glycol dimethyl ether (G4) was used to induce increased anodic tolerance for the operation of P2‐/O3‐type layered oxide cathodes through improved CEI [226]. We caution, however, that despite the apparent performance improvements, the inherently limited anodic stability of ethers remains a key barrier. Thorough evaluation of CEI effectiveness is therefore needed, including tests such as voltage hold (“floating”) and soaking experiments.

On the other hand, ether‐based blends have been particularly effective for improving low‐temperature operations through incorporating low‐melting point cosolvents such as dioxolane (DOL). For example, triflate [218] and NaPF_6_ [227] were used in G2/DOL mixtures to demonstrate highly efficient low‐temperature operation. DOL incorporation in 0.4 M NaPF_6_/G2 solution enabled improvements in the conductivity and diffusivity of the Na^+^ and resulted in a high CE of 99.9% at very low temperatures. DOL also promoted formation of a more robust SEI, evidenced by a smoother morphology and a higher Young's modulus (1.1 GPa) compared with G2 alone (0.3 GPa; Figure 7b). This improvement was attributed to DOL weakening G2–cation coordination, which in turn facilitated PF_6_^−^ decomposition, consistent with a reduced LUMO energy level [227]. Although not discussed, one challenge we view with these systems in general is the limited solubility of the salt at low temperature, which helps explain the choice of low salt concentration to allow low temperature operation. While lower concentration may benefit cycling at low temperature regimes, it can compromise the performance at room temperature.

Mixed anionic systems were used to improve the properties of the electrolyte through achieving synergistic effects. In one study, NaF and Na_2_CO_3_ were added to the electrolyte to suppress the dissolution of the corresponding SEI components [228]. However, the practicality of this strategy is questionable, as the delicate equilibrium may be readily perturbed during cell operation, potentially causing undesired and uncontrolled precipitation. Most approaches rely on conventional soluble salts as additives. For instance, the addition of NaBF_4_ (0.1 M) to NaPF_6_ (0.9 M) solutions was shown to promote the formation of sheet‐like B–O SEI suggested to aid in repairing the SEI cracks [229]. However, understanding the functional role and impact of these sheets is still lacking, and the absence of half‐cell cycling data makes it difficult to clearly assess the specific contribution of NaBF_4_. It is worth noting that the earlier decomposition onset of BF_4_^−^ resulted in a more beneficial Cathode Electrolyte Interphase (CEI). Additive salts could incorporate other less reducible cations such as K^+^ and Li^+^, either having the same or different [230] anion compared to the principal Na salt [230, 231, 232, 233], even in carbonate‐based electrolytes. The goal of incorporating different cations varied; it aimed to produce a shielding effect to prevent Na dendrite formation through absorption of plated Na protrusions, that is, Li and KTFSI additive [231, 232], while in other instances its main aim was to have these additives actively participate in forming inorganic rich SEI, as was reported for LiTFSI additive in a NaClO_4_ carbonate electrolyte [233]. It is important to note that the first report demonstrating the used of additive salts that have the same anion as the principal salt but with a different cation was first implemented in Mg battery electrolytes in 2012, where the addition of LiBH_4_ or NaBH_4_ to Mg(BH_4_)_2_ electrolyte resulted in dramatic improvements in the plating/stripping current densities [11].

Informed by strategies implemented in the Li metal area, highly concentrated [219, 234, 235] and locally highly concentrated electrolytes [236] were pursued to further increase the inorganic content in the SEI. NaFSI is commonly combined with ethereal solvents and diluents, such as those proven effective for Li metal, including bis(2,2,2‐trifluoroethyl) ether (BTFE) [236], 1H,1H,5H‐octafluoropentyl‐1,1,2,2‐tetra‐fluoroethyl ether (OTE) [237], 1,1,2,2‐tetrafluoroethyl‐2,2,3,3‐tetra‐fluoropropyl ether (TTE). However, the conductivity in these solutions remains limited, and the path to address achieving higher cycling rates is not clear. We suggest caution when introducing F source through organic solvents due to a lack of understanding of the decomposition pathway and the resulting products’ profile. For example, fluoroethylene carbonate (FEC) can improve the cycling performance of Na metal in carbonate electrolytes [238]. However, as discussed in the previous Section 3.1, FEC also promotes gas evolution and may not be an ideal NaF‐forming additive. In a NaClO_4_/PC electrolyte, in situ ^23^Na MRI showed that adding FEC led to a minimal presence of isolated (“dead”) Na metal, whereas in its absence isolated Na progressively accumulated [221]. This behavior was further linked to an increase in deposition overpotential, attributed to enhanced electrolyte decomposition and buildup of isolated Na in FEC absence (Figure 7c). Although FEC provides a clear initial benefit, it also led to an increase in the deposition overpotential at later cycles and ultimately resulted in failure (Figure 7c), indicating that the same underlying degradation processes still occur, but are delayed rather than eliminated.

Recently, weakly coordinating solvents emerged as a way to drive the formation of inorganic rich SEI through the formation of contact ion pairs (CIP) and aggregates (AGG). This trend again follows those being applied for Li metal batteries, as discussed in Section 2. A notable advancements were reported with the use of diethoxyethane (DEE), where high CE (99.89% average, 99.94% using the Aurbach method) was obtained in 2 M NaPF_6_/DEE electrolyte, attributed to a uniform SEI that has inorganic content of Na_2_O and NaF (Figure 7d) [239]. The improved SEI produced less porous plated Na, which contributed to lowering the fluctuations in the measured CE relative to DME‐based electrolytes, DEE also enhanced the low‐temperature performance. Despite these impressive gains, Na‐free full cells operated to 4 V with Na [Ni_1/3_ Fe_1/3_ Mn_1/3_]O_2_ (NFM) still lost ∼20% of their capacity within the first 100 cycles. This occurred although the electrolyte showed good cathode compatibility via CEI formation. Further studies are needed to understand the degradation mode in these electrolytes so appropriate solutions can be devised.

#### 3.2.2 Solid State and Beyond Volatile Solvents

In order to minimize or even eliminate the volatility concerns of the solvents utilized in Na metal batteries, ionic liquid solvents discussed in Section 2, or solid state electrolytes are poised to offer a potential solution (Section 5). Additionally, these provide an opportunity to understand Na metal interface in the absence of typical organic layers, as would be in the case of inorganic solid state electrolytes. Ionic liquids are molten salts at room temperatures constituting of an organic cation and an inorganic anion such as TFSI^−^ and FSI^−^ [109]. The organic cations found to be more appropriate for use with Na metal include pyrrolidinium and phosphonium [242, 243]. The high viscosity of these solvents typically makes efficient room temperature operation challenging, so good Na metal plating/stripping can mainly be achieved at elevated temperatures. Despite the relatively low conductivity, Forsyth and co‐workers, who have been pioneering the use of ILs with Na metal anodes, reported improved ability to cycle Na metal at room temperature in methylpropylpyrrolidinium (C_3_mpyr) FSI, citing good interfacial properties as manifested in faster charge transfer that to some extent countered the impact of the low conductivity [244]. However, the overpotential remained relatively high, which still limits the rate capabilities. Thinning of the ionic liquids has been typically used to increase the conductivity through reduction of the viscosity; this indeed improves Na plating/stripping rate performance [245]. However, thus far, high overpotentials have been proven very challenging to overcome in solutions dominated by ionic liquid, so further discoveries are needed to overcome this challenge. On the other hand, inorganic solid‐state electrolytes, as will be discussed in Section 5, fully eliminate concerns associated with liquid electrolytes, such as unfavorable organic SEI and SEI solubility issues, in addition to elimination of the desolvation barriers. Very high Na conductivities to a record 70 mS/cm at 300 K and 1.0 transference number were reported by Orimo and co‐workers in Na_2_(CB_9_H_10_)(CB_11_H_12_) [246]. The lower melting point of Na metal, as opposed to Li metal, can have a positive impact on maintaining better contact between the metal and electrolyte during plating/stripping and minimizing voiding issues that ultimately lead to dendritic‐type structural evolutions. *Closo*‐borates were particularly shown to support good cycling of Na metal anode by Remhof et al. [17, 247], and good intrinsic compatibility was also verified for Na *closo*‐carborates in a systematic study that addressed the potential impact of impurities and synthetic conditions (Figure 7e) [240]. The low reactivity of the *closo*‐borate Na_4_(B_10_H_10_)(B_12_H_12_) versus the sulfide counterpart manifested in a much thinner interphase with Na_9_Sn_4_ anode [248] and higher coulombic efficiencies even when cycled at impressive high capacities as demonstrated by Meng and co‐workers (7 mAh/cm^2^, 1 mA/cm^2^, pressed Al powder current collector; Figure 7f) [241]. Nonetheless, the choice of electrolytes compatible with Na metal remains very limited and further exploration of improved and new designs would be highly encouraged.

## 4 Multivalent Metals: Magnesium and Calcium Anode With Liquid Electrolytes

While Li metal has dominated the conversation around high‐energy anodes, Mg metal represents a multivalent alternative whose interfacial chemistry and design constraints differ in important ways. The persistent interest in Mg anode is rooted in a compelling set of intrinsic advantages over Li anode. From a safety perspective, Mg metal has an intrinsically lower tendency to form dendrites, reducing the risk of internal short circuits. For space‐constrained applications, such as portable electronics and electric vehicles, Mg metal anodes offer a higher volumetric capacity. Lastly, magnesium is significantly more abundant and geographically distributed than lithium, supporting long‐term resource availability. Yet, this promise has been perpetually tempered by long‐standing fundamental electrochemical hurdles that have slowed progress. The divalent nature of Mg^2^^+^ leads to sluggish interfacial kinetics, a consequence of its strong solvation, high desolvation energy, and a complex two‐electron transfer process for its electrochemical reduction [249]. This is compounded by severe interphase limitations, as Mg tends to develop native or electrolyte‐derived surface layers that are blocking to Mg^2^^+^ transport unless specifically engineered, contributing to a high nucleation overpotential that hinders uniform plating.

Exacerbating this challenge of surface passivation is the extreme sensitivity of Mg metal to common impurities, a practical consideration when it comes to evaluating the intrinsic vs apparent electrolyte behavior. Specifically, trace water and oxygen readily react with Mg metal to form blocking films of Mg(OH)_2_ and MgO, making a strict absence of these contaminants (<10 ppm water) critical for meaningful electrochemical assessment. The effect of any other salt or solvent‐derived impurities is often uncertain, but their accumulation at the anode can trigger detrimental side reactions. Common workarounds used in the literature, like in situ scavengers or electrochemical conditioning (“pre‐activation” or “conditioning”), merely act as a patch [250, 251, 252, 253, 254], potentially introducing new variables and obscuring the intrinsic behavior of the system. Further, from the practical perspective, only a rigorous purification and handling of electrolyte components is likely to offer a permanent solution.

To deconstruct these interconnected challenges, this section summarizes recent mechanistic and experimental advances that bear directly on practical Mg anodes. We will trace each critical bottleneck by examining the underlying mechanisms of failure, the design strategies developed to mitigate them, and the practical limitations of those strategies, focusing on how electrolytes solvate Mg^2^^+^, how interfaces form and fail, how metal microstructure influences deposition, and which engineering approaches are most promising going forward. Ca metal will also be briefly discussed, as generally it has similar challenges as the Mg metal.

### 4.1 The Solvation Shell

The quest for a reversible Mg anode has been historically shaped by a restrictive assumption born from experimental failures. Electrolytes obtained by simply transferring conventional Li‐ion components to Mg batteries, such as PF_6_^−^ salts and carbonate solvents, irreversibly passivate the metal surface and led to the widespread view that a protective solid electrolyte interphase (SEI) was unattainable [255, 256]. This constraint guided early electrolyte development pioneered by Aurbach and co‐workers toward systems with intrinsic compatibility with bare Mg metal, such as complex organochloride mixtures derived from Mg‐compatible Grignard reagents. This foundational period established two principles: ethers provide the most reliable solvent environment for Mg^2^^+^, and chloride anions are particularly effective at maintaining Mg surfaces electrochemically active [257]. However, despite the advantages of early chloride‐based systems, these suffered from corrosivity, limited oxidative stability, and poor compatibility with high‐voltage cathodes [257, 258]. Without losing sight of the lessons learned, efforts to circumvent these challenges initiated a transition toward halide‐free and simple electrolyte systems, pioneered by Mohtadi et al. with the development of Mg(BH_4_)_2_ and the introduction of weakly coordinating *closo*‐borates such Mg(CB_11_H_12_)_2_ electrolytes [11, 12, 13], which still defines much of current research. Within this framework, recent work continues to focus on developing new Mg‐compatible anions, as well as on engineering electrolyte formulations that employ commercially available salts, such as Mg(TFSI)_2_.

Mechanistically, the reversibility of Mg plating/stripping is intimately connected to the composition of the Mg ion complex approaching the electrolyte‐metal interface during the metal deposition/dissolution process. Compared to Li^+^, molecules within that first solvation shell are more tightly bound and strongly polarized due to the higher charge density of Mg^2^^+^ (1.28 *e*/Å^3^ vs 0.54 *e*/Å^3^), and this is supported both computationally [259] and experimentally [13] in the difficulty of thermally desolvate salts containing Mg‐coordinated solvent molecules. Unless carefully engineered (see below), the tightly bound solvation shell raises the desolvation barrier at the Mg surface, slowing charge‐transfer kinetics and increasing the overpotential for metal deposition, relative to monovalent systems. Further, species in the primary solvation shell are strongly polarized by Mg^2^^+^ and are exposed to highly reducing transient Mg^+^ ions and nascent Mg atoms created during the electron transfer process. Consequently, the reductive stability of the species bound to Mg^2^^+^ and the ease with which this coordination environment can reorganize prior to electron transfer directly impact deposition kinetics and the extent of parasitic side reactions.

#### 4.1.1 Custom‐Designed Standalone Anions

In the absence of a protective layer (see section 4.2), ethers such as tetrahydrofuran (THF), glymes (1,2‐dimethoxyethane (DME) or diglyme (G2)), and their derivatives remain the predominant solvent systems due to their inherent compatibility with Mg metal and their balanced donor numbers and dielectric constants [260]. These solvents provide solvation environments that balance salt dissociation against desolvation energetics, supporting reversible Mg plating at reasonable efficiency. Historically, electrolyte development focused on pairing these ethers with reductively stable anions to limit anion‐based parasitic chemistry. However, achieving the high performance of recent systems required a shift toward anions that are also weakly coordinating to enhance Mg^2+^ mobility.

**FIGURE 8:**
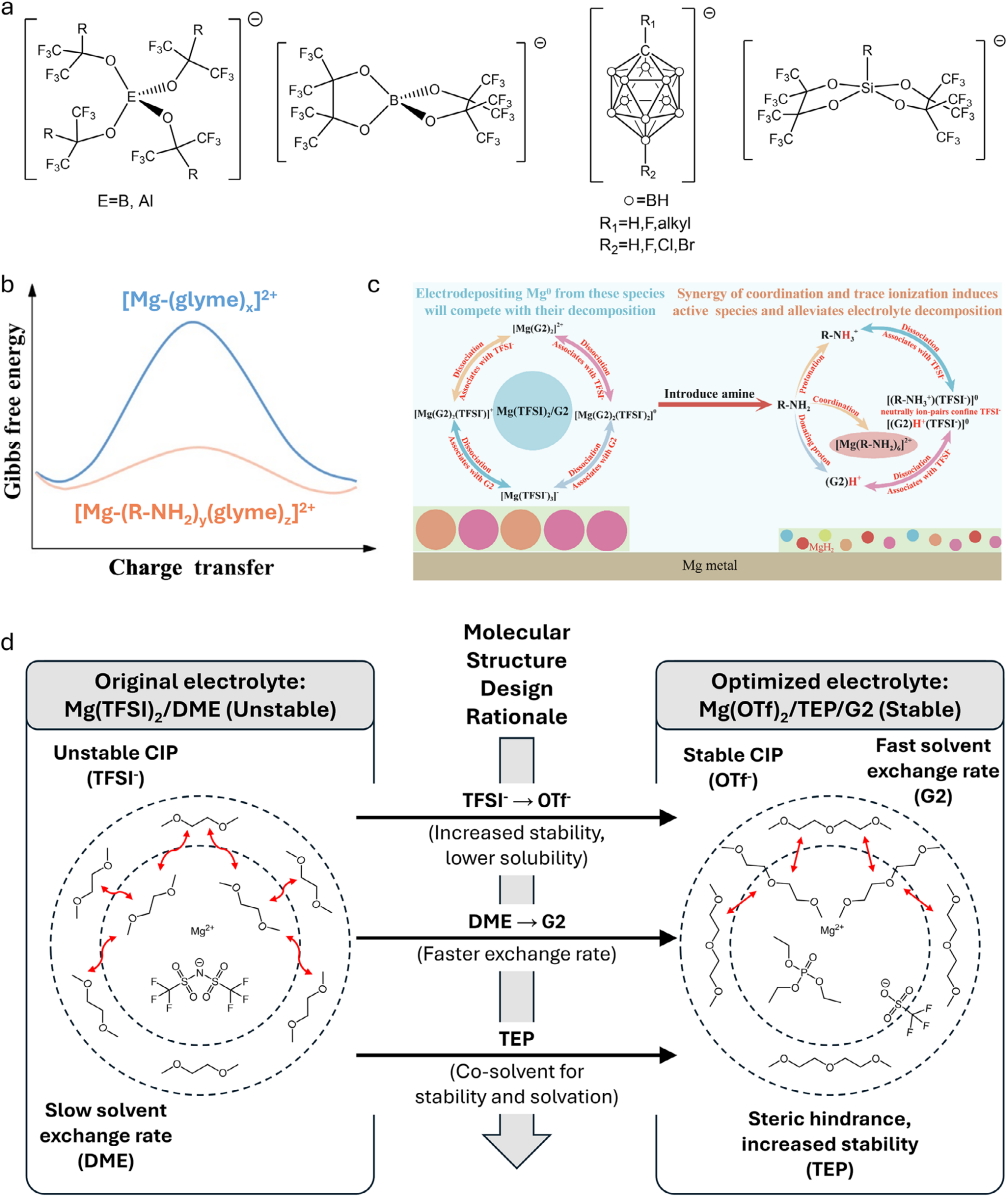
(a) High performance weakly coordinating anions for Mg electrolytes. (b) Transfer barrier reduction affected by glyme displacement from Mg^2+^ primary coordination sphere by primary amine co‐solvents. Adapted with permission [270]. Copyright 2024, Royal Society of Chemistry. (c) Proposed solution speciation MgTFSI_2_/G2 electrolyte before and after primary amine co‐solvents addition, including amine autoionization processes. Reproduced with permission [270]. Copyright 2024, Royal Society of Chemistry. (d) Rational electrolyte design via solvation shell engineering. The strategy evolves from the unstable Mg(TFSI)_2_/DME baseline to a robust Mg(OTf)_2_/G2 system with trialkyphosphate additives, promoting fast ligand exchange and thermodynamically stable contact ion pairs.

Over the past decade, several advanced anion families have emerged to meet these criteria (Figure 8a): fluoroalkoxyborates (Mg[B(hfip)_4_]_2_, where hfip = hexafluoroisopropoxyl) [261], and Mg[B(O_2_C_2_(CF_3_)_4_)_2_]_2_ [262]) pioneered by Fichtner and Zhao‐karger et al. and one of the most studied in the advanced anions family; fluoroalkoxyaluminates such as Mg[Al(hfip)_4_]_2_; [263] and carbaborate cluster anions Mg[CB_11_H_11_X]_2_ where X = alkyl, F, Cl, Br [13, 264]. All these anions combine exceptional reductive stability with minimal coordination to Mg^2+^, typically leading to high ionic conductivity and Coulombic efficiency of Mg deposition and stripping exceeding 99%. Most recently, a family of pentacoordinated silicate anions featuring fluorinated substituents has been introduced [265]. With a Coulombic efficiency on par with the initial reports of other anions (97%), the full potential of these silicate‐based electrolytes will likely be established as they undergo broader benchmarking within the research community.

#### 4.1.2 Solvent Engineering and Additive‐Driven Coordination

Despite initial setbacks in developing suitable electrolytes based on less specialized simple Mg salts such as MgTFSI_2_, interest in these systems persists due to their commercial accessibility and relatively low cost. Behind this continued interest lies evidence that some anions are significantly more robust toward Mg metal in its free, uncoordinated form compared to when coordinated to Mg^2^^+^ in species such as contact ion pairs or higher‐order aggregates [256, 266]. However, recent studies have refined this perspective, suggesting that the goal is not the absolute elimination of ion pairing, but rather to modulate the cation's coordination environment to simultaneously prevent anion‐derived interfacial passivation and maintain a low energy barrier for desolvation [267, 268]. As such, design responses toward enabling the use of the commercially available simple salts targeted achieving an optimal balance between solvation strength, ion pairing, and desolvation kinetics.

Restructuring the Mg^2+^ solvation sphere with additives has proven to be a key strategy for enabling these simple salts. Wang et al. found that co‐solvents with amine groups can displace ether molecules from the primary solvation shell, lowering desolvation barriers (Figure 8c), directly accelerating charge‐transfer kinetics (Figure 8b) and outpacing the parasitic anion decomposition process to achieve half‐cell cycling Coulombic efficiencies above 99.5% at low overpotentials (<50 mV) [269]. These benefits, however, are offset by practical issues like corrosivity and H_2_ evolution, which have been linked to proton exchange processes originating from amine autoionization [270, 271]. While these issues were alleviated upon anion switch from TFSI to triflate (TfO), the low oxidative stability of amines compared to ethers remains a challenge [271]. Beyond amines, the more oxidatively stable trialkylphosphates, such as trimethylphosphate (TMP) [272] and triethylphosphate (TEP) [273], initially introduced by Yang et al., have also been shown to enhance the kinetics through solvation shell reorganization, albeit without any of the shortcomings of amines, posing as a more promising approach (Figure 8d). Note that by altering the species that are reduced at the interface, both amine and phosphate additives also fundamentally dictate the nature of the resulting surface layer, a topic that will be detailed in the following section. Enhanced charge transfer kinetics are also attainable in additive‐free electrolytes through the use of multicomponent solvents, as evidenced by the superior performance of MgTFSI_2_ in DME/G2, compared to DME or G2 alone [274], and the high‐rate capability of Mg(CB_11_H_12_)_2_ in DME/G2 [275]. These results underscore the potential of multicomponent solvent systems to tailor the desolvation energy landscape, particularly when coupled with modern computational tools to guide and accelerate discovery [276, 277].

#### 4.1.3 Mixed‐Anion Strategies

The integration of multiple anion species offers a distinct pathway to manipulate the Mg^2+^ coordination environment, decoupling bulk transport from interfacial chemistry by combining anions with differing donor strengths. The classic addition of chloride to weakly coordinating electrolytes, for example, facilitates charge transfer by forming bridged [Mg_x_Cl_y_]^n^^+^ complexes that promote smooth deposition, albeit with well‐known trade‐offs [256, 278, 279]. Borohydride salts (BH_4_^−^) have been employed similarly, though their low oxidative stability limits their application to fundamental studies [266].

Iodine additives offer another powerful example, enabling stable cycling in MgTFSI_2_/DME electrolytes through a dual mechanism. In solution, they form electroactive species through I_3_^−^ complexation with Mg^2+^ that improve charge‐transfer kinetics and low the deposition overpotential [280]. Concurrently, they promote the in situ formation of a Mg^2+^ conductive MgI_2_‐rich interphase that ensures long‐term stability and reversibility [281].

### 4.2 Engineering the Interphase on Mg Anode

Once the Mg complex ion reaches the metal surface and even when desolvation is optimized, some coordinated species may still be reduced, forming a solid layer. Its chemistry and transport properties will determine whether deposition proceeds steadily or is throttled by resistive films, and whether deposited Mg remains reversible or becomes trapped under a blocking barrier. Historically, such layers were considered detrimental, exhibiting poor Mg ion permeability that rendered electrolytes non‐functional. This perspective changed with the seminal discovery that an interfacial layer formed in Mg(BH_4_)_2_ electrolytes could support stable Mg deposition and stripping [282]. This finding paved the way for the modern viewpoint that now guides the field, whereas a properly engineered interphase is not an obstacle to be avoided, rather the enabler of a new generation of high‐performance and chloride‐free electrolytes.

#### 4.2.1 Regulating Decomposition in Commercially Available Simple Salts

While this layer is commonly referred to as a solid electrolyte interphase (SEI) in Mg batteries by analogy to lithium‐ion systems, the comparison warrants caution. The validity of this direct analogy remains an open question, given the fundamental differences between Li^+^ and Mg^2+^ in terms of charge density and coordination behavior, as well as the disparate electrolyte design spaces from which these interphases emerge. Thus, well‐established strategies for Li‐ion SEI formation may not be directly applied to Mg; instead, the Mg interphase must be engineered through electrolyte design tailored specifically to the unique features of multivalent chemistry.

The need for interphase engineering arises primarily in electrolytes that are otherwise incompatible with the Mg anode due to passivation. The most prominent example is the commercially available MgTFSI_2_/glyme system, which is known to passivate the anode surface and has been widely used as a benchmark. The resulting interphase is now better understood, typically evolving into a thick and resistive layer composed of a mixture of inorganic species (MgF_2_, MgS_x_, MgO) and organic fragments from solvent co‐reduction accompanied by gas generation [126, 267, 270, 276, 283, 284, 285]. This layer grows uncontrollably during cycling and progressively blocks Mg^2^^+^ transport, leading to large interfacial resistances [272]. Consequently, in these systems, the main target to obtain a better performing interfacial layer is regulating the decomposition of electrolyte components by applying a number of key strategies.

**FIGURE 9:**
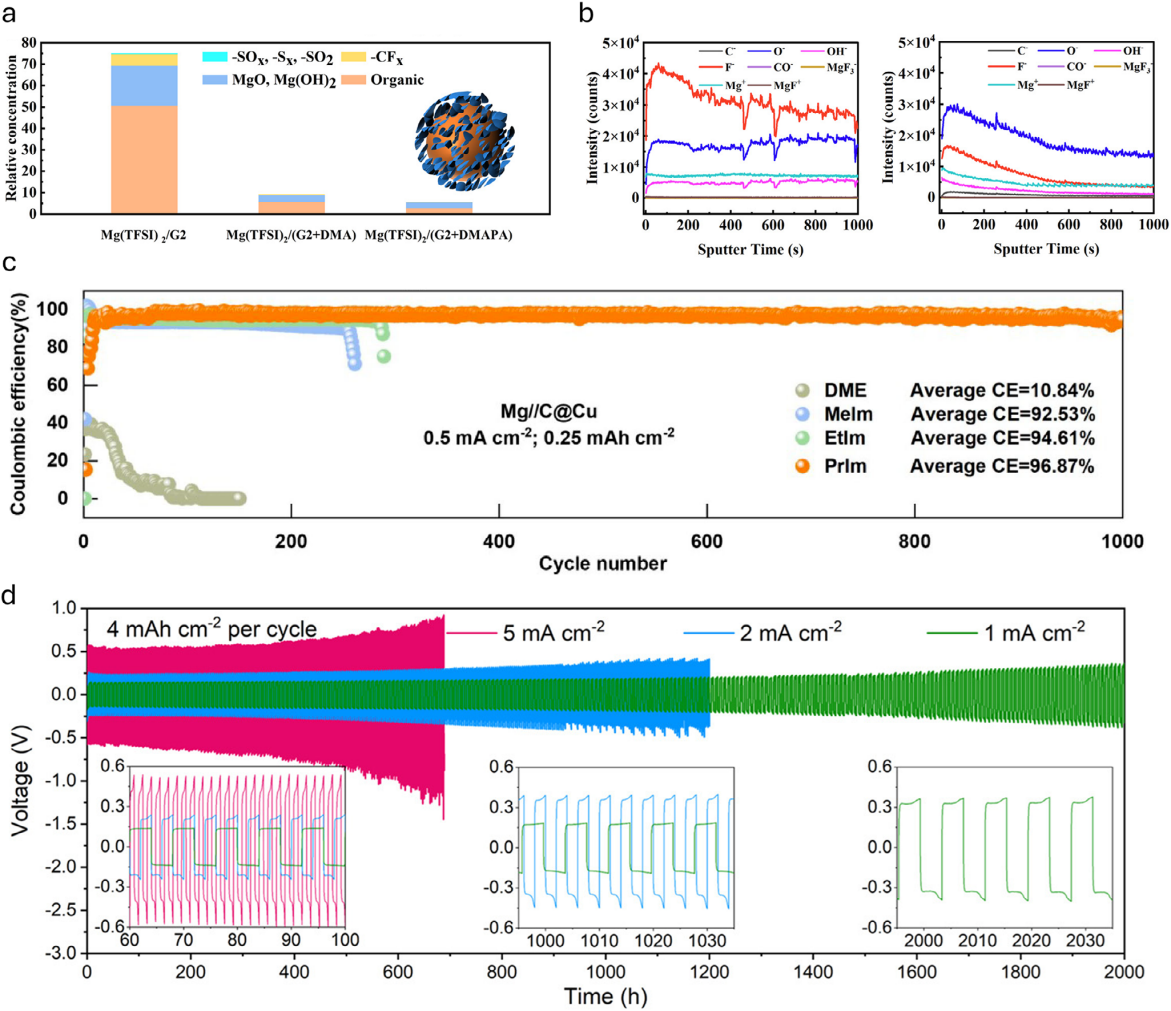
(a, b) Effect of 3‐dimethylaminopropylamine (DMAPA) co‐solvent on Mg metal interfacial layer composition and thickness on MgTFSI_2_/G2 electrolyte. Reproduced with permission [270]. Copyright 2024, Royal Society of Chemistry. (c) MgTFSI_2_/DME electrolyte performance with and without imidazole‐based co‐solvents on Mg//C@Cu (carbon‐coated on copper foil) cells. Reproduced with permission [285]. Copyright 2025, Wiley‐VCH. (d) Mg||Mg cells cycling of Mg(OTf)_2_/G2: TEP at various current densities with an areal capacity of 4 mAh/cm^2^. Reproduced with permission [26]. Copyright 2025, Elsevier.

One of the most direct approaches involves introducing functional additives to manage the interfacial reduction pathway. As discussed previously, amine co‐solvents enhance the charge transfer kinetics by modifying the Mg^2+^ solvation shell, with the direct consequence of mitigating the uncontrolled decomposition pathway and promoting a thinner, more stable and self‐limiting SEI composed of a mixture of organic and inorganic species (Figure 9a,b) [270, 286, 287]. In the limit, where the amine is within the solvent molecule, such as in amine‐ethers MPA (3‐methoxypropylamine) or in M2 (dimethoxyethylamine), the SEI contains an increased organic content and it has been suggested that the increasing number of phase boundaries and vacancies assist in Mg‐ion diffusion, notably with no simple correlation found between SEI fluorine content and performance [267]. Other ligand‐rich cosolvents, such as 1‐alkylimidazole additives, also yield thinner and denser organic‐rich interphases [284, 285]. Short alkyl substituents (methyl or ethyl) show a significant improved performance compared with the additive‐free system, reflected in a decreased in Mg//C@Cu asymmetric cell overpotentials (≈0.3 V vs >2.0 V at 0.5 mA/cm^2^) and average CE (92.53% and 94.61% vs 10.84%), via DME‑derived films (Figure 9c) [284]. A longer alkyl substituent, as in 1‐propylimidazole (PrIm), leads to a thinner and more uniform layer dominated by additive‐derived organics, and improved cycling stability. This is demonstrated in symmetric cell cycling (0.5 mA/cm^2^ and 0.25 mAh/cm^2^), where the cycle life increases from 250 cycles with shorter alkyl groups to over 1000 cycles with PrIm (Figure 9c) [285].

**FIGURE 10:**
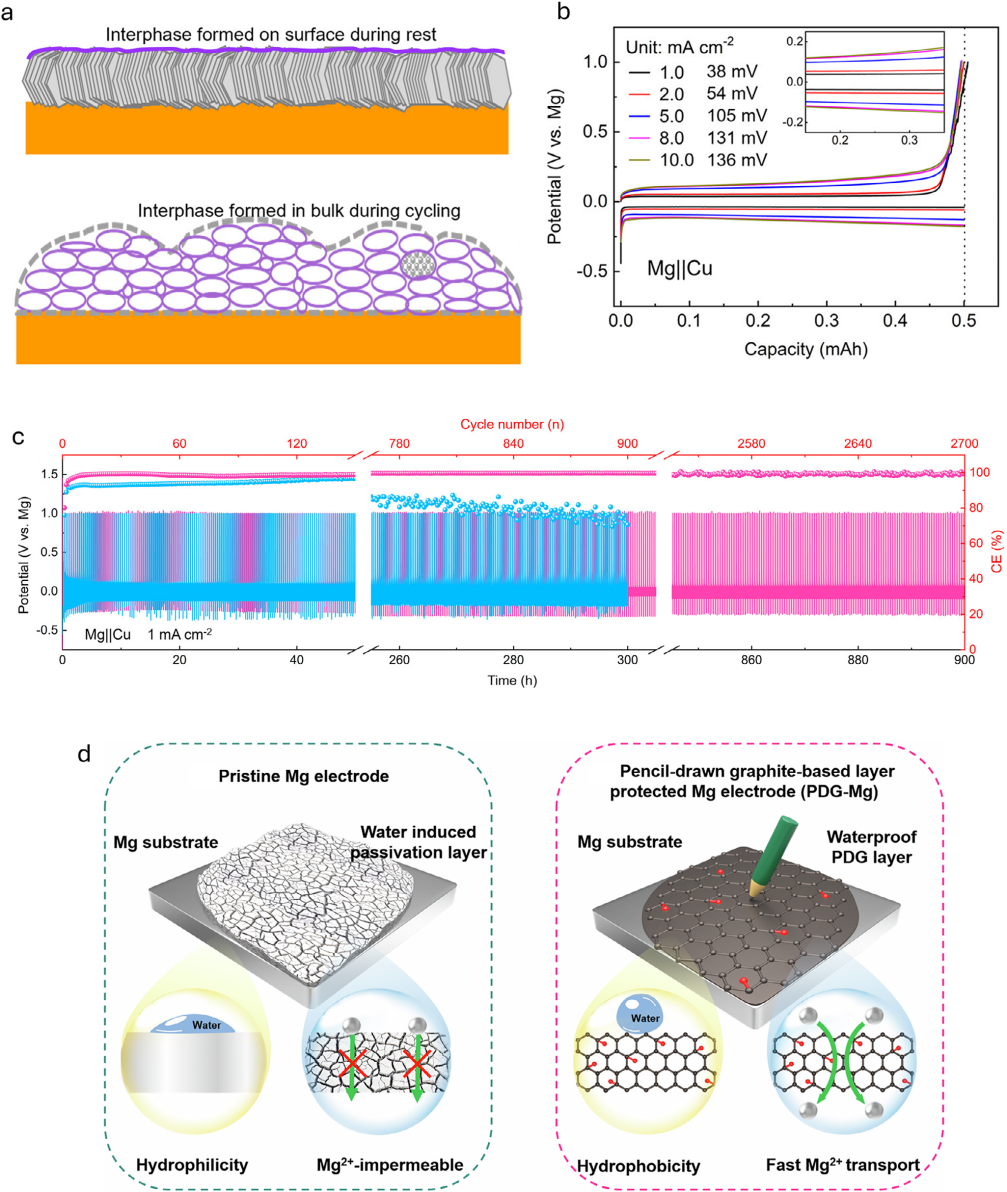
(a) Comparison of the interphase‐controlled Mg deposition morphologies in Mg(OTf)_2_/G2:TEP (up) and Mg(TFSI)_2_/DME:TEP (down) electrolytes. Adapted with permission [26]. Copyright 2025, Elsevier. (b) Voltage profiles of a Mg||Cu asymmetric cell using the Mg[B(hfip)_4_]_2_ in DME/THF electrolyte at current densities ranging from 1 to 10 mA/cm^2^. Reproduced with permission [276]. Copyright 2025, Royal Society of Chemistry. (c) Cycling performance comparison of Mg||Cu asymmetric cells using the Mg[B(hfip)_4_]_2_ in DME (blue) and in DME/THF (pink) electrolytes at 1 mA/cm^2^. Reproduced with permission [276]. Copyright 2025, Royal Society of Chemistry. (d) Schematic illustrating waterproof ion‐conductive protection provided by a pencil‐drawn graphite layer (right) compared to the water‐induced passivation on pristine Mg (left). Reproduced with permission [303]. Copyright 2024, Nature Publishing Group.

In a similar manner, organophosphorus additives like trimethyl phosphate (TMP) also fulfill the dual role of modifying the solvation environment and serving as a direct precursor for the interphase [272]. In a MgTFSI_2_/DME electrolyte, it promotes the formation of an inorganic‐rich interphase including Mg_3_(PO_4_)_2_ and Mg(PO_3_)_2_ that demonstrated an improvement of the interfacial Mg^2+^ transport, with a reported R_SEI_ of 279 Ω. The addition of lithium triflate (LiOTf) further improves cycling stability, extending the lifetime in symmetric cells from 150 to 450 h (0.5 mA/cm^2^ and 0.1 mAh/cm^2^) without altering the nature of the SEI [273]. However, at these longer cycling times, the overpotential increased from 80 mV to 580 mV, suggesting a progressively rising interfacial resistance due to continuous additive consumption for SEI reformation. While structurally similar to TMP, Nazar and co‐workers showed that triethyl phosphate (TEP) yielded a very different interfacial layer [26]. This layer is free of TEP‐derived components, either due to the suppression of TEP breakdown or formation of soluble decomposition products upon reduction. Furthermore, TEP assists in decreasing TFSI‐related components in the SEI by lowering the concentration of contact ion pairs (CIPs) in solution. Despite the resulting SEI being thinner than that formed in MgTFSI_2_/DME, Coulombic efficiency is limited to 70%, likely due to the continued formation of F^−^ and S^2−^ species in the interphase during Mg plating. This limitation was overcome by the same group through a complete solvent reformulation into a co‐ether phosphate electrolyte (CEPE), which introduced the fluorinated co‐solvent BTFE (bis(2,2,2‐trifluoroethyl) ether) as a protective shield that physically avoids the reduction of TFSI anion via preferential adsorption onto the Mg surface [283]. The Mg/CEPE electrolyte enabled remarkable cycling stability of >7000 h on a symmetric cell at 2 mA/cm^2^ and 2 mAh/cm^2^, clearly superior to MgTFSI_2_/DME/TEP (12 h) and MgTFSI_2_/DME (<2 h). In a further optimization, this system was rationally evolved into the MgOTf_2_/G2/TEP electrolyte, leveraging the higher reduction stability of the OTf anion compared to the TFSI anion, as well as the faster solvation shell exchange rate of G2 compared to DME [26]. STEM‐EDS imaging and depth‐profiling TOF‐SIMS studies of Mg plating concluded that this system allows near‐free‐interphase magnesium deposition, forming an ultrathin interphase at rest that is not carried into the Mg bulk and that does not affect the behavior of the Mg surface during deposition/stripping (Figure 10a). This type of behavior is reminiscent of the impedance increase found in conventional Mg‐compatible electrolytes, where an absorption layer was proposed to form during rest that disappears upon dynamic electrochemical cycling [288, 289, 290]. Under these near‐free‐interphase conditions, this system provides a very high average CE for half‐cell Mg metal cycling (99.79%–99.96%) at practical current densities and areal capacities (2–5 mA/cm^2^ and 1–10 mAh/cm^2^) (Figure 9d), although overpotentials of >500 mV are required for operating at 5 mA/cm^2^. Given the commercial availability of its components and its remarkable electrochemical performance, this system represents one of the most promising recent advances in Mg batteries. Nevertheless, to fully unlock its practical viability, future efforts must be directed toward reducing overpotentials and stabilizing the cell impedance during extended cycling.

#### 4.2.2 Scavengers and Sacrificial Anion Strategies

At the opposing end of the Lewis acid/base scale, electron‐deficient additives are proposed to function as scavengers for Lewis basic byproducts. For example, the additive tri(2,2,2‐trifluoroethyl)borate (TFEB) in the halide‐containing MgTFSI_2_/MgCl_2_/DME electrolyte is thought to remove inorganic electrolyte decomposition byproducts such as MgF_2_ and MgO, resulting in a thinner and less inorganic SEI [291]. This observation invites speculation about the historical role of Lewis acids in conventional electrolytes. Beyond their established function of complexing strong bases like Grignard reagents or amides, it is plausible that any excess Lewis acid also contributed to enhanced interfacial stability through a similar scavenging mechanism, a potential benefit that has remained largely overlooked.

Rather than managing TFSI anion decomposition with additives, another strategy is to replace the anion altogether, leveraging anions that act as sacrificial agents to form a more stable and less resistive interphase through a controlled decomposition. For example, electron‐localized fluoroalkoxylates like PFTB^−^ (perfluoroterbutoxide) or HFIP^−^ (hexafluoroisopropoxide) favor the formation of “weakly solvated CIPs” due to stronger Mg^2+^–anion interactions. This tight pairing facilitates desolvation at the electrolyte‐electrode interface, driving the formation of a thin and dense MgF_2_‐rich layer [126]. However, this anion‐derived film ultimately hinders interfacial Mg^2+^ transport, which, combined with a low electrolyte bulk ionic conductivity of 0.2 mS/cm leads to high cycling overpotentials (150 and 200 mV) even at low current densities (0.1 mA/cm^2^). A more successful approach uses the [B(hfip)_4_] anion, which despite being initially considered Mg stable was later found to form interfacial layers in glymes, albeit much thinner than MgTFSI_2_ [292, 293]. With judicious solvent selection, Mg[B(hfip)_4_]_2_ can form an even more compact and MgF_2_‐dominated inorganic layer (in THF/DME) [276], or a robust organic‐rich layer (in 1,3‐dioxane) [277]. Both systems yield extended cyclability in symmetric cells and high ACE (99.5%–99.7% vs. 95.6% in DME), but the THF/DME system also achieves greatly improved cell overpotential (66 mV vs ≈240 mV at 1 mA/cm^2^; Figure 10b,c).

#### 4.2.3 Exogenous and Artificial Interphases ASEIs

An alternative approach to in situ SEI formation is to protect the Mg metal surface with exogenous interfacial layers designed to suppress parasitic reactions while enabling Mg^2^^+^ transport. Polymeric coatings were among the first approaches explored [294], but hybrid and inorganic ASEIs have become more commonplace due to their higher stability and stronger passivation. Inorganic ASEIs are typically based on protective layers composed of Mg alloys with semimetals such as In, Sb, Bi or Sn that are known to provide efficient Mg diffusion, which form via ex situ surface reaction of the Mg metal with suitable precursors [295, 296]. Hybrid systems combine inorganic stability with polymer flexibility by forming nanocrystalline MgCl_2_ domains in a polymer matrix that serve as Mg^2+^ conductive sites [297] or by simultaneously creating alloys and polymers [298, 299]. Although not always mentioned in these works, all the intermetallic components of these ASEIs are intrinsically mixed ion‐electron conductors (MIECs), possessing significant electronic conductivity in addition to their Mg^2+^ ionic conductivity, creating a risk of Mg deposition onto the ASEI surface. Therefore, a successful ASEI must be carefully designed to incorporate electronically insulating components like MgO, MgX_2_ (X = halogen), or organic components, to block electron transport. The precise control required for creating such multi‐component layers poses significant challenges for manufacturing scalability. Other engineered structures have been proposed that physically or chemically isolate the Mg metal while providing well‐defined pathways for ion transport. For instance, porous crystalline frameworks functioning as ion‐sieving membranes such as metallic [300] or covalent organic frameworks [301], as well as zeolites [302] have been applied as protective layers. These frameworks are designed with angstrom‐scale pores that selectively allow the passage of Mg^2^^+^ ions while physically blocking larger detrimental species from reaching the anode surface‐ an approach also applied for Li metal as outlined in Section 2.1. This mechanism prevents electrolyte decomposition and promotes uniform Mg deposition by creating a stable and selective transport channel. An even more unconventional approach discovered by Lie, Lu, Seh and co‐workers leverages a simple pencil‐drawing method (Figure 10d) to create a graphitic carbon layer on the Mg surface to create a water‐tolerant interphase that prevents the formation of the typical passivating MgO/Mg(OH)_2_ layer and enables stable cycling even after direct contact with water [303].

### 4.3 Mechanisms of Mg Anode Failure

The long‐term stability of Mg metal anodes stems from a complex interplay of electrochemical, interfacial, and material‐level factors. While Mg metal is fundamentally less prone to dendritic growth than Li metal, this perceived advantage masks a more complex reality. The failure of magnesium metal anodes is, in fact, a multifaceted challenge encompassing other degradation pathways, implying that focusing solely on the prevention of classic dendrites is an insufficient metric for success, as even blunt deposits have been shown to cause cell failure over repeated cycles. Assessment of these properties and limitations should precede any consideration of practical use so that they can be addressed appropriately; accordingly, they are in this section.

#### 4.3.1 Morphological Instabilities

**FIGURE 11:**
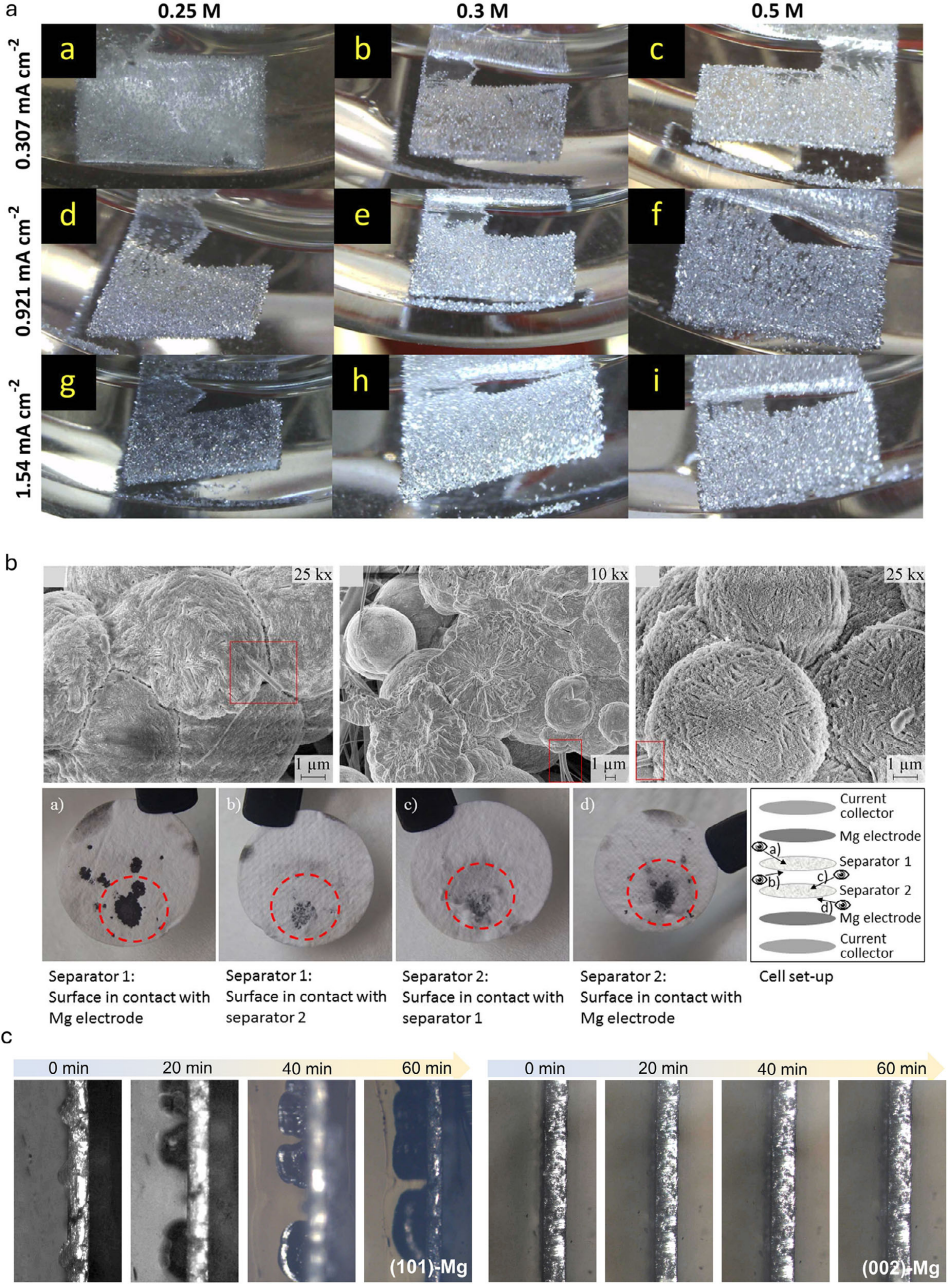
(a) Hemispherical Mg growth using Mg(HMDS)_2_‐2AlCl_3_ electrolyte in THF after 8 h of chronopotentiometry. Reproduced with permission [320]. Copyright 2024, Royal Society of Chemistry.(b) SEM images of Mg electrode after deposition step (up, left), of separator surface (up, center) and Mg electrode after oxidation step (up, right), and photographs of the two separators after a soft short‐circuit (down) by CV of a symmetric Mg cell using 0.3 M Mg(TFSI)_2_ in G2 electrolyte with a glass fiber separator. Adapted with permission [309]. Copyright 2018, IOP Publishing.(c) In situ optical images of the Mg deposition process on (101)‐Mg (left) and (002)‐Mg (right) electrodes at 3 mA/cm^2^ using APC (PhMgCl‐AlCl_3_ in THF) electrolyte. Reproduced with permission [319]. Copyright 2024, Wiley‐VCH.

Although less common that in Li metal, Mg dendrites can and do still form once Sand's time is exceeded [304], posing an arguably greater safety risk than their Li counterparts due to the superior mechanical properties of Mg metal. Mg dendrites exhibit significantly higher elastic modulus (27.1 GPa) [305] compared even to the bulk value of Li (∼6.8 GPa) [305], making them more resistant to mechanical suppression. Further, Mg hardness (0.5–1.5 GPa) [306] reduces the chances of dendrite yielding upon contact with the separator compared to the softer Li (0.5–175 MPa) [307, 308]. This combination allows Mg dendrites to more easily puncture even robust separators. A failure mode more unique to magnesium systems is the growth of non‐dendritic hemispherical deposits (Figure 11a). It has been demonstrated that even under conditions designed to suppress dendrites, Mg can evolve from initial 2D platelets into large 3D hemispherical structures that continue to grow and eventually cause short circuits (Figure 11b) [309, 310]. This morphology evolution highlights that globular deposits can generate sufficient localized pressure to penetrate or deform the separator. Another critical failure pathway involves the formation of “dead” Mg, electrically isolated Mg particles that typically originate from inhomogeneous dissolution of Mg anode [311]. Just like in other metal anodes, these detached particles can become trapped within the separator pores and grow within the separator structure once they reconnect with Mg anode on subsequent plating cycles, eventually leading to cell shorting [312].

These diverse failure modes in Mg anodes arise from processes that promote non‐uniform plating and heterogeneous nucleation [313]. While dendrite formation is fundamentally linked to the onset of diffusion‐limited conditions and quantified by Sand's time, hemispherical and dead Mg are primarily an interfacial phenomenon, rooted in the presence of a passivation layer. Hemispherical deposits originate from the low ionic conductivity of this layer, which impedes uniform Mg^2^^+^ flux and forces deposition to occur at a few electrochemically active sites rather than uniformly across the surface. While dead Mg is formed during the stripping cycle of any high surface area or morphologically unstable deposit (hemispheres being one example), where current density becomes focused at the base of the deposit, causing necking and detachment.

#### 4.3.2 Influence of Anode Metallurgy

Beyond electrolyte and interphase, which tend to be the main contributors to non‐uniform plating and heterogeneous nucleation, the intrinsic properties of the metal anode itself represent a critical yet often overlooked variable. While there are currently no systematic studies on the effects of Mg metal purity on its electrochemical performance, research on the Li metal anode provides a compelling precedent. In Li metal systems, it is well‐established that impurities present in commercial foils accumulate at the electrode‐electrolyte interface during cycling and act as preferential nucleation sites for heterogeneous deposition, leading to the formation of protrusions and dendrites that cause premature cell failure [314, 315]. It is very probable that similar impurity‐driven failure mechanisms are at play in commercial Mg foils. The presence of such impurities could act as a confounding variable in electrolyte development, where poor cycling stability attributed solely to the electrolyte might, in fact, be exacerbated or even initiated by heterogeneous nucleation on impurities within the Mg foil itself. In addition to purity, preliminary results reported by Mandai et al. showed that the Mg metal microstructure may also play a role on its cycling performance [306]. Warm rolling (300°C) provided a Mg foil with coarser grain size and lower residual stress density when compared to cold rolling (<300°C), thereby reducing features that can cause nonuniform current distribution and uneven anode utilization.

#### 4.3.3 Morphology Regulation Strategies

Strategies to mitigate these failure modes and promote homogeneous plating are applied at two distinct levels. The first is at the immediate surface through the creation of magnesiophilic interphases with elements like Sb, Bi, Sn, or In to guide uniform Mg deposition and lower kinetic barriers. Some of these layers are designed with a dual‐role of ASEI and guiding morphology and were discussed in the previous section. The second major approach aims to promote planar growth by favoring the electrodeposition of the low‐energy (0002) basal plane, which tends to be parallel to the electrode surface. This has been achieved using specific 3D host substrates like SnS_2_[316] or Ni(OH)_2_[317], incorporating electrolyte additives like tetrabutylammonium ions [311] or sacrificial n‐PrCl [318], or by chemically etching the Mg anode to expose the (0002) plane (Figure 11c) [319]. While promising, the efficacy of this crystallographic control at high current densities remains a critical question, as kinetic factors may overwhelm the modest energy differences between crystal facets. Determining the current density threshold for this transition and its relationship to Sand's time is essential for the successful implementation of this approach in full battery applications. Furthermore, factors such as the substrate used for deposition and the purity and microstructure of the Mg metal foil itself are often‐overlooked variables that can significantly influence nucleation and growth, and warrant more systematic investigation.

### 4.4 The Status and Challenges of Ca Metal

Calcium metal faces the same daunting challenges as magnesium in forming passivating interphases that inhibit reversible metal plating and stripping, as demonstrated by Aurbach and co‐workers [321]. Thus, many of the electrolyte advancements made in demonstrating reversible Ca plating/stripping were largely informed by the earlier developments in Mg batteries [6]. The large size of the Ca^2+^ (1.14 Å) vs. the Mg^2+^ (0.72 Å) results in low charge‐to‐size density (52 C/mm^3^) which is close to Li and less than half that of Mg^2+^ (120 C mm ^−3^). This suggests better mobility in solid‐state structures and lower desolvation energies and motivated studies of Ca metal anode batteries. The first report to demonstrate reversible metal plating in a conventional salt was reported by Ponrouch, Palacin and co‐workers in CaBF_4_ carbonate electrolyte, however, high temperatures (75–100°C) were involved, and the CE was relatively low [238]. Further improvements in the CE were informed by earlier developments in the Mg battery area by Mohtadi et al., where Ca(BH_4_)_2_/THF solution was shown by Bruce and co‐workers to support efficient reversible Ca plating and stripping in THF solutions at room temperature [322]. This electrolyte solution by far remains the most efficient electrolyte solution to date. Weakly coordinating salts, also directly learnt and transferable from the Mg field was the use of alkoxy borates [323, 324] and *closo*‐carboranes [325] that supported Ca plating/stripping at room temperature, albeit these solutions underperform in comparison to their performance with the Mg anode.

Beyond these salts, simple halides saw limited use because of their very low solubility in ethereal solvents. Recent examinations of CaI_2_/DME solutions showed Ca plating/stripping performance, albeit modest (overpotentials > 1 V at 0.02 mA/cm^2^) [326]. The authors attributed this behavior to a reduced diffusion barrier associated with a CaI_2_‐derived SEI [326]. The performance was further improved by increasing electrolyte conductivity through the addition of Lewis acids, most notably tris(2H‐hexafluoroisopropyl) borate (THFB). By interacting with the I^−^ anion, THFB promotes dissociation of CaI_2_, resulting in a Coulombic efficiency of about 71%. In general, the inability to achieve high CE in all Ca electrolytes can be linked to the tendency of Ca^2+^ to have a higher coordination number of 6–8 fold leading to Ca requiring desolvation energy near ca 1.5 that of Mg, contrary to what was believed earlier [324]. These limitations underscore a more severe metal passivation in the case of Ca compared to Mg.

Studies have recently shown that, similar to Mg metal anodes, SEI can also be present on Ca metal anode, wherein high inorganic SEI content can similarly result in increased overpotentials and poorer plating/stripping performances. For example, increased solvation of Ca^2+^ by high‐donor‐number solvents increased the organic fraction of the SEI and was correlated with improved Ca plating/stripping performance in symmetric cells in CaTFSI_2_ based electrolyte [327].

Demonstrating an efficient and reversible calcium metal anode remains a central research challenge, and this uncertainty continues to limit confidence in calcium's promise as a practical battery anode. Progress will depend on developing electrolytes and interphases that avoid surface passivation, since insulating reaction layers can quickly hinder calcium plating and stripping and lead to unstable, low coulombic efficiency. Future studies should also rigorously examine how the anode morphology evolves during deposition and dissolution, how the SEI or passivation layer builds up and changes with time, and how corrosion and parasitic reactions contribute to capacity loss. Finally, calcium anodes must be evaluated under realistic, dynamic cycling conditions, including varying current densities, areal capacities, and rest periods so that their stability and performance can be judged under operating regimes that reflect practical battery use.

## 5 Lithium Metal Anode With Solid‐State Electrolytes

The pursuit of next‐generation energy storage systems has centered on developing safer, more energy‐dense batteries to meet the demands of electrification and advanced electronics. All‐solid‐state lithium metal batteries (ASSLMBs) can represent a transformative approach, aiming to achieve these goals by replacing the flammable liquid organic electrolytes of conventional lithium‐ion batteries with a solid‐state electrolyte (SSE). This fundamental substitution not only enhances safety but also has the potential to enable the use of a high‐capacity lithium metal anode.

However, the realization of practical ASSLMBs is contingent on overcoming a complex interplay of material, interfacial, and mechanical challenges centered around the SSE. A successful electrolyte must exhibit high ionic conductivity at room temperature, comparable to liquid counterparts, to enable fast charging and high power output. Simultaneously, it must form a stable interface with the highly reactive lithium metal anode and high‐voltage cathode materials to prevent continuous decomposition and the formation of lithium dendrites, which can cause short circuits. Furthermore, the solid‐solid nature of the battery introduces unique mechanical stressors, where maintaining intimate contact, managing volume changes during cycling, and resisting fracture are paramount to long‐term stability.

This section provides a comprehensive overview of these critical aspects, focusing on all‐solid inorganic SSEs, which exhibit significant Li^+^ conductivity at room temperature without the use of solvents or plasticizers, unlike polymer SSEs. Since the impact of solvents on Li metal was discussed in Section 2, we refer readers to excellent reviews on polymers, as this topic is beyond the scope of the current discussion [328, 329]. We begin by surveying the primary classes of solid‐state electrolytes, including sulfides, halides, oxides, and hydroborates. For each class, we discuss their fundamental properties, ionic conductivity, and inherent compatibility with lithium metal. Next, we delve into the critical topic of the solid electrolyte interphase (SEI), examining the nature of naturally formed interphases and the strategic design of artificial interlayers to stabilize the anode/SSE interface. Finally, the section will address the pivotal role of mechanics, exploring how intrinsic material properties and the application of pressure influence everything from electrolyte densification and ionic conductivity to lithium deposition morphology and dendrite suppression. By systematically examining these interconnected challenges, the section aims to provide a clear perspective on the current state and future directions for the development of durable and high‐performance all‐solid‐state batteries.

### 5.1 A Survey of Solid‐State Electrolyte Families

#### 5.1.1 Sulfides

Since Kanno et al. developed the first LGPS‐type electrolyte (Li_10_GeP_2_S_12_) with a historical breakthrough in ionic conductivity (12 mS/cm at 27°C, sintered pellet) in 2011, sulfide electrolyte has gained widespread and growing research interest, becoming one of the most popular solid‐state electrolytes nowadays [330]. At present, sulfide electrolytes are typically categorized into three main types: (1) LGPS‐type, (2) argyrodite‐type, and (3) Li_2_S‐P_2_S_5_‐type.

**FIGURE 12:**
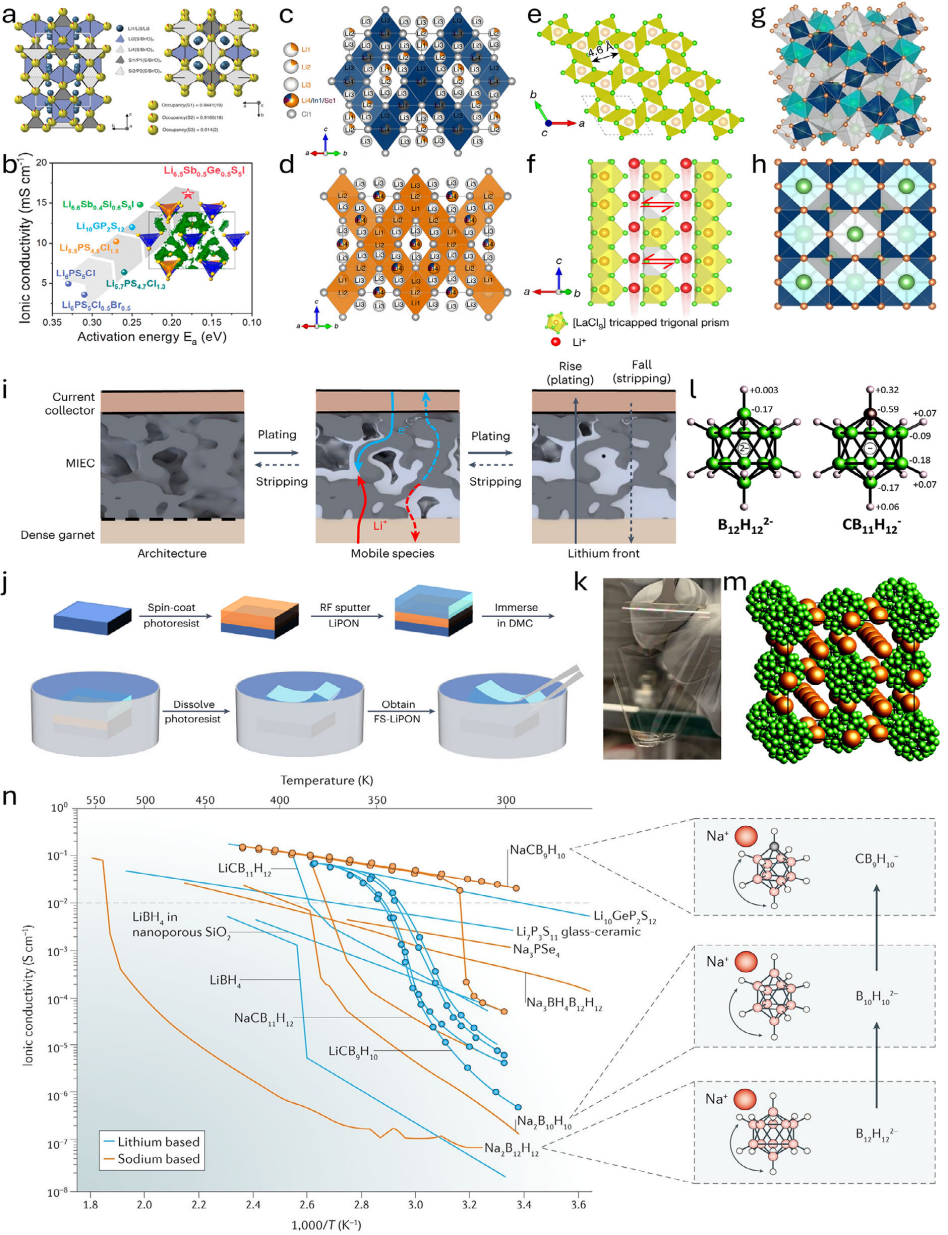
Overview of prominent solid‐state electrolytes. (a) Crystal structure of LSiPSBrO determined by Rietveld refinement, with S, Br, and O atom occupancies at S sites shown in yellow, green, and red, respectively. Reproduced with permission [333]. Copyright 2023, AAAS. (b) Li‐ion conductivities and activation energies for various sulfide superionic conductors, alongside Li‐ion probability density isosurfaces (dark green) for Li_6.5_Sb_0.5_Ge_0.5_S_5_I; blue and orange tetrahedra represent SbS_4_ and GeS_4_ units. Reproduced with permission [337]. Copyright 2022, American Chemical Society. (c) Refined structure of Li_2_In_1/3_Sc_1/3_Cl_4_ highlighting the blue Li4/In1/Sc1 octahedral framework. Reproduced with permission [366]. Copyright 2022, Nature Publishing Group. (d) Illustration of the 3D Li‐ion diffusion pathway formed by face‐sharing Li1 tetrahedra and Li2 octahedra (light orange). Reproduced with permission [366]. Copyright 2022, Nature Publishing Group. (e) Top view of the LaCl_3_ lattice along the *c*‐axis showing the unit cell (dashed square) and abundant intrinsic channels with an inner diameter of 4.6 Å. Reproduced with permission [367]. Copyright 2023, Nature Publishing Group. (f) Side view of vacancy‐containing LaCl_3_ lattice depicting Li^+^ migration along 1D channels (red spheres) and between adjacent channels (bidirectional arrows); vacancies are shown as grey tricapped trigonal prisms. Reproduced with permission [367]. Copyright 2023, Nature Publishing Group. (g) Typical crystalline structure of garnet‐type oxide electrolyte. Reproduced with permission [380]. Copyright 2025, Wiley‐VCH. (h) Typical crystalline structure of perovskite‐type oxide electrolyte. Reproduced with permission [380]. Copyright 2025, Wiley‐VCH. (i) Uniform Li‐metal plating on the surface of mixed ionic‐electronic conducting (MIEC) garnet due to continuous Li‐ion and electron conduction; Li metal coats pores, preventing dendrite‐induced short circuits. Reproduced with permission [23]. Copyright 2023, Nature Publishing Group. (j) Schematic of the synthesis process for free‐standing LiPON films. Reproduced with permission [400]. Copyright 2023, Nature Publishing Group. (k) Photograph of a transparent, flexible free‐standing LiPON thin film. Reproduced with permission [400]. Copyright 2023, Nature Publishing Group. (l) Relative geometries of B_12_H_12_^2−^ and CB_11_H_12_^−^ anions with boron, carbon, and hydrogen atoms shown as green, brown, and white spheres; numbers indicate Mulliken charges from first‐principles calculations. Reproduced with permission [427]. Copyright 2015, Royal Society of Chemistry. (m) Schematic of the disordered LiCB_11_H_12_ structure showing cation channels; orange and green spheres represent Li/Na and C/B atoms, respectively. Reproduced with permission [427]. Copyright 2015, Royal Society of Chemistry. (n) Arrhenius plot comparing lithium and sodium ionic conductivities in solid electrolytes; hydride electrolytes show sharp conductivity increases after phase transitions driven by complex anion reorientation, with transition temperatures lowered by lower‐symmetry anions [408]. Copyright 2016, Nature Publishing Group.

The LGPS‐type electrolyte has a unique crystal structure feature of the 1D ion transport channel along the *c*‐axis with the low ion migration energy barrier, thus displaying a high ionic conductivity [330, 331]. To further increase the ionic conductivity, more ion transport pathways are supposed to be opened. Based on that, Kanno et al. modified the elemental composition of the LGPS electrolyte and proposed the Li_9.54_Si_1.74_P_1.44_S_11.7_Cl_0.3_ sulfide electrolyte, which demonstrates not only the characteristic 1D ion pathway, but also the 2D conduction mode in the *ab* plane. As a result, the ionic conductivity has been increased to 25 mS/cm at room temperature [332]. The concept of high entropy has also been employed to design a new LGPS‐type electrolyte with higher conductivity through increasing the compositional complexity to eliminate ion migration barriers while maintaining the structural framework for superionic conduction [333]. With that, Kanno's group has developed a new LGPS‐type electrolyte, Li_9.54_[Si_0.6_Ge_0.4_]_1.74_P_1.44_S_11.1_Br_0.3_O_0.6_, with an ionic conductivity of 32 mS/cm at 25°C after hot pressing at 400°C and 370 MPa for 1 hour (Figure 12a). A solid‐state battery was thus built with a thick cathode utilizing this electrolyte as the catholyte (800 µm, LiNbO_3_‐coated LiCoO_2_, no carbon) and Li/In alloy as anode (separated by a pellet of Li_10.25_P_3_S_12.25_I_0.75_ from this electrolyte), which shows a high discharge capacity of 22.7 mAh/cm^2^ at 25°C. Although LGPS‐type electrolytes usually have high ionic conductivity, they have very poor compatibility with Li metal, leading to both dendrite growth and interface reactions. The existence of precious and high‐valence transition metal elements like Ge makes the material expensive and reducible, resulting in dramatically and continuously increased interface impedance and polarization voltage as well as low critical current density values [334].

The argyrodite‐type solid electrolyte has a formular LPSX, where X refers to the halogen anions that partially replace sulfur anions and decrease the ion transport barriers, leading to much improved ionic conductivities from 10^−5^ to 10^−2^ S/cm [335, 336, 337]. Furthermore, the ionic conductivity can be modified by changing the halogen anions [338, 339], doping with one or multiple elements [340, 341], cooling protocols [342], and external pressure [343, 344]. So far, the highest ionic conductivity of argyrodite‐type electrolyte is reported to be Li_6.5_Sb_0.5_Ge_0.5_S_5_I (16.1 mS/cm at 28°C, cold‐pressed pellet) by Yu et al. (Figure 12b) [337]. High‐entropy strategy was also utilized to design an argyrodite electrolyte with a high lithium diffusion coefficient of 1.4 × 10^−11^ m^2^/s at 29°C from PFG NMR spectroscopy, which is consistent with the experimental EIS results of 19.8 mS/cm at 25°C (after hot pressing) [338]. Despite its high ionic conductivity and relatively lower cost, argyrodite‐type electrolyte experiences dendrite formation issues with a low critical current density of 0.6 mA/cm [345]. To tackle this problem, different strategies like element doping [345], chloride content modification [346], particle microstructure control [347], artificial interlayers [348], and cell pressure optimization [349, 350] have been utilized. Further details about the improvement on critical current density and mechano‐chemical stability will be discussed in the following section.

The Li_2_S‐P_2_S_5_‐type electrolyte is also known as thio‐LISICON because their structures are replacing oxygen in LICISON‐type conductors with sulfur, bringing in higher Li^+^ ionic conductivity [351]. The unique feature of the Li_2_S‐P_2_S_5_‐type electrolyte is its mixed glass‐ceramic phase, in which the glass phase can effectively reduce grain boundary impedance and increase the compaction density and ionic conductivity. The Li_2_S‐P_2_S_5_‐type electrolyte usually has lower ionic conductivity than LGPS and argyrodite‐type electrolytes, varying around 10^−3^ S/cm. So far, Seino et al. reported the highest ionic conductivity of the Li_2_S‐P_2_S_5_‐type electrolyte (17 mS/cm at 25°C) after hot pressing at 280°C; while the cold‐pressed 70Li_2_S·30P_2_S_5_ glass and glass‐ceramic material only show 0.08 and 1.4 mS/cm, respectively [352]. Apart from heat treatment, other typical ways to improve ionic conductivity include cation doping [353] and oxygen doping [354], and halogen doping [355], which are usually also employed to improve the interfacial compatibility of the Li_2_S‐P_2_S_5_‐type electrolyte with Li metal. Sn doping was utilized by Shin et al. to improve both chemical and electrochemical stability of the Li_2_S‐P_2_S_5_ glass‐ceramics electrolyte to Li metal [356]. Lin et al. also reported that the in situ‐formed LiI within the interphase prevents continuous electrolyte degradation and limits lithium dendrite growth [355].

#### 5.1.2 Halides

Halide solid electrolytes have attracted intense interest since 2018 when Asano et al. discovered Li_3_YCl_6_ and Li_3_YBr_6_ with room‑temperature ionic conductivities of 0.51 and 1.7 mS/cm, respectively [357]. These materials offered a rare combination of high conductivity and compatibility with oxide cathodes and launched a new era of halide SE research. Halide SEs consist of monovalent halide anions (Cl^−^, Br^−^, I^−^, F^−^) and typically trivalent or higher valent metal cations. Their large halide ions give wide diffusion channels and high polarizability, resulting in low migration barriers and good mechanical deformability. In addition, halide SEs generally show a relatively high anodic stability (>4 V vs. Li/Li^+^), making them a potentially promising catholytes for high‑voltage ASSBs.

Halide SEs can be classified in multiple ways. One scheme groups them by the valence of the central metal cation: divalent (e.g., Li_2_MCl_4_), trivalent (Li_3_MX_6_ or LiMX_4_), and tetravalent (Li_2_MCl_6_) systems [358]. Another common scheme uses crystal structure and macroscopic morphology: (i) typical Li_3_MX_6_ halides, (ii) spinel‑type halides, (iii) framework‑type halides, (iv) amorphous‑type halides, and (v) soft clay‑like halides [359]. Both schemes reflect the interplay between local coordination, vacancy concentration and network connectivity. The valence‑based classification highlights how higher valent cations tend to create more vacancies and thus higher conductivities, whereas the structural classification links specific anion packing and cation ordering with transport pathways.

The Li_3_MX_6_ family (M = Sc, Y, In, rare‑earth metals; X = Cl, Br, I) derives from lithium halide structures in which trivalent metal cations partially occupy lithium sites, generating charge‑compensating vacancies. When the radius ratio of M^3+^/X^−^ lies between 0.414 and 0.732, the anion sublattice can adopt either hexagonal close‑packed (hcp) or cubic close‑packed (ccp) arrangements [360, 361]. Conductivity arises from Li^+^ hopping through octahedral and tetrahedral sites. In Li_3_YBr_6_, the three‑dimensional network of tetrahedral interstices gives higher conductivity than the quasi‑one‑dimensional channels in Li_3_YCl_6_.

Spinel‑type halides have the general formula of Li_2_MCl_4_. Their anion sublattice is cubic close‐packed, with divalent or trivalent metal cations in tetrahedral or octahedral sites [362, 363, 364]. Normal spinel halides have transition metal cations in tetrahedral sites and Li^+^ in octahedral sites; inverse spinel halides share the octahedral sites between Li^+^ and transition metal cations; and deficient spinel halides incorporate vacancies and lithium deficiency to enhance diffusion. Because divalent cations overcrowd the lattice, early spinel halides exhibited poor conductivity (<10^−5^ S/cm). In 2020, Zhou et al. synthesized Li_2_Sc_2/3_Cl_4_, a trivalent spinel, achieving 1.5 mS/cm, the first spinel halide to exceed 1 mS/cm [365]. Subsequent partial substitution of Sc^3+^ with In^3+^ (Li_2_In_1/3_Sc_1/3_Cl_4_) created additional vacancies and raised the conductivity above 2 mS/cm while enabling stable cycling at cut‑off voltages up to 4.8 V (Figure 12c,d) [366]. Spinel halides are advantageous because their relatively open frameworks can accommodate high lithium mobility; however, only a few compositions have practical conductivities and further exploration of trivalent substitutions is needed [359].

Framework‑type halides are built from open three‑dimensional networks of metal halide matrix that provide spacious migration channels. They are distinct from close‑packed Li_3_MX_6_ because the anion sublattice is not based on close packing. Examples include Li_0.388_Ta_0.238_La_0.475_Cl_3_, which shows a Li^+^ conductivity of 3.02 mS/cm at 30°C and forms a self‑passivating interphase with lithium metal, enabling more than 5000 h of cycling although the overpotential is as high as around 100 mV (Figure 12e,f) [367]. When Ta is replaced with Zr and Ca, the conductivity seems to drop by half (Li_0.495_Zr_0.259_Ca_0.086_La_0.432_Cl_3_:1.61 mS/cm at 30°C), which means the existence Ta plays an important role in the performance. However, the reduction of Ta^5+^ remains a challenge, which can be better investigated if half‐cell tests and impedance studies during cycling were carried out. Another breakthrough is LiTaOCl_4_, a mixed oxychloride with a more open framework, which achieved cold‑pressed conductivity above 10 mS/cm [368]. However, the employment of In‐Li alloy and the LYC interlayer indicates that the reduction of Ta^5+^ may still bring in interfacial issues with Li metal anodes.

Amorphous halide electrolytes bypass the crystalline requirement for specific diffusion pathways. Oxygen doping promotes amorphization; for example, 1.6Li_2_O–TaCl_5_ shows conductivities up to 6.6 m S/cm [369]. Mixed‑oxide halides such as ZrO_2_‐LiCl‐Li_2_ZrCl_4_ also exhibit high conductivities [370]. The absence of long‑range order enables isotropic ion transport and high deformability, which aids interface contact. Soft clay‑like halides such as 2LiCl‐GaF_3_ possess a layered structure with intercalated halide sheets that can shear under pressure, resulting in high ionic conductivity (3.7 mS/cm) and excellent interfacial contact with cathodes [371]. These materials blur the boundary between solid electrolytes and solid plastic crystals, offering mechanical compliance without sacrificing conductivity.

Despite the attractive bulk properties of halide SEs, their compatibility with lithium metal anodes is problematic [372]. Li_3_InCl_6_ and Li_3_YCl_6_ react with Li via reduction of M^3+^ to metallic M and formation of LiCl, leading to a mixed ionic–electronic conducting (MIEC) interphase [373]. X‑ray photoelectron spectroscopy reveals that metallic In and In_2_O_3_ signals grow with time when Li_3_InCl_6_ contacts Li, while Li 1s spectra show evolution to Li_2_O and LiOH; impedance measurements show continuously increasing interfacial resistance [372]. Similar behavior is observed for Li_3_YCl_6_ [374]. The reactions are not self‑limiting. Li^+^ and electrons diffuse through the MIEC layer, allowing the halide to decompose until either the electrolyte or Li metal is consumed [375]. The high reducibility of halide SEs arises because M^3+^ cations (e.g., In^3+^ or Y^3+^) are easily reduced to form stable halides like LiCl, LiBr or LiI; thus the thermodynamic driving force for decomposition is large [376].

To improve the lithium compatibility in the cell, one approach is to introduce interfacial modification layers. For example, coating lithium metal with β‐Li_3_N or LiCl films reduces side reactions and increases critical current density (CCD) from 0.7 to 2.0 mA cm^−^^2^ [377]. Double‐layer electrolytes can also be developed through combining halide SEs with other solid electrolytes (e.g., Li_3_InCl_6_ with Li_2_OHCl) forms stable SEI layers composed of Li_2_O and LiCl, enhancing interfacial stability and ionic conductivity [378]. Another strategy is chemical modification of the halide. Co‐doping halide solid electrolytes such as Li_2+x_Zr_1‐x_Al_x_Cl_6‐3x_F_3x_ with Al and F promotes the formation of a thin Li–Al alloy layer and fluoride‐rich (Li–F, Zr–F) interphases, which mechanically reinforce the interface, homogenize Li deposition, and suppress dendrite penetration, enabling long‐term stable cycling of Li|SE|Li symmetric cells, although the overpotential keeps increasing during the cycling process. Besides, close attention should also be paid to the doping amount, as it is shown that with higher doping, although the electronic conductivity increases, the ionic conductivity undesirably decreases [379].

#### 5.1.3 Oxides

Oxide solid electrolytes have emerged as another promising candidate for all‐solid‐state lithium metal batteries (ASSLBs) because some of them demonstrate good chemical stability and wide electrochemical windows. They are generally classified into several structural families: garnet‐type, perovskite‐type, NASICON‐type, and LISICON‐type, along with thin‐film electrolytes such as LiPON as a mature oxide‐nitride glass outside the crystalline families [380].

Garnet‐type electrolytes have the general formula A_3_B_2_(XO_4_)_3_ and a face‐centered cubic framework with multiple Li sites and can host 5–7 Li per formula unit, enabling high conductivity when lithium disorder is stabilized (Figure 12g) [380]. Li_7_La_3_Zr_2_O_12_ (LLZO) is the most studied garnet‐type electrolyte, notable for its good ionic conductivity of around 0.1 mS/cm and stability [9, 381, 382]. The cubic LLZO polymorph is the performance benchmark due to its disordered Li sublattice and three‐dimensional pathways; the tetragonal phase is less conductive. Specific dopants (e.g., Ta or Te) reliably boost the cubic phase fraction and reduce grain‐boundary resistance [383, 384, 385, 386]. LLZO is also widely viewed as one of the garnet class leaders for Li‐metal anodes because it is thermodynamically the most stable among common oxides in contact with Li metal (0.31 eV) [387]. However, the air/CO_2_ sensitivity of garnets can form resistive Li_2_CO_3_ surface layers, which increases interfacial resistance [388, 389]. Wachsman et al. demonstrated a 3D mixed ion‐electron conducting garnet architecture that delivers a super high critical current density of 100 mA/cm^2^ and enables stable Li metal cycling at ultra‐high areal capacities (up to ∼10–20 mAh/cm^2^) and current densities (∼10 mA/cm^2^) for hundreds to over 1000 h, exceeding conventional garnet SSE performance by mitigating void formation and interfacial failure (Figure 12i) [23]. This impressive performance is primarily enabled by the 3D architecture and distributed electronic pathways, rather than intrinsic material improvements. It would therefore be beneficial to determine the true local Li current density based on the actual electrochemically active surface area, rather than the geometric area, and to track how Li evolves and redistributes during cycling. It is worth noting in this current approach, scalability and manufacturability are currently constrained by the complexity of the processing.

Less compatible oxides with Li metal include perovskites, NASICON, and LISICONs frameworks, where perovskites and NASICONs have relatively high room‐temperature ionic conductivities. Perovskites with the ABO_3_ formula feature high bulk Li‐ion conductivity but suffer from grain‐boundary limitations that reduce total conductivity (Figure 12h). Li_0.34_La_0.51_TiO_2.94_ (LLTO) was first reported to have a bulk conductivity around 10^−3^ S/cm and grain boundary conductivity around 10^−5^ S/cm [390]; while Li_3/8_Sr_7/16_Ta_3/4_Zr_1/4_O_3_ shows the highest bulk and grain boundary conductivity of 0.2 and 0.13 mS/cm respectively at 30 °C [391]. So far, the major bottleneck of the ionic conductivity is still at the grain boundaries, whose conductivity is around 1–2 orders of magnitude lower than bulk [392, 393, 394]. The existence of reducible metal ions like Ta or Ti is also likely to bring in poor compatibility with the Li metal surface. This makes perovskites less suited for direct Li‐metal pairing unless interfaces are engineered. On the other hand, NASICON frameworks combine MO_6_ octahedra and PO_4_ tetrahedra in rigid networks that can host fast Li conduction with appropriate cation substitutions, especially Al^3+^ or Ti^4+^ or Ge^4+^ [380, 383]. They can reach relatively high ionic conductivities due to reduced grain boundary resistance; for example, optimized Li_1.3_Al_0.3_Ti_1.7_(PO_4_)_3_ (LATP) has been reported with conductivities approaching around 1.2 mS/cm [395], and Li_x_Al_x_Ti_2‐x_(PO_4_)_3_ (LAGP) has achieved around 0.5 m S/cm at room temperature [396]. The high ionic conductivity, combined with good air stability, makes NASICON a strong contender for practical processing, though Ti‐containing variants face challenges with Li metal interfaces [383, 397].

Unfortunately, while LISICONs show excellent high‐temperature conductivity, the room‐temperature conductivity is poor and they exhibit strong reactivity with lithium metal, which limits their applicability in ASSLMBs. For example, at 300°C, Li_14_Zn(GeO_4_)_4_ exhibits an ionic conductivity of 1.25×10^−1^ S/cm, while at room temperature it is only 10^−4^ mS/cm.

Amongst oxides, LiPON (lithium phosphorus oxynitride) stands out due to its unique mechanical properties and very limited reactivity with Li metal. LiPON is an amorphous oxide‐nitride phosphate thin‐film electrolyte fabricated primarily by physical vapor deposition. LiPON‐based solid‐state batteries are a relatively mature class of oxide‐based all‐solid‐state batteries, which are primarily produced in small batches and used in small coin cells [398]. Its strengths lie in its interfacial stability with lithium metal, chemical/electrochemical robustness, and proven reliability in micro batteries. However, scaling is impeded by deposition constraints and mechanical brittleness of ultra‐thin films. A pragmatic direction is to seek oxide glasses with improved formability and lower‐temperature processing to emulate some of LiPON's manufacturing advantages while achieving higher thickness and throughput. An illustrative example is Li_4_SiO_4_‐Li_2_SO_4_ glass and glass‐ceramics, which were prepared through mechanochemical processing followed by heat treatment at 270°C. It can exhibit improved formability and function as a secondary battery at 100°C without the need for high‐temperature sintering [399]. Meng et al. reported a novel method for creating a flexible, free‐standing lithium phosphorus oxynitride (LiPON) thin‐film electrolyte, which enables the uniform and dense deposition of lithium metal without external pressure by leveraging interfacial stress to suppress dendrite formation (Figure 12j,k) [400]. The room‐temperature conductivity remains however, extremely low (order or 10^−6^ S/cm), which limits demonstrations with higher current densities and long‐term cycling in a full cell configuration. The scalability of its complicated fabrication process may also pose challenges to practical applications. In short, LiPON is the archetype thin‐film oxide electrolyte. It excels in micro‐scale applications but faces scalability and mechanical limits for large‐format ASSLMBs.

Overall, oxide electrolytes offer several advantages, like decent chemical and electrochemical stability, thermal stability, and mechanical strength. Nevertheless, they also face significant challenges. Grain boundary resistance in polycrystalline materials often limits ionic conductivity. Approaches such as optimized sintering, doping, and fabrication of single‐crystal or glass‐ceramic electrolytes are being pursued to overcome these issues [380]. Brittleness and processing difficulties also hinder oxide electrolyte integration [383]. Interfacial resistance between oxide electrolytes and electrodes, especially lithium metal, is another critical issue. Techniques such as buffer layers, coatings, and surface modifications have shown promise in addressing this [401, 402, 403, 404, 405].

#### 5.1.4 Hydroborates

Hydroborate solid electrolytes emerged from complex hydride research originally aimed at hydrogen storage, and the modern field began with Mg(BH_4_)_2_ and LiBH_4_ [11, 406]. Mohtadi et al. demonstrated the first‐ever reversible hydride electrolyte battery. Mg deposition and stripping were demonstrated in a halide‐free inorganic salt electrolyte, Mg(BH_4_)_2_, in ether solvents, and showed that adding LiBH_4_ dramatically enhances current density and coulombic efficiency, enabling a functioning rechargeable Mg battery [11]. The implication of this foundational breakthrough was that borohydrides can serve as highly reducing, Mg‐compatible electrolyte systems, opening an entirely new class of electrolyte chemistry for practical rechargeable magnesium batteries. In their seminal work, Nakamori and Orimo et al. observed that, under microwave irradiation, LiBH_4_ underwent an orthorhombic‐to‐hexagonal phase transition near 380–390 K, revealing rapid Li‐ion transport in the high‐temperature (HT) phase at the 10^−3^ S/cm level and a conductivity jump of over three orders of magnitude versus the low‐temperature phase [406]. The same group is credited with identifying the superionic conduction in HT‐LiBH_4_ and quantifying its lowered activation energy [407]. These discoveries catalyzed the broader search across carborate anions, where superionic behavior was then demonstrated (Figure 12n) [408, 409]. Usually, hydroborate SSEs can be categorized by anion type: borohydride ([BH_4_]^−^), *closo*‐hydroborates ([B_10_H_10_]^2−^, [B_12_H_12_]^2−^), *closo*‐carbaborates ([CB_9_H_10_]^−^, [CB_11_H_12_]^−^), *arachno*‐hydroborates ([B_3_H_8_]^−^), *nido*‐hydroborates ([B_11_H_14_]^−^), and *conjuncto*‐hydroborates ([B_24_H_23_]^3−^), with mixed‐anion systems providing many of the room‐temperature (RT) leaders [410].

The archetypal borohydride is LiBH_4_, whose tetrahedral [BH_4_]^−^ anion provides four B–H bonds arranged around a boron center. LiBH_4_ exhibits a first‐order phase transition at around 390 K from an orthorhombic structure to a hexagonal phase with cooperative Li^+^ diffusion; this transition lowers the activation energy from 0.69 eV to 0.53 eV and increases Li^+^ conductivity to around 10^−3^ S/cm. Since the high‐temperature conductivity of LiBH_4_ occurs far above room temperature, numerous strategies have been explored to stabilize the superionic phase, including halide substitution [411, 412], sulfide mixing [413], and nanoconfinement [414]. LiBH_4_ provides good reductive stability toward lithium metal and can be cold pressed because of its soft, low‐density nature. However, its oxidative stability is limited. While LiBH_4_ was initially considered stable up to 5 V vs Li^+^/Li [415, 416], linear sweep voltammetry later revealed oxidative stability of only ≈ 2 V [417]. This restricts cathodes to low‐voltage materials such as TiS_2_ or Li_4_Ti_5_O_12_ [418, 419].

*Closo*‐hydroborates possess closed polyhedral anions that can rotate rapidly at elevated temperatures. High‐T superionic behavior is established, but RT conductivities of single‐anion salts are pretty low without disorder stabilization [420]. Li_2_B_10_H_10_ has a hexagonal structure at ambient conditions; Na_2_B_10_H_10_ transforms above 360 K to a cubic close‐packed phase with 0.01 S/cm conductivity at 383 K [421, 422]. Li_2_B_12_H_12_ adopts a Pa‒3 structure and undergoes a first‐order phase transition above 355°C, while Na_2_B_12_H_12_ transitions above 256°C and achieves 0.1 S/cm at 529 K [420, 423]. Anion mixing can stabilize disordered phases and yield RT superionic Na^+^ conduction [17, 424, 425].

Replacing one B–H vertex in [B_12_H_12_]^2−^ with a C–H group produces *closo*‐monocarbaborate anions. LiCB_9_H_10_ undergoes a phase transition at 332 K to a partially disordered orthorhombic phase and converts to a hexagonal phase at higher temperatures. NaCB_9_H_10_ experiences two transitions (290 K and 310 K) and retains for a period of time, a conductivity of 0.03 S/cm at 297 K due to thermal hysteresis [426]. Carbon substitution reduces the anion charge from −2 to −1, weakening cation–anion interactions. LiCB_11_H_12_ and NaCB_11_H_12_ have depressed transition temperatures (∼400 K and 380 K) and display exceptional ionic conductivities of 0.15 S/cm and 0.12 S/cm, respectively (Figure 12l,m) [427]. First‐principles calculations attribute the enhanced mobility to weaker electrostatic interactions and rapid anion rotation. Mixed carborane systems deliver the highest room temperature conductivities among hydroborate electrolytes [246, 428, 429]. A solid solution of 0.7 Li(CB_9_H_10_)–0.3 Li(CB_11_H_12_) exhibits 7 × 10^−3^ S/cm and an activation energy of 0.28 eV [429]. Its sodium analogue Na_2_(CB_9_H_10_)(CB_11_H_12_) reaches 7 × 10^−2^ S/cm at 300 K, representing the highest Na^+^ conductivity among solid electrolytes [246]. Such mixed anions stabilize the disordered phase at ambient temperature and extend the electrochemical stability window to ∼3.4 V vs Li^+^/Li [417]. Questionable qualities of the Li and Na salts prompted refined synthesis methods that minimized impurities, which was also significant in enabling a more accurate evaluation of the SSE's intrinsic electrochemical stability [240].

Other reported boron clusters type SSEs include *Arachno*‐hydroborates and *Nido*‐hydroborates. *Arachno*‐hydroborates such as [B_3_H_8_]^−^, possess open polyhedral frameworks characterized by three center bonding [410]. Alkali metal salts of [B_3_H_8_]^−^ undergo order‐disorder transitions and show moderate ionic conductivities. For example, NaB_3_H_8_ adopts an orthorhombic structure in which Na^+^ ions are tetrahedrally coordinated to four [B_3_H_8_]^−^ anions [430]. K^+^ and Na^+^ salts show distinct conduction pathways due to different coordination environments. These materials have been examined mainly for hydrogen storage rather than battery applications. *Nido*‐hydroborates, on the other hand, feature open cage clusters derived from icosahedral precursors. Examples include [B_11_H_14_]^−^, [7‐CB_10_H_13_]^−^, and [7,9‐C_2_B_9_H_12_]^−^ [431, 432, 433, 434, 435]. They exhibit order–disorder phase transitions with lower anion charge than divalent *closo*‐hydroborates, which reduces electrostatic interactions and improves cation mobility. However, their ionic conductivities are generally 2–3 orders of magnitude lower than those of *closo*‐hydroborates because high polarizability hinders ion transport. Mixed anion systems combining *nido*‐hydroborates with *closo*‐hydroborate anions can stabilize disordered phases and attain conductivities >10^−3^ S/cm, but pure *nido*‐hydroborate salts are not currently among the top performers [435].

Recently, high anodic stability was reported in *Conjuncto*‐hydroborates which are formed by linking two or more *closo*‐hydroborate clusters via oxidative deprotonation [436, 437, 438]. Sodium salts such as Na_2_B_20_H_18_, Na_4_B_20_H_18_, Na_3_B_24_H_23_ and Na_4_B_36_H_34_ were synthesized from [B_10_H_10_]^2−^ and [B_12_H_12_]^2−^ [439, 440, 441, 442]. The enlarged polyanions provide broader electrochemical stability windows (>4 V) and higher ionic conductivity than their monomeric counterparts. Although conductivity is generally lower than those of *closo*‐hydroborates, *conjuncto*‐hydroborates can be promising for high‐voltage cathodes where oxidative stability is critical.

Thus far, there has been no compelling evidence that the boron cluster SSEs can enable efficient Li metal plating/stripping, so their compatibility remains questionable. In fact, a systematic study revealed that the monocarborane has excessive reactivity with Li metal, being worse for LiCB_11_H_12_ as opposed to LiCB_9_H_10_ [240]. Similarly, poor Li plating/cycling performances were shown for Li_4_(B_10_H_10_)(B_12_H_12_) [241].

### 5.2 The Critical Role of the Anode‐Electrolyte Interface

#### 5.2.1 Formation and Properties of Native Interphases

**FIGURE 13:**
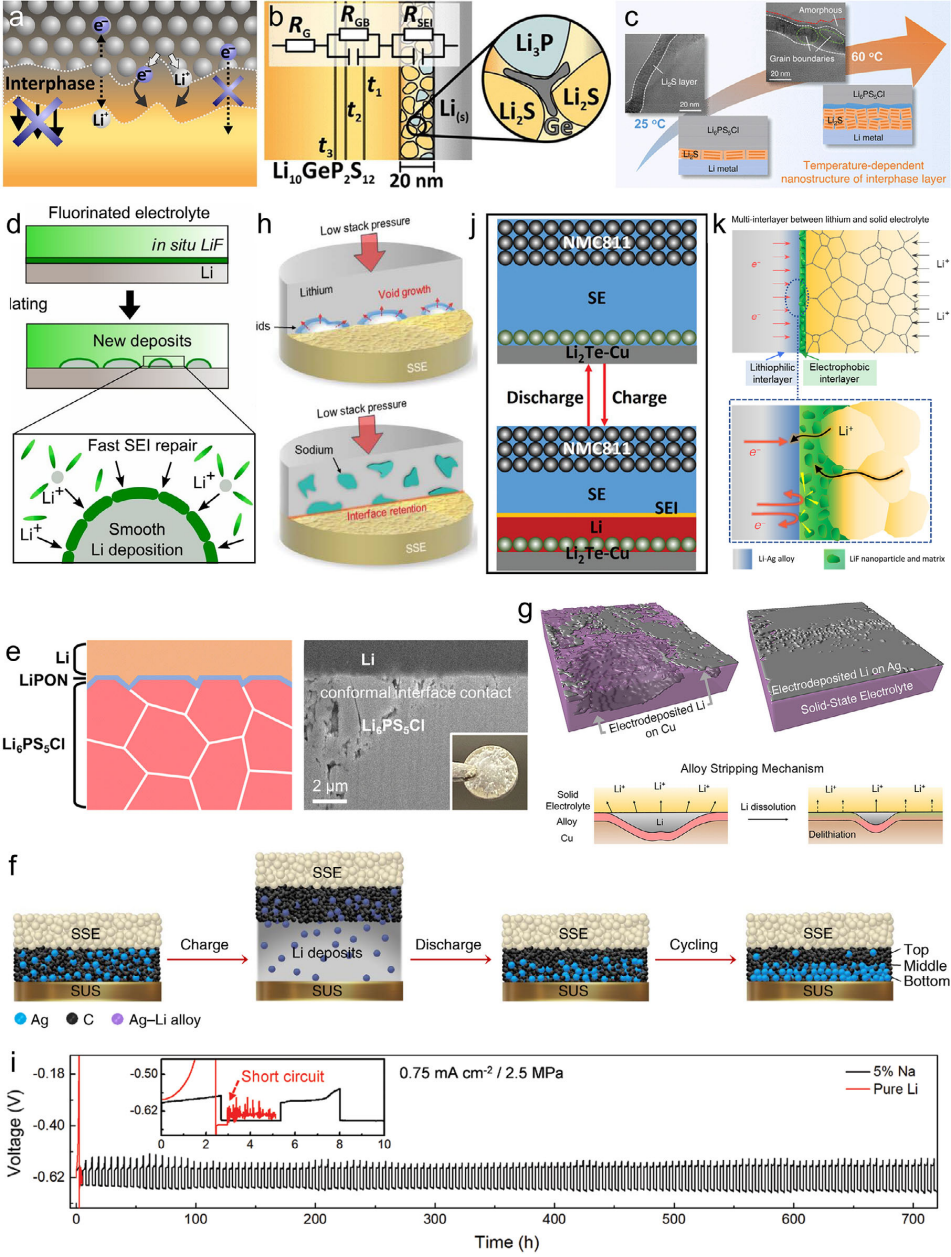
Interfaces between lithium metal anode and solid state electrolytes. (a) Ideal solid electrolyte interphase (SEI) characteristics: stable, ionically conductive, and electronically insulating. Reproduced with permission [444]. Copyright 2015, Elsevier. (b) Interphase formation schematic at the Li/Li_10_GeP_2_S_12_ interface, showing decomposition products Li_3_P, Li_2_S, and Ge (or Li_15_Ge_4_), which increase interfacial impedance. Reproduced with permission [445]. Copyright 2016, American Chemical Society. (c) Nanostructure and high‐resolution TEM images of the interphase layer between Li metal dendrites and sulfide electrolyte at various temperatures. Reproduced with permission [452]. Copyright 2022, American Chemical Society. (d) Li deposition with an in situ LiF SEI formed in fluorinated electrolyte; fluorine reacts rapidly with fresh Li deposits to create a thin, compact SEI after SEI breakdown. Reproduced with permission [151]. Copyright 2020, National Academy of Sciences. (e) Improved wetting behavior of Li metal on LiPON‐coated Li_6_PS_5_Cl SSE; FIB‐SEM cross‐section shows uniform contact, with inset photo demonstrating superior liquid Li wetting on the coated SSE. Reproduced with permission [456]. Copyright 2022, Royal Society of Chemistry. (f) Schematic of Li plating and stripping on a current collector coated with an Ag‐C nanocomposite during charge‐discharge cycles. Reproduced with permission [457]. Copyright 2020, Nature Publishing Group. (g) 3D synchrotron X‐ray microcomputed tomography renderings of the Li/LPSCl interface in cells with Ag‐coated electrodes; SSE shown in purple, electrodeposited Li in gray. Reproduced with permission [460]. Copyright 2023, Elsevier. (h) Working principle schematic of an anode‐free Li_2_Te‐Cu|SSE|NMC solid‐state battery. Reproduced with permission [462]. Copyright 2023, Wiley‐VCH. (i) Schematics illustrating Na accumulation and void mitigation during stripping of a Li‐Na alloy electrode. Reproduced with permission [463]. Copyright 2025, AAAS. (j) Long‐term cycling stability comparison of 5% Na‐doped electrodes versus pure Li electrodes under 0.75 mA/cm^2^ current density, 2 mAh/cm^2^ areal capacity, 2.5 MPa stack pressure, and Li‐In counter electrode; voltage values are negative due to Li‐In electrode potential of 0.62 V vs. Li/Li^+^. Reproduced with permission [463]. Copyright 2025, AAAS. (k) Interface schematic between lithium metal and garnet‐type solid electrolyte, highlighting interfacial design with lithiophilic and electron‐blocking interlayers; bottom panel shows an enlarged view of the interface. Reproduced with permission [465]. Copyright 2022, AAAS.

One of the unique features of solid state batteries is the solid‐solid interfaces, including both anode/SSE and cathode/SSE interfaces. An ideal interface would be thermodynamically stable and have perfect interfacial contact without interfacial heterogeneities during battery cycling (Figure 13a) [443, 444]. However, most SSEs are not thermodynamically stable with Li metal and tend to be reduced and decomposed to form interphases. The interphases can be both ionically and electronically conductive, depending on the nature of the SSE materials. For SSEs containing metallic cations in the frameworks, like LGPS or LLTO, electronically conductive interphases including Li alloy or reduced metal phases are likely to form and continuously grow [444, 445]. For example, using in situ XPS, Janek et al. observed that when paired with a lithium metal anode, Li_10_GeP_2_S_12_ was reduced into Li_3_P, Li_2_S, and Li‐Ge alloy (Figure 13b) [445], while Li_0.35_La_0.55_TiO_3_ was reduced into titanium ions and titanium metal [444]. If the reaction products between SSEs and Li metal have very low or negligible electronic conductivity, the growth of interphases will be self‐terminated after reaching a certain thickness due to the kinetic limitations. For example, argyrodite LPSX (X = Cl or Br) and LiPON can form kinetically stable interphases containing Li_2_O, LiX, Li_2_S, Li_3_P, and Li_3_N [446, 447]. Similarly, in cells using LLZO as the SSE, Li_2_O, Zr_3_O, and La_2_O_3_ are expected to form based on computational results, which have extremely low electronic conductivity [448]. Apart from the low electronic conductivity, the ionic conductivity of the interphases will also impact Li^+^ ions kinetics during electrochemical redox reactions [449]. The thickness of the interphase is also a critical factor: if it is too thin, it may promote undesired electronic conduction; conversely, if it is too thick, it can impede ion transport [450]. Generally, the composition, thickness, and microstructure of SEI can all play important roles in determining cell performance. Janek et al. reported that for Li/LPSCl, using ToF‐SIMS and AFM, they determined the Li_2_S‐rich layer thickness to be about 250 nm [451]. This interphase still has at least ∼10× higher ionic conductivity than bulk Li_2_S, likely due to the layered structure of Li_2_S‐rich layer in the SEI (Li_2_S layer next to Li metal, then P‐ and Cl‐enriched regions toward the SE). On the other hand, other studies showed that while effective passivation of Li/LPSCl (low interface resistance) occur when thin, well‐crystallized Li_2_S interphase (∼12 nm) forms at room temperature, elevated temperature (60°C) produces a thicker, polycrystalline and partially amorphous interphase (extra ∼3 nm amorphous outer layer observed) with many grain boundaries (Figure 13c) [452]. This thicker/disordered interphase leads to ultra‐high interface resistance (∼204 Ω after 120 h at 60°C) and loss of passivation. Therefore, although the formation of interphases can hardly be avoided, the ones with proper thickness, composition, microstructure, and high ionic but low electronic conductivity are desired.

#### 5.2.2 Engineering Artificial Interphases for Enhanced Stability

As the interphases play a significant role in the solid‐state battery cycling stability, many researchers have also been focusing on artificial interphases to stabilize the interfaces, improve contact, and reduce interfacial resistance, bringing in more homogenized interfacial current densities. Various Li SEI compounds, like LiF and Li_2_O, may serve as promising coatings on SSEs, inspired by the advancements made in solid electrolyte interphases (SEI) in liquid electrolytes as explained in Section 2.3 (Figure 13d) [89, 151]. It is found that Li compounds like LiF or LiPON help prevent SSE decomposition due to their low electronic conductivity, improve Li wettability, and suppress Li dendrites (Figure 13e) [453, 454, 455, 456]. Wang et al. formed an LiF‐rich solid electrolyte interphase in situ between the SSE and Li metal, which suppressed Li dendrites and could achieve a high critical current density of over 2 mA/cm^2^ [453].

Other metals or Li alloys have been another popular option for controlling the nucleation and growth of Li metal during battery cycling. Among them, silver has attracted many researchers’ interest. Lee et al. reported an Ag‐C composite anode without excess Li that can effectively regulate the plating of Li metal, leading to an excellent electrochemical cyclability in the full cell demonstration (stable average coulombic efficiency over 99.8% for 1000 cycles) (Figure 13f) [457]. Ceder et al. investigated the microscopic mechanism of the Ag/C buffer layer in anode‐free lithium‐metal solid‐state batteries, revealing that silver's ability to form a solid solution with lithium, maintain a positive lithiation potential, and undergo significant volume expansion, enables uniform lithium deposition and suppresses dendrite formation [458]. They also demonstrated that Ag‐nanoparticle fillers can help mitigate dendrite growth and reduce SSE fracture under stress intensification through unidirectional increased current density stress tests [459]. McDowell et al. found that Li–Ag and Li–Au improve Li nucleation and thus deposit a uniform Li metal layer during plating and leave less island and void formation after stripping (Figure 13g) [460]. However, it is worth noting that alloy agglomeration during cycling requires further investigations and improvements.

Besides silver, magnesium has also been utilized to form Li–Mg alloy and modify lithium transport and mechanical properties. Janek et al. showed that Li–Mg forms solid solution phases due to high Li solubility without obvious structure change, leading to higher stripping capacities [461]. However, the delithiation kinetics are limited by diffusion‐controlled lithium depletion at the interface, and repeated volume changes during cycling can degrade the alloy interphase, impacting rate capability and stability. In contrast, Mitlin et al. reported a fundamentally different mechanism in an anode‐free ASSB with the coating of Li_2_Te on Cu. The mechanism involved in situ transition of Cu_2_Te into Li_2_Te that was not delithiated during Li stripping. The lithiophilicity and electrochemical stability Li_2_Te significantly reduced the electrodeposition/electrodissolution overpotentials by around 50% and increased the CE from 98.5% to 99.7% (Figure 13h) [462].

With negligible or inexistent Li solubility to form alloys, metals like sodium Na and tungsten W have also been employed to modify electrode surfaces for Li plating. A recent approach dispersed electrochemically inactive Na metal domains into Li electrode to dynamically accumulate at the solid‐state electrolyte interface during Li stripping. The softness and creep properties of Na metal contributed to mechanically enhancing interfacial contacts, suppressing void formation, and supporting stable cycling at low stack pressures without blocking Li transport (Figure 13i,j) [463]. W interlayer works by acting as a high nucleation barrier with very low lithium solubility and high vacancy formation energy in order to suppress void formation and improve lithium dendrite growth tolerance at the solid‐state electrolyte interface [464].

To capture the different features of Li compounds and alloys, another strategy incorporated both lithiophilic and electron‐blocking layers [465, 466, 467]. Kang et al. proposed a layer‐by‐layer strategy for Ta‐doped Li_6.4_La_3_Zr_1.4_Ta_0.6_O_12_ (LLZTO) that uses metallic Ag as the lithiophilic layer and LiF as the electron‐blocking layer (Figure 13k). Due to significantly higher lithium diffusivity in Li–Ag alloy versus pristine Li metal and low electronic conductivity of LiF, a high critical current density (3.1 mA/cm^2^) and homogeneous lithium plating/stripping process was shown and attributed to the combination of a very low electron leakage and low interfacial resistances [465]. However, in practical use, achieving an appropriate balance between electron blocking and ionic conductivity is challenging, and the degradation of interlayer materials may cause morphological changes during cycling.

### 5.3 The Mechano‐Electrochemical Coupling in Solid State Batteries

Maintaining intimate contact between Li metal and the SSE is critical for durable cell cycling and, therefore, for practical application. This is governed by the interplay of complex factors, including the intrinsic mechanical properties of both the SSE and Li metal, chemical compatibility, pressing and contacting conditions, and the applied mechanical force during cycling; these aspects and related findings are discussed herein.

#### 5.3.1 Fundamental Mechanical Properties and Characterization

Besides solid‐solid interfaces, the dependence of solid‐state batteries on the intrinsic mechanical properties of the SSE, as well as those induced through processing methods used to prepare the SSE layer or incorporate it into electrodes and cells, is also unique. These mechanical properties critically influence the SSE's ability to withstand stresses, maintain interfacial contact, and suppress lithium dendrite formation during battery operation, which will be discussed in detail in this section.

One important intrinsic property of the SSE is the elastic modulus, which describes a material's resistance to elastic deformation under applied stress and can be categorized into three main types based on the nature of the stress: tensile modulus, compression modulus, and shear modulus [468]. The compression modulus measures resistance to compressive stress and is defined as the ratio of compressive stress to compressive strain; it can be derived from stress‐strain curves obtained via compression tests, often performed on cylindrical samples using universal mechanical testing machines [469, 470, 471]. Materials with a higher compression modulus usually display a lower compressibility. The tensile modulus (or tensile Young's modulus) quantifies the ratio of tensile stress to the resulting normal strain, reflecting how a material resists stretching; it is also commonly measured using a universal mechanical testing machine by analyzing stress–strain curves obtained from tensile tests on samples shaped like “dog‐bones”. Both tensile and compression moduli are closely related and, under uniaxial loading, approximate Young's modulus, representing atomic‐scale resistance to deformation. The shear modulus, on the other hand, characterizes a material's resistance to transverse deformation under shear stress and is defined as the ratio of shear stress to shear strain. It is typically measured using ultrasonic velocity techniques, where the shear wave velocity and material density are used to calculate the modulus nondestructively. These elastic moduli collectively provide critical insights into the mechanical robustness of solid‐state electrolytes (SSEs), influencing their ability to withstand mechanical stresses during battery operation, suppress lithium dendrite growth, maintain interfacial contact, and endure manufacturing processes. Advanced characterization methods such as nanoindentation and atomic force microscopy (AFM) further enable nanoscale mapping of elastic properties, revealing mechanical heterogeneities that can affect SSE performance and reliability in solid‐state batteries [472, 473, 474]. In addition to elastic deformation, materials experience plastic deformation, which is characterized by hardness, a measure of resistance to localized plastic deformation. Hardness is dependent on properties such as strength, ductility, and viscoelasticity. In most studies of solid‐state electrolytes (SSEs), hardness is determined through nanoindentation measurements.

Fracture toughness is another significant mechanical parameter in solid‐state batteries since it can help predict fast fracture of the solid electrolytes and the resultant failure of batteries. This property is highly dependent on the SSE processing and preparation methods. Higher fracture toughness usually means higher resilience to crack propagation and fracture. Nanoindentation is widely used to measure the fracture toughness of different materials, including both solid‐state electrolytes and cathode materials [475, 476, 477, 478, 479]. In this case, intrinsic parameters like elastic modulus and hardness would be measured first. For metallic materials or plastics, the notched‐specimen method is commonly used [480, 481], while the pre‐cracking method can be more accurate in measuring the fracture toughness of ceramic materials [482].

**FIGURE 14:**
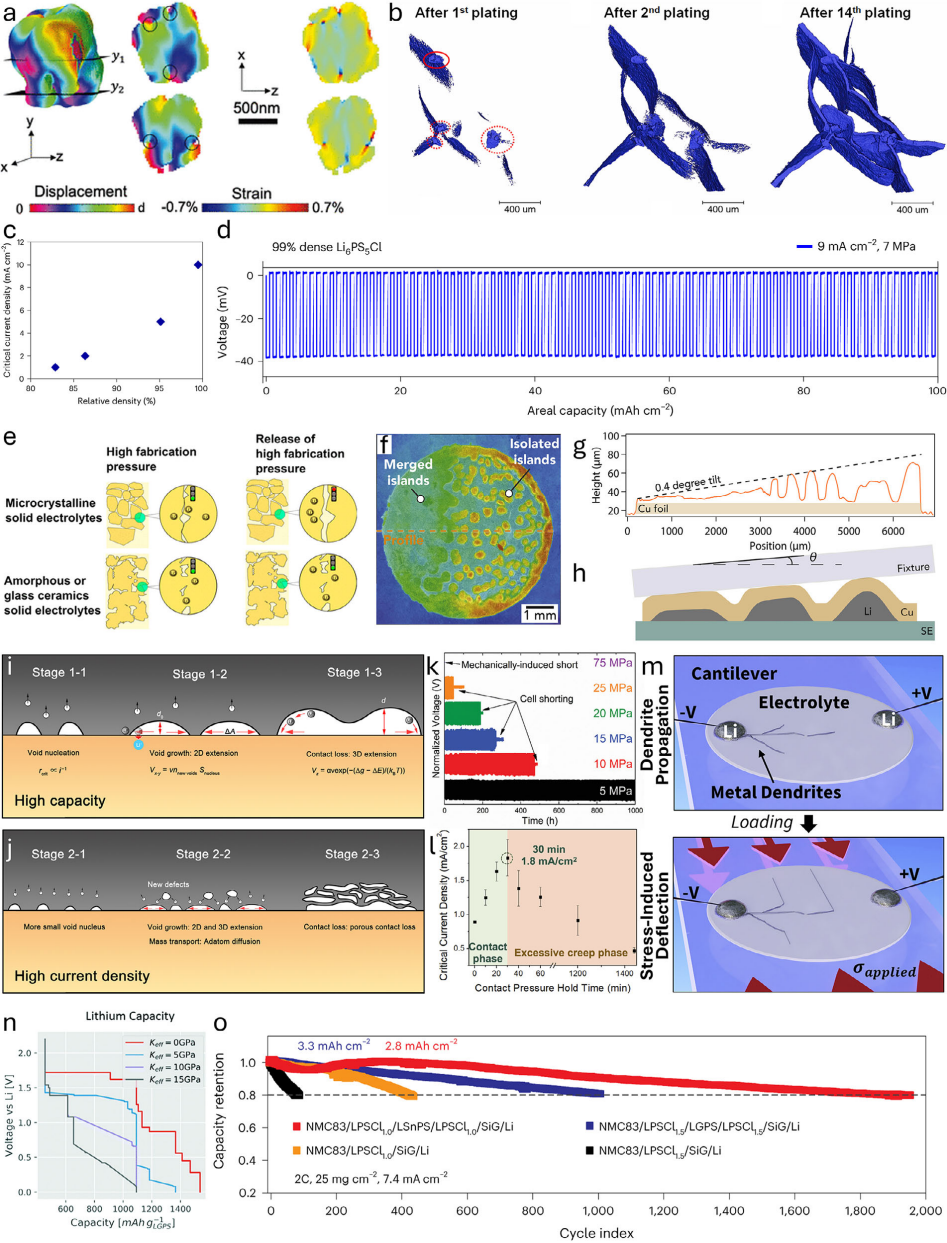
Mechanics in solid state batteries. (a) Reconstructed 3D displacement field of LLZO grains with a cubic‐tetragonal mixed structure. Reproduced with permission [485]. Copyright 2021, American Chemical Society. (b) 3D renderings of crack development on the LPS/Li surface after the 1^st^, 2^nd^, and 14^th^ Li plating cycles. Reproduced with permission [487]. Copyright 2021, Elsevier. (c) Critical current density for dendrite formation plotted against the relative density of Li_6_PS_5_Cl solid electrolytes processed under various conditions. Reproduced with permission [25]. Copyright 2025, Nature Publishing Group. (d) Cycling performance of a Li/Li_6_PS_5_Cl three‐electrode cell at 9 mA cm^−2^ plating and 0.05 mA cm^−2^ stripping currents, each with 0.5 mAh cm^−2^ capacity, under 7 MPa stack pressure using a 400 °C sintered Li_6_PS_5_Cl disk (99% dense). Reproduced with permission [25]. Copyright 2025, Nature Publishing Group. (e) Morphological differences of microcrystalline solid electrolytes depending on fabrication pressure, compared to amorphous or glass‐ceramic electrolytes. Reproduced with permission [490]. Copyright 2021, American Chemical Society. (f) 3D surface map of an electrode subjected to non‐uniform stack pressure; [505] Copyright 2022, Elsevier. (g) Height profile along the line indicated in (f). Reproduced with permission [505]. Copyright 2022, Elsevier. (h) Schematic illustrating the effect of imperfect fixture alignment on Li morphology. Reproduced with permission [505]. Copyright 2022, Elsevier. (i and j) Schematics showing microscopic Li evolution during stripping under (i) high capacity and (j) high current density conditions. Reproduced with permission [507]. Copyright 2022, AAAS. (k) Normalized voltage of Li symmetric cells over time during plating and stripping at varying stack pressures. Reproduced with permission [349]. Copyright 2020, Wiley‐VCH. (l) Trend of critical current density as a function of contact hold time at 25 MPa. Reproduced with permission [350]. Copyright 2023, Elsevier. (m) Li dendrite propagation behavior with and without applied compressive load. Reproduced with permission [484]. Copyright 2022, Elsevier. (n) Voltage profiles illustrating LGPS decomposition at different effective moduli (K_eff_). Reproduced with permission [514]. Copyright 2020, Royal Society of Chemistry. (o) Capacity retention comparison between single‐ and multi‐solid‐electrolyte‐layer batteries with SiG/Li anodes, tested at 25 mg cm^−2^ cathode loading, 7.4 mA /cm^2^ current density (2C rate), and identical total electrolyte thickness. Reproduced with permission [515]. Copyright 2024, Nature Publishing Group.

A less studied but important topic is related to internal stress generated at the atomic scale in the SSE crystallites or in the electrode components during chemomechanical synthesis and mechanical processing. These can affect not only the SSE's properties but also the growth and propagation of lithium metal dendrites [483, 484]. Bragg coherent diffractive imaging (BCDI) is one appropriate method to measure the crystal internal stress and reconstruct the 3D field of atomic displacement inside solid‐state electrolyte grains. For example, Sun et al. used BCDI to reveal edge dislocations, twin domain boundaries, and pronounced strain inhomogeneity in LLZO, showing mixed‐phase (low‐Al) grains exhibit higher strain energy and extended tensile regions compared with largely strain‐free cubic grains (Figure 14a) [485]. The mechanical force conditions inside the SSE can also be clearly observed via strain distribution, which can be qualitatively displayed by X‐ray computed tomography (CT) in 3D (Figure 14b) [486, 487]. The visualization of larger stress and strain inside the solid electrolyte during crack and dendrite propagation can guide advanced materials and engineering design to improve cell performance in practical applications.

#### 5.3.2 The Dual Role of External Pressure: Fabrication and Stack

Apart from internal pressure, external pressure also plays a significant role in cell performance, from manufacturing to cycling [344]. Fabrication pressure refers to the pressure applied during the manufacturing processes of both electrolyte and electrodes, including pellet pressing and SSE, electrode calendaring. Two methods can be utilized to apply fabrication pressure: uniaxial and isostatic pressing, among which isostatic pressing is likely to reach higher density due to more uniform distribution of pressure [488]. The other important type of external pressure is stack pressure, which holds the cell components together during cycling. The optimal values for both fabrication and stack pressure are dependent on the type of solid electrolytes. Halide SSEs usually need much higher fabrication pressure than sulfide and oxide SSEs (200–400 MPa vs. <200 MPa) [489]. However, lower stack pressure is more desirable to fragile oxide SSEs (<10 MPa) compared to sulfide and halide SSEs (10–400 MPa). Oxide SSEs usually need high‐temperature sintering to realize the best performance.

#### 5.3.3 Mechanical Effects on the Solid State Electrolyte

One of the most direct influences of fabrication pressure on the solid electrolyte is the pellet density and porosity, which further affects the ionic conductivity. Different types of SSE may behave differently under various fabrication pressures. Microcrystalline Li_6_PS_5_Cl pellet pressed under higher fabrication pressure (370 MPa) shows a larger grain size, higher density, and lower porosity than lower pressure (50 MPa) [344]. This is because higher fabrication pressure brings in a more ordered and denser internal structure, which facilitates Li^+^ ions movement and thus increases ionic conductivity (2.28 vs. 0.99 mS/cm). Bruce et al. demonstrated that high plating currents of up to 9 mA/cm^2^ can be achieved without dendrite formation by sufficiently densifying argyrodite (LPSCl) to 99% and used modeling to show that changes in microstructure (smaller pores, shorter cracks) are responsible for this increase in critical current density (Figure 14c,d) [25]. However, it seems that a very low stripping current of 0.05 mA/cm^2^ is needed and whether this principle is applicable to other SSE (e.g., garnets) is yet to be evaluated. For other sulfide SSEs, crystallinity also affects their dependence on fabrication pressure differently [490]. The ionic conductivity in amorphous (e.g., Li_2_S‐P_2_S_5_) and partially crystallized glass ceramic (Li_7_P_3_S_11_) solid electrolytes continuously increases with the fabrication pressure between 100 and 400 MPa attributed to pressure‐induced sintering of amorphous particles and irreversible densification of pellets (Figure 14e). However, due to the brittleness and limited plastic deformation, increasing fabrication pressure is not an efficient approach to increase the pellet density and ionic conductivity of oxide SSEs [380, 491, 492]. Therefore, calcination and sintering steps are typically utilized to improve the crystallinity of solid state electrolytes and form better grain boundaries to increase the ionic conductivity [384, 493].

Stack pressure may also influence the ionic conductivity of solid state electrolytes, especially the ones with microcrystalline structures. This is because gaps and voids inside the electrolytes regenerate after removing the fabrication pressure and the stack pressure is usually much lower compared to the fabrication pressure. As a result, the apparent conductivity is likely to decrease with lower stack pressure. For example, at low stack pressures below approximately 30–50 MPa, the measured ionic conductivities for sulfide‐based solid electrolytes, including Li_10_GeP_2_S_12_, Li_6_PS_5_Cl, Li_6_PS_5_Br, and Li_7_P_3_S_11_ are very low due to poor and non‐reproducible contact between the solid electrolyte pellet and the tungsten carbide electrodes, an issue that is largely resolved when using soft carbon powder as the current collector instead [490]. Generally, solid state electrolytes with different mechanical properties have different dependence on the stack pressure. Compared to oxide electrolytes, sulfide and halide electrolytes with lower elastic modulus usually demonstrate more significant increases in ionic conductivity when the stack pressure is increased in the range of 0–30 MPa because of the improved contact [489]. The contact between cathode and electrolytes is also essential to the cell performance, which may be influenced by fabrication pressure because it can reduce the porosity of the composite cathode and thus improve the contact between electrolyte and cathode particles, leading to better electron and ion transport [494, 495]. Stack pressure, on the other hand, affects the contact during charging and discharging process through mitigating volumetric expansion and shrinkage [496, 497, 498, 499]. Increasing stack pressure to 10 MPa was shown to improve capacity retention by maintaining intimate particle contact and cathode microstructure [499].

#### 5.3.4 Mechanical Effects on Lithium Metal Anode

Lithium metal plating behavior was found to depend on the cell pressure. During plating, the defects on the surface of current collectors can lead to uneven pressure and current density distribution, which causes non‐uniform nucleation of Li metal. In this case, Li tends to grow on these active sites, further increasing the local stress. As the pressure builds up, the SSE becomes prone to cracking causing Li metal dendrites penetration into the SSE [489, 500]. According to linear elasticity theory, local mechanical stresses and surface tension at the lithium/electrolyte interface influence the kinetics of lithium plating by altering the electrochemical potential and reaction rates. This understanding highlights the importance of designing electrolytes with sufficient mechanical strength to suppress interface roughening and ensuring uniform charge and mass transport for stable electrodeposition [501, 502, 503, 504].

Due to the impact of local pressure on both nucleation and kinetics of lithium plating, the morphology of deposited Li also depends on the cell pressure. In fact, without external pressure, non‐uniform plating of Li on a planar surface may lead to the formation and growth of isolated Li metal blisters. Dasgupta et al. reported that, under external pressure, these blisters merge to promote lateral growth on the current collector, bringing in a flatter morphology and suppressing Li dendrites (Figure 14f‐h) [505]. To realize this effect, the pressure applied needs to fall within a certain range, for example, while too low pressure may be ineffective, too high pressure that leads to exceeding the yield strength of Li metal would cause Li metal irreversible plastic deformation and creeping [349, 506].

It is important to note that Li creeping phenomenon is not necessarily always undesirable. Repeated plating/stripping cycles are likely to induce the formation of interfacial voids, leading to morphological instabilities and thus cell failure. In fact, Li creeping originating from the application of an optimal external pressure can hinder the formation of voids through reducing the Gibbs free energy of voids nucleation (Figure 14i,j) [507]. Besides, Li creeping can also promote the transport of vacancies and decrease the volume of voids or pores, improving the interfacial contacts and cell stability [489]. Importantly, the inactive lithium without ionic contact on the current collector can be reconnected and reused under the application of proper external pressure [508].

Li creeping behavior can be controlled by parameters like pressure strength and time [349, 350, 509, 510]. For example, Meng et al. reported that in Li_6_PS_5_Cl, a moderate stack pressure in the range of 5–25 MPa brings in less dendrite formation and better interfacial contact, while a high pressure of 75 MPa immediately leads to dendrite formation due to stress concentrating at tips and thus causes the cell to short (Figure 14k) [349, 484]. In addition, the process of pressure application during compressing the Li metal against the SSE also plays a role. For example, an optimal pressure holding time of 30 min at 25 MPa was necessary to reach the highest critical current density of Li_6_PS_5_Cl (1.8 mA/cm^2^, Figure 14l) [350]. Therefore, consideration and effective control of both external pressure and its application process during cell fabrication can significantly contribute to optimizing the cell performance.

Beyond its impact on Li creeping behavior, external pressure may influence Li metal crystal structure or dendrites trajectory during Li plating [484, 511, 512]. Consideration of this is helpful for understanding Li metal behavior. For example, regarding the crystal structure, various Li phases have been observed under high pressure and low temperature range [511], or the report of what is believed to be glassy Li metal in liquid electrolytes systems [512].

The impact of pressure on Li dendrites propagation goes beyond its magnitude and extends to its loading axes. In an interesting study, Chiang et al. found that the external pressure superimposes a compressive stress that deflects the dendrite growth trajectory toward the loading axis. When compressive loads (≥ 150 MPa) were applied horizontally across the SSE (Figure 14m), dendrite penetration was redirected to grow parallel to the compressive stress axis, which prevented the occurrence of a short‐circuit [484]. We, however, note that dendrite penetration through the electrolyte is undesired regardless of their growth vector. Such dendrites compromise SSEs' mechanical integrity, ultimately leading to cell failure.

Importantly, recent findings reveal that dendrite growth in solid electrolytes is not driven solely by mechanical stresses but is significantly accompanied by electrochemical corrosion processes at the dendrite tips [513]. Using operando microscopy and stress measurements in garnet Li_6.6_La_3_Zr_1.6_Ta_0.4_O_12_ solid electrolytes, it was shown that plating‐induced stresses coexist with electrochemical degradation, which weakens the electrolyte and facilitates dendrite propagation. This coupling reduces the mechanical stress required for dendrite growth by up to 75% compared to mechanical stress alone. Therefore, controlling both mechanical and electrochemical factors is critical for improving the cycling performance and safety of solid‐state batteries.

#### 5.3.5 Interfacial Mechanics: Contact and Reaction Kinetics

The interface between SSE and Li metal anode represents not only the point of physical contact but also where chemical/electrochemical decomposition reactions take place. As mentioned before, interphases are prone to form due to thermodynamic instability of SSEs when contacting Li metal. However, the kinetics of these process were proposed be impacted by applied external pressure in what was described as mechanical constriction [514, 515]. Li and co‐workers reported this phenomenon via the application of MPa‑level external pressure amplified through a dense graphite interlayer in the Li‐LGPS system (Figure 14n), or via lithiation‑induced strain localized around micrometer Si particles (Figure 14o), which can impose GPa‑level constraint that kinetically suppresses deep reduction of the electrolyte. This localized mechanical barrier confined Li alloying or SSE decomposition to thin surface shells by imposing a high mechanical constriction that limits volumetric expansion and reaction front propagation. As a result, Li deposition is redirected into residual interparticle voids within the electrode structure, which promotes more uniform plating and effectively inhibits dendrite growth by preventing localized stress accumulation and uneven lithium flux at the interface.

## 6 Summary and Outlook

### 6.1 Advancing Metal‐Anode Batteries: Unifying Principles

**FIGURE 15:**
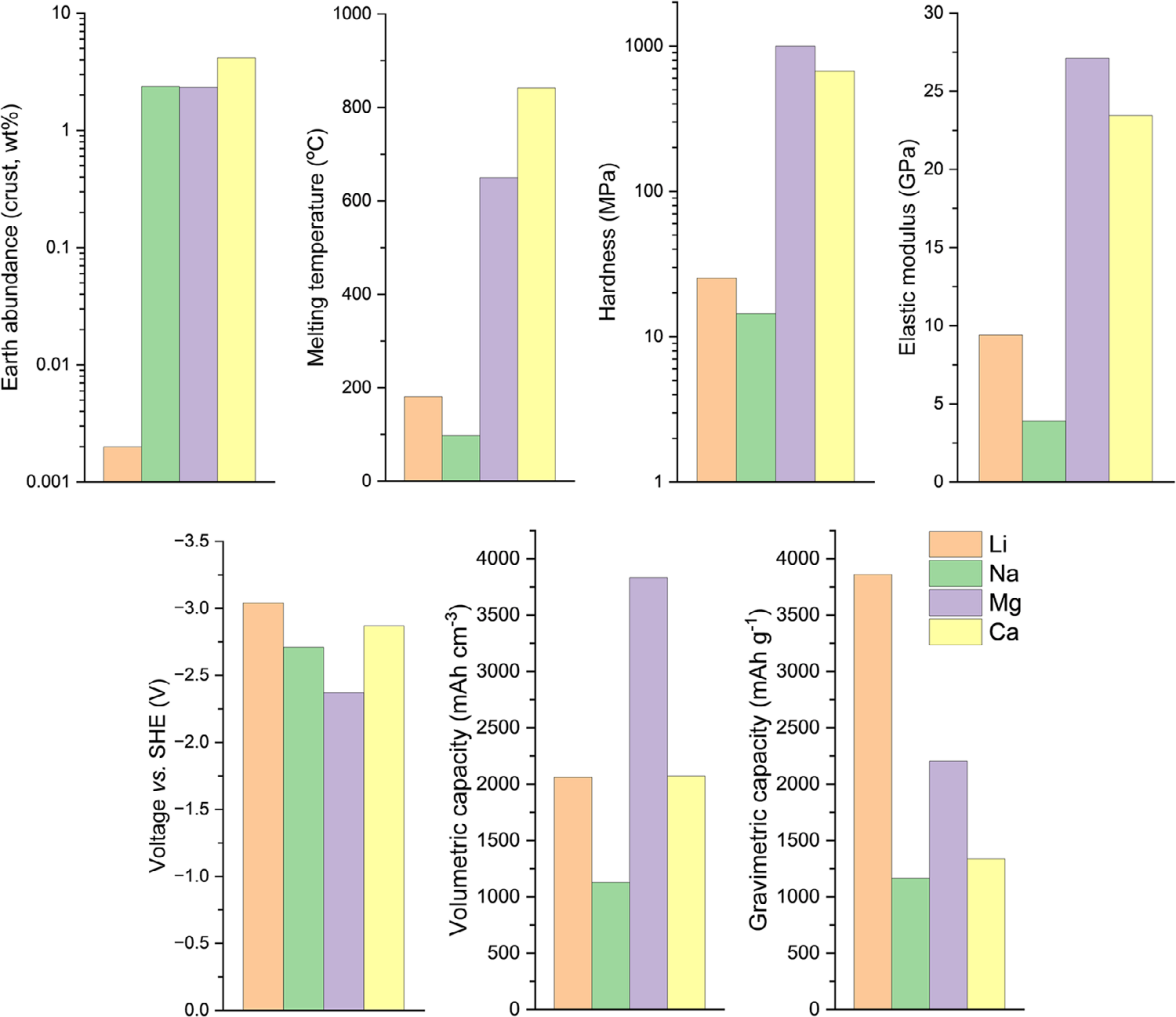
Comparison of different metal‐anodes using key metrics of relevance to practical use. Elastic modulus and hardness are obtained from ref. [305, 306, 516, 517], where average values of hardness for Li, Na, Mg, and Ca are shown.

**FIGURE 16:**
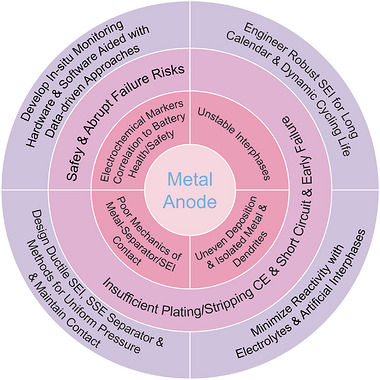
Common challenges and potential directions for the future development of metal‐anode batteries.

In this review, we have provided a comparative assessment of the four representative metal‐anodes, highlighting their differences in key practical metrics (Figure 15). This multi‐dimensional comparison illustrates the unique strengths and trade‐offs associated with each chemistry and emphasizes that no single metric alone determines commercial viability. We have also distilled the guiding principles that have advanced room‐temperature reversible metal‐anode batteries and identified the barriers currently preventing their commercial viability. An important milestone is showing, in principle, that relatively high metal plating/stripping Coulombic efficiencies can be achieved: 99.8‐99.9% for Li and Na, in both liquid and solid electrolytes, and for Mg in liquid electrolytes, and 95% for Ca in liquid electrolytes. However, these values are typically demonstrated only under a limited set of plating/stripping conditions in research laboratories that may not reflect practical operating conditions, and premature cell failure still occurs because of complex underlying fundamental processes. Note that Coulombic efficiencies exceeding 99.9% are required for practical use. The metal‐anode systems share common challenges across chemistries and to address these limitations, integrated strategies are needed (Figure 16). Our goal is to accelerate next‐generation technologies that can reliably demonstrate compelling value beyond current Li‐ion performance, providing advantages strong enough to justify widespread adoption. We emphasize that these technologies are intrinsically linked; recognizing the commonalities between monovalent and multivalent systems allows for a more unified approach to electrolyte and interface engineering. With a central theme focused on electrolyte studies and their naturally occurring interphases, we demonstrate that advancements in multivalent metal systems were significantly informed by foundational progress in Li and Na metal technologies.

For liquid electrolytes, the core design principles are shared across metal anode batteries, centering on the maximization of cation coordination to a ligand bound to an anion or an additive solvent. The objective is to engineer a stable interphase that facilitates metal deposition with appropriate morphological features and chemical composition. However, the specific chemical requirements diverge significantly by chemistry. For lithium (Li) and sodium (Na), the common pursuit is the formation of fluorine‐rich interphases, a strategy that has largely contributed to an impressive increase in Coulombic Efficiency (CE) over the last decade, reaching, respectively, as high as 99.8% and 99.9%, as measured in half cells. Conversely, Magnesium (Mg) batteries necessitate the minimization of fluorine content to prevent passivation, with most robust designs opting for organic‐dominant solvation shells to produce interphases with little to no fluorine or other inorganic content.

In the realm of Solid‐State Electrolytes (SSE), the linchpin of their performance is Li metal cells is the formation of kinetically stabilized interphases produced via the decomposition of the SSE, although artificial interphases may also represent a viable approach. Prominent examples of competent electrolytes include sulfides for Li‐metal batteries and hydrides for Na‐ion batteries, with hydrides being proposed and demonstrated to have high compatibility with reactive metals over a decade ago [11]. However, we caution against claims about the viability of intrinsically incompatible electrolyte families (e.g., halides family) based on limited testing and cycling data. Such systems often exhibit high and frequently rising overpotentials as passivating SEI layers progressively builds up with cycling. These effects may be further intensified when metallic species within the electrolyte are reduced; if electronically conductive, they can catalyze additional electrolyte decomposition. Furthermore, failure modes in SSEs are distinct from liquid systems; they are not solely chemical/electrochemical but often involve underlying chemomechanical factors that are absent in liquid electrolytes. For example, the mechanical properties profile of the SSE is an important contributing factor to cell performance.

A common issue in metal anode systems is the lack of standardized and consistent reporting regarding cell hardware components, such as separator/SSE and spacer thicknesses, spring, and testing conditions like preconditioning. These factors significantly affect cell performance metrics, including Coulombic efficiency CE, cycle stability, and morphological changes. Additionally, CE measurements in half‐cells (e.g., inert working electrode/metal reference and counter electrodes) are often reported without considering activation effects, i.e. capacity lost in early cycling due to SEI formation. For instance, relying solely on the modified Aurbach method for CE measurement which involves initially passing large capacities, can obscure the influence of parasitic reactions occurring during early plating/stripping cycles. Therefore, detailed reporting of cell components is essential, and CE should be measured using both the Aurbach method and cycling methods as described in Section 2 to accurately assess the electrolyte's performance.

**FIGURE 17:**
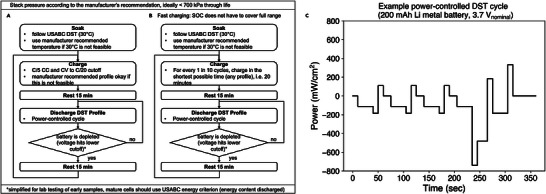
Proposed battery life testing protocols using dynamic stress test (DST). Copyright 2024, Elsevier [519].

The disconnect between academic testing protocols and the performance requirements of real‐world battery applications remains another significant barrier to progress. Published test protocols are often far removed from the conditions encountered in commercial cells, making it difficult to assess how the reported performance of new materials will translate into practical devices. We propose that, in addition to standard screening methods (half‐cell CE measurements, symmetric cell cycling and basic rate studies), the field should progressively consider the adaptation of industry‐relevant testing conditions based on established benchmarks, for example, in the USA, those are defined by the USABC [518]. Recently, leading industrial and academic scientists and engineers proposed battery life testing protocols using dynamic stress test (DST) to probe the battery operation under dynamic conditions that mimic the real operation of the vehicle (Figure 17) [519]. Other tests include calendar life testing such as specific reference performance tests (RPTs) which would be most informative when conducted under a set of pre‐defined conditions that can be established by the field. It is worth noting that these tests are conducted using a standardized full‐cell pouch size with selected limited metal excess and lean electrolyte loading (for example, E/C ≤ 5 g/Ah) whilst passing realistic areal capacity, i.e. at least 3–5 mAh/cm^2^. While these tests may not be appropriate for early‐stage research, the development of modified protocols that give related information from the very beginning would be highly effective in gauging the practical relevance of a given advance. Ultimately, only through performance evaluation under conditions that approximate real‐world demands can we determine whether a promising electrolyte formulation or interphase design is poised to survive the transition from laboratory to product.

### 6.2 Proposed Research Directions

#### 6.2.1 Liquid Electrolytes‐Based Batteries

##### 6.2.1.1 Monovalent Metal Anode

Li and Na metal anodes may offer compelling “drop‐in” opportunities to boost cell‐level battery energy density while leveraging much of the existing Li‐ion manufacturing infrastructure. They also face many of the same fundamental challenges, although their severity differs. For example, the higher solubility of Na organometallic species can make it harder to achieve durable long‐term performance. In contrast, Na metal systems have reached coulombic efficiencies (CE) as high as 99.9% across a range of electrolytes, performance that has not yet been demonstrated for Li metal. Overall, electrolyte design concepts developed for Li metal have translated well to Na metal, improving compatibility for reversible plating/stripping. Despite the wide variety of reported formulations, many rely on the same central principle: the use of highly fluorinated electrolytes.

However, this approach has not yet resolved key challenges associated with metal anode. In LMB systems, reliance on LiFSI to promote LiF‐rich SEI introduces important tradeoffs as this often requires reducing solvent participation in the solvation shell (as in HCEs and localized HCEs), which can limit rate capability due to reduced ionic conductivity. In addition, achieving the highest CE frequently requires extended conditioning over many cycles, leading to metal inventory losses on the order of 5–10%, a serious drawback for anode‐free configurations. Under lean conditions, abrupt failure becomes difficult to avoid. On the other hand, in Na metal batteries, high CEs% are achievable in a variety of salts, including dilute NaPF_6_‐based electrolytes. However, the benefits of forming NaF‐rich SEI to stabilize the overall performance can be offset by the relatively insulating character of NaF‐rich interphases, as opposed to LiF.

Both Li and Na metal heavily rely on ether‐based electrolytes, and both can suffer from gas evolution and are prone to corrosion during rest (often more severe for Na), creating a significant practical barrier. Finally, while fluorinated solvents may contribute to fluorinated SEI formation and improve anodic stability with oxide cathodes, the outgassing issue can be more severe, and many fall under PFAS‐related scrutiny, raising concerns for commercialization and sustainability. Additional challenges associated with salts like those imide‐based include corrosion of aluminum current collectors and as demonstrated in the case of FSI^−^ stringent purity requirements to mitigate it, increasing electrolyte manufacturing complexity and cost.

A less addressed concern is the longevity and stability of the SEI, which is tied to its mechanical and chemical stability so new frontiers should focus on the design of solvents and additives capable of supporting stable performance. The large volume changes during cycling necessitate consideration of the mechanical robustness of the SEI as a crucial factor in protecting the interphase that forms. Lessons learned from lithium‐ion systems indicate that even volume changes limited to less than 10% require careful selection of solvents and solvent additives to maintain a robust SEI throughout expansion and contraction cycles. Inorganic SEI components tend to be more brittle, while organic components are softer and more flexible. The availability of new solvents and organic additives may open opportunities to re‐explore salt choices and inspire novel designs, thereby introducing new possibilities for effective electrolytes. Other options may include screening the effects of the organic solvent through utilization of porous liquid or solids, as discussed in Section 2. In this case, it is necessary to establish the compatibility of these components with Li/Na metals and assess the impact of cation‐host association on cell overpotentials.

Guidance can be gained by placing greater emphasis on understanding the mechanisms, structure, and evolution of SEI formation, which remain insufficiently understood. This is a challenging task given that the SEI is nanoscale, unstable, and composed of a complex mixture of inorganic and organic species, with its composition dependent on formation and cycling conditions such as temperature, current density, and cycling protocols. However, the combination of advanced experimental techniques with artificial intelligence (AI) and machine learning (ML) can hold great potential to uncover complex patterns and relationships that are difficult to identify using traditional methods.

As electrolyte formulation designs are considered, it is also beneficial to objectively examine scalability bottlenecks and safety concerns. For instance, highly volatile or flammable systems may offer desirable performance but could pose significant challenges for practical implementation, including manufacturability.

##### 6.2.1.2 Multivalent Metal Anode

Over the last decade, magnesium metal anode research has witnessed tremendous progress. The field has evolved from a binary search for Mg metal compatible electrolytes to a broader scope that now includes the exploration of interphases and deposition strategies designed to foster uniform growth and extend cyclability. While these developments are academically compelling, translating them into a functional battery demands a more critical assessment. Therefore, we review here these results not merely as scientific achievements, but through the pragmatic lens required to transition from laboratory success to industrial reality.

The increasing reports of high‐performing electrolytes, with Mg metal cycling efficiencies exceeding 99% [26, 271, 276, 277, 279, 292, 520, 521, 522], represent a milestone of maturity for the field, but these performance metrics must be reviewed critically. On the one hand, from an industrial perspective, a CE of 99% is merely the baseline for entry; to compete in the electric vehicle or grid‐storage markets, the CE must consistently exceed 99.9% (ideally 99.95%) to match current Li‐ion battery cycling life. On the other hand, some of the cell testing is performed using thick glass fiber separators or with multiple separators that allow for increased cell cycling even under non‐uniform Mg metal growth. To convince industrial partners to take Mg metal cells to the next level, electrolyte performance must be demonstrated free from engineering artifacts by transitioning toward standardized testing involving commercially relevant separators.

The discovery of “SEI‐like” layers on Mg metal is arguably one of the most significant recent advancements. It offers the potential for simplified manufacturing of commercial cells and has democratized the field by allowing a broader range of researchers to use commercially available salts like Mg(TFSI)_2_ and Mg(OTf)_2_. However, despite the large new design space a functional interfacial layer theoretically opens, the reported systems remain tethered primarily to ether‐based formulations that inherently cap oxidative stability [260]. True industrial viability hinges on breaking this chemical confinement to unlock solvent systems capable of supporting the high‐voltage cathodes necessary for competitive cells.

We caution, however, against falling into the trap of considering the SEI as a universal solution. These layers can act as significant kinetic bottlenecks, potentially sacrificing the high power density that makes Mg so attractive. This is where Mg metal offers a unique opportunity that sets it apart from sodium and lithium: the “near‐free‐interphase” regime. Mg metal appears capable of forming absorption layers at rest that protect the metal from corrosion while remaining permeable to plating and stripping upon voltage demand [26, 288, 289, 290]. This suggests a pathway to achieving high energy density without the resistive penalties of a permanent solid layer. Future efforts should be also directed towards exploiting these intrinsic surface protection mechanisms of Mg metal.

In the broader context of energy storage, it is unfortunate that Mg metal is still regarded by much of the battery community as a primarily academic exercise, as evidenced by the significantly larger volume of research on Li‐metal anodes. However, as the advances summarized in this review demonstrate, this perception is not due to any fundamental limitation of the Mg metal itself. These developments represent solid steps on a path toward practical application, yet significant work is required to elevate magnesium metal to a commercially competitive level.

#### 6.2.2 Solid State Batteries

Solid‐state electrolytes (SSEs) offer a pathway to addressing many of the challenges metal anodes face in liquid electrolytes. Replacing organic solvent‐based systems with inorganic SSEs can mitigate problems related to SEI solubility and the formation of unstable organic interphases. To date, SSE research has largely emphasized improving bulk ionic conductivity; however, enabling reliable metal‐anode batteries demands a shift in priorities toward rigorous design and control of the dynamic chemo‐mechanical interface. Progress will require a holistic approach that integrates new materials design, AI‐enabled discovery, standardized benchmarking, and practical engineering.

##### 6.2.2.1 Overcoming Challenges Through SSE Design

High metal plating/stripping coulombic efficiency (CE) approaching 99.9% has been demonstrated in inorganic SSEs, but limited areal capacity, poor durability, and the need for high stack pressure remain major barriers. Many studies rely on thick electrolyte pellets (>500 µm) that are not industrially relevant, or on excess metal reservoirs that mask true electrolyte performance, often turning “improved cycling” into little more than delayed failure.

To date, SSE design has frequently faced a trade‐off between ionic conductivity and electrochemical stability, leaving few practical options. Sulfides are among the leading candidates for Li metal, while Na metal tends to cycle best with *closo*‐borates; however, neither system, at least in their room‐temperature high‐conductivity forms, offers robust stability against 4 V‐class cathodes. In the multivalent systems, there are no available room‐temperature superconductors, i.e. conductivity > 10^−4^ S/cm so inorganic all‐solid‐state electrolyte development targets improving the cationic conductivity. It should be, however, noted that the high hardness of these metals, as opposed to Li and Na, may introduce challenges associated with poor contacts leading to high interfacial resistances; this makes the use of solvents, plasticizers or even artificial SEIs potentially unavoidable.

From the standpoint of metal‐anode compatibility, future materials discovery should prioritize intrinsic stability in strongly reducing environments. This includes identifying electrolytes that are either more thermodynamically stable against the metal or that form a truly self‐limiting, highly conductive, and mechanically robust interphase. Machine learning may help accelerate this search by leveraging existing knowledge of SSE interfacial behavior and extending predictions beyond bulk conductivity to include reduction potential, adhesion energy, and key mechanical properties (e.g., shear and bulk modulus). In parallel, AI can support microstructural optimization by linking fabrication conditions to performance, enabling SSE manufacturing with reduced tortuosity and fewer defects.

Whereas liquid‐electrolyte design for metal anodes typically emphasizes chemical and electrochemical stability, solid‐state electrolyte (SSE) development must also account for mechanical behavior. SSE cracking and void formation at the metal interface are key failure drivers, indicating that more ductile electrolytes could help suppress these degradation pathways. While high stack pressure in laboratory tests can reduce voiding and delay dendrite growth, it is not scalable because it increases cost and reduces energy density. The target is therefore stable metal cycling under low external pressure. To enable objective assessment, early‐stage studies should explicitly define an SSE's operating electrochemical and mechanochemical boundaries, capturing both strengths and limitations.

Beyond interfacial stability, chemical robustness and processability deserve greater attention as practical descriptors. Relevant questions include moisture tolerance, handleability (e.g., pellet/film brittleness), and metal wettability. For instance, sulfide and halide electrolytes are generally moisture‐sensitive, with halides often being particularly corrosive. Oxide electrolytes are typically less reactive to moisture but can suffer from interfacial impedance growth, poor physical contact with both anode and cathode, and localized reduction reactions that degrade performance. Hydride electrolytes, in many current implementations, still face challenges with long‐term interface stability against lithium metal, and their complex chemistries introduce additional design constraints. Overcoming these limitations is critical to advancing safe, durable solid‐state battery technologies.

A distinctive challenge for solid‐state electrolytes (SSEs) is that translating academic advances into practical devices requires explicit consideration of the mechanical boundary conditions needed for stable metal operation, factors that are often omitted from fundamental studies but deserve attention in this early phase. For assessing commercial viability, thin‐film SSEs are particularly relevant: electrolyte thickness should be minimized (<50 µm) while areal capacity per cycle is increased (>4 mAh/cm^2^), so the SSE functions primarily as a separator rather than a resistive bulk layer.

Accordingly, it is critical to develop scalable methods to fabricate free‐standing, thin‐film SSEs that retain mechanical integrity. This effort is constrained by the limited availability of binders, including those suitable for dry processing, that remain compatible with reactive metals such as Li and Na. For example, PTFE, a common binder for dry processing, can be incompatible with lithium metal, complicating adoption of this approach.

Elucidating the complex failure mechanisms at the metal/SSE interface is essential for designing metal‐compatible solid‐state electrolytes, but it will require wider adoption of advanced *operando* diagnostics. Techniques such as X‐ray computed tomography (X‐ray CT) and *operando* neutron imaging can capture the 3D, time‐resolved evolution of interfacial contact loss (voiding) and dendrite formation during cycling, enabling quantification of “dead metal” and direct correlation with capacity fade. Because mechanical stress influences both dendrite penetration and void formation, future studies should also track in situ stress and the evolution of interphase and SSE properties under repeated compressive and tensile loading. For instance, *operando* nanoindentation could quantify local, time‐dependent mechanical property changes; coupled with AI/ML, these datasets could support digital‐twin models that accelerate identification and design of SSEs optimized for metal anode all‐solid‐state batteries.

#### 6.2.3 Other aspects related to practical considerations

Alkaline and alkaline earth metals are classified as flammable, with a variable degree of severity, and can violently react with oxygen and moisture. Increased reactivity in general tracks with increased surface area, which is currently a common phenomenon observed among the metals reviewed herein with repeated plating and stripping, as was described in Sections 2.4.4, 3.1, and 4.3. The propensity of the metal to form dendritic, globular or porous structures with repeated cycling has been well established as a behavior that poses a serious safety risk that can result in battery short and potentially catastrophic failure. However, what is less addressed is that in some instances, these structures can insidiously form and evolve without necessarily being easily detected just based on the cell performance. For example, as was discussed in Section 3.1, Na dendritic structures can evolve and accumulate without obvious warning signs; this is also true for Mg metal, which has the propensity to form spherical morphologies which quietly accumulate in the separator, leading to an abrupt cell shorting (Section 4.3). Understanding the root causes of this behavior, in addition to developing effective early detection tools are important areas that require more dedicated R&D efforts.

Safety concerns surrounding cell shorts during operation have been studied most extensively, whereas hazards arising during the manufacturing of metal‐anode cells or under storage and idle conditions have received comparatively little attention, despite their potentially catastrophic consequences. For example, in charged anode‐free cells, an internal short circuit caused a violent fire within only 2.6 seconds in the presence of solvents and 1.9 s without them. On the other hand, a typical Li‐ion cell tested under the same conditions only ejected smoke after 4 s of the internal short [523]. The reaction of molten lithium with oxygen released from the NMC811 cathode was identified as a major contributor to this event. However, severe reactivity can arise even with comparatively benign cathodes. A recent study showed that contact between lithium metal and a LiFePO_4_ (LFP) cathode at room temperature, even in the absence of flammable solvents, can trigger a violent thermite‐like reaction [524]. These issues raise serious concerns not only for cell operation, but also for manufacturability and scale‐up, underscoring the need for innovative solutions to mitigate them.

**FIGURE 18:**
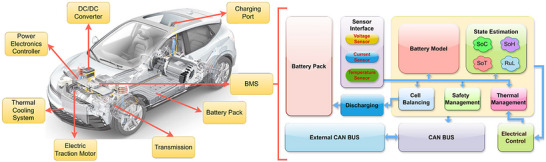
Functional block diagram of the battery management system of an electric vehicle: SoC: State of Charge, SoT: State of temperature, SoH: State of Health, RuL: State of Life, CAN BUS: Controller Area Network communication. Reprinted with Permission [525].

Ultimately, efficient and safe use of battery packs in EVs can only be practically achieved through the use of battery management BMS systems, which are used to diagnose and ensure battery health through performance management, thermal protections, state of charge estimation and fault diagnosis (Figure 18). BMS encompasses a combination of hardware such as sensors and actuators and complex software that includes computational algorithms and real‐time data monitoring [525, 526, 527].

The design of effective BMS relies on capturing battery complex electrochemical interactions in order to design appropriate hardware and software tools capable of maintaining efficient and safe performance. This currently poses a challenge for nascent battery systems due to limitations in understanding and determining these battery responses under real world operation conditions (also see Section 6.1 above). For example, accurate sensing and state estimation are critical, and thus the development of advanced sensors that can allow for in‐situ monitoring and models that can capture the battery aging, aided with data‐driven approaches, is needed. Additionally, development of methods that can be used as early warnings is necessary, for example, as discussed above, Li metal anode batteries have shown a shorter time frame as opposed to Li‐ion preceding the occurrence of a violent event; thus capturing factors that can be used as early warning signs due to thermal or cell fault events is critical.

## Author Contributions

**Chan Shu**: investigation, writing – original draft, visualization, writing – review and editing. **Rana Mohtadi**: conceptualization, investigation, writing – original draft, writing – review and editing, visualization, supervision, resources. **Jian Pan**: investigation, writing – original draft, validation, visualization, writing – review and editing. **Oscar Tutusaus**: investigation, writing – original draft, writing – review and editing.

## Conflicts of Interest

The authors declare no conflicts of interest.

## Data Availability

Data sharing not applicable to this article as no datasets were generated or analysed during the current study.
